# Cestode infection is linked to transcriptional shifts in neuropeptide signalling and caste-specific ageing pathways in a social insect

**DOI:** 10.1186/s12864-026-12959-6

**Published:** 2026-06-15

**Authors:** Giulia Blasi, Katharina Schwolow, Hugo Darras, Susanne Foitzik

**Affiliations:** 1 Institute of Organismic and Molecular Evolution (iomE), Johannes Gutenberg University of Mainz, Mainz, 55128 Germany; 2 Centre for Evolutionary and Organismal Biology, School of Medicine, Zhejiang University, Hangzhou, 310058 China

**Keywords:** Neuropeptides, Ageing, Gene Expression, Parasite Manipulation, Social Insects, Caste

## Abstract

Social insects are key models for phenotypic plasticity, as queens and workers show striking differences in behaviour and lifespan despite sharing the same genome. Neuropeptides are central regulators of behavioural plasticity, and caste-specific ageing is governed by the insulin/insulin-like growth factor 1 signalling–target of rapamycin–juvenile hormone network. Parasites that manipulate host behaviour and lifespan may exploit these regulators by targeting conserved neuropeptides or by altering ageing pathways. Here, we examine a host–parasite system in which the cestode *Anomotaenia brevis* extends lifespan and alters behaviour in its intermediate host, the ant *Temnothorax nylanderi*. In the fat body, queens and infected workers showed a higher degree of overlap in gene expression, consistent with a queen-like transcriptional shift in infected workers. This overlap was characterised by genes involved in ageing regulation, indicating that parasites affect components of conserved metabolic and longevity pathways typically associated with the queen phenotype. Parasite-derived peptides did not resemble host neuropeptides, which argues against classical neuropeptide mimicry based on sequence similarity. The parasite may affect host signalling through alternative mechanisms, including indirect modulation of host neuropeptide pathways. Ant neuropeptide and receptor expression varied strongly with caste and infection, with the latter associated with broad downregulation of neuropeptides in the brain, including tachykinin, short neuropeptide-F, allatostatin-A, orcokinin, CAPA, and diuretic hormones. In contrast, caste-related differences were more pronounced in the fat body, where infected workers resembled uninfected queens. This suggests that infection affects neural and peripheral regulatory pathways, reducing worker-like signalling and partially activating queen-like metabolic programmes. Our results reveal that parasites can exploit caste-specific plasticity and identify the molecular pathways used to reprogram host phenotypes.

**Supplementary Information:**

The online version contains supplementary material available at 10.1186/s12864-026-12959-6.

## Introduction

Phenotypic plasticity, the ability to modify phenotypic traits in response to internal conditions or external environmental cues, is a key factor underlying the ecological and evolutionary success of organisms. In social insects, queens and workers often develop from the same genetic background but differentiate into adults with different behaviours and lifespans through caste-specific gene expression. Comparative analyses across social insects indicate that shifts in central pathways, including insulin/insulin-like growth factor 1 signalling (IIS), target of rapamycin (TOR), and juvenile hormone (JH), are closely associated with the regulation of longevity and fecundity in queens [1]. In ants specifically, insulin signalling has been shown to promote reproduction via the MAPK (mitogen-activated protein kinase) pathway, while reduced AKT (protein kinase B) activity is associated with enhanced longevity in the long-lived reproductive caste [2]. Consistent with this, fertile ant workers that activate their ovaries following queen removal are not only more fecund and long-lived, but also upregulate TOR-pathway genes, indicating involvement of nutrient-responsive signalling during fertility induction [3]. In addition, elevated JH levels during larval development can redirect worker-destined brood toward queen-like phenotypes in adulthood [4, 5].

In addition to life-history traits, queens and workers of social insects exhibit different behavioural repertoires, with the former specialising in reproduction and the latter focusing on brood care, foraging, nest construction and defence. Neuropeptides are short peptides of 3 to 100 amino acids [6] that activate G protein-coupled receptors (GPCRs) in the peripheral and central nervous systems and are important regulators of these social behaviours [7, 8]. Well-studied peptide families, including corazonin, tachykinin-related peptides, insulin-like peptides, short neuropeptide F, and oxytocin/vasopressin-related peptides, together with their receptors, control the physiology and behaviour of social insects, linking internal metabolic states with colony demands [8, 9]. Among these, tachykinin (TK) is linked to aggression and social interactions [10], whereas short neuropeptide F (sNPF) regulates feeding, locomotion, and foraging motivation [11]. Corazonin (CRZ) controls stress and social behaviour [12, 13], and inotocin (INO) governs cuticular hydrocarbon synthesis and water balance in ants [14].

As neuroendocrine pathways are major determinants of lifespan and behaviour, they represent prime targets for exploitation by parasites aimed at altering host phenotypes. Parasites can induce extended phenotypes in their hosts that enhance the parasite’s fitness while imposing costs on the host [15]. For example, they can manipulate host physiology and behaviour through targeted interference with the neuroendocrine pathway [16]. In the jewel wasp *Ampulex compressa*, venom injected into the brain of a cockroach hosts induces a reversible state of lethargy that suppresses escape responses while leaving other motor functions intact; the venom delivers precursors of neuropeptides such as TK and CRZ, which are processed in the host brain to modulate neural circuits controlling locomotion, thereby allowing the wasp to subdue its host for larval development [17].

Because social insects exhibit high plasticity in both lifespan and behaviour, we propose that their neurocircuitry may be especially prone to manipulation by parasites. The acorn ant *Temnothorax nylanderi*, which serves as an intermediate host to the parasitic cestode *Anomotaenia brevis*, provides a tractable system to investigate these mechanisms. Workers infected by *A. brevis* exhibit a markedly prolonged lifespan that matches that of queen ants, likely facilitating transmission to the definitive hosts, woodpeckers [18]. In addition, infected workers, like healthy queens, display reduced activity and flight behaviour, which may further increase their susceptibility to avian predators that access nest sites in rotten wood or acorns [18, 19]. Cestode-infected workers resemble uninfected queens in several traits, indicating that the parasite may exploit the host’s caste plasticity to transform workers into pseudo-queens. Indeed, there is some transcriptional overlap in the gaster between infected workers and healthy queens [20], but this study sequenced only pooled abdomens, providing limited depth and no tissue specificity. However, bulk gaster transcriptomes may fail to resolve relevant molecular pathways, because tissue composition differs between castes; in particular, ovarian tissue constitutes a much larger proportion of the queen’s abdomen. Consistent with a potential mechanistic influence on the host, cestode cysticercoid larvae are transcriptionally active within the haemolymph of the host ant and release proteins, including antioxidants and epigenetic regulators like thioredoxin peroxidase, calreticulin, superoxide dismutase and peptidyl-prolyl cis-trans isomerase [21]. However, whether these parasites specifically target ageing pathways or neuropeptide signalling remains unknown.

Here, we test whether the transcriptional activity of infected workers resembles that of healthy queens. We focus on the molecular underpinnings of two key traits—lifespan and behaviour—with particular emphasis on neuropeptides known to modulate behavioural phenotypes. We compare gene expression across castes and infection states to identify candidate points of interaction with parasite-derived factors. We addressed previous limitations from bulk transcriptome studies by providing substantially higher resolution with tissue-specific transcriptomes of the brain and fat body. We focused on the brain and fat body since they represent key regulatory hubs for parasite-induced plasticity: the brain is central to neuropeptide signalling and behavioural regulation [22, 23], whereas the fat body coordinates endocrine, metabolic, and immune processes [24, 25] that may also be altered by infection. We hypothesised that infection would shift host neuropeptide expression towards a queen-like profile, consistent with parasite-induced changes in caste-related physiology, and that these effects would be most pronounced in the brain. We further investigated whether cestode-derived peptides share sequence similarity with host neuropeptides, suggesting potential molecular mimicry.

## Materials and methods

### Field collection and sample preparation

Colonies of *T. nylanderi* were collected in August–September 2023 in the Lenneberg Forest near Mainz, Germany (50.0116 N, 8.1749 E), under permit from the local forestry office. Colonies of this species are monogynous and, at this site, approximately one quarter of all colonies contain workers infected with the cestode *Anomotaenia brevis*. These infected workers can be reliably recognised by their pale pigmentation and, on average, account for 13% of the workforce [19]. In some cases, infected virgin queens are produced, but they never leave the colony, remain unmated, and exhibit a short lifespan, which is typical for virgin queens in ants [26, 27]. The ants were maintained under controlled conditions (21 °C, 60% RH, 14:10 h light: dark cycle) with water, honey, and crickets provided weekly. We dissected brains and fat bodies from pools of single queens and pools of either two infected or two uninfected workers from seven colonies (*N* = 1 replicate per type per colony). Infected and uninfected workers were collected from the same seven colonies, while queens were collected from seven additional infected colonies from the same site, using RNA-seq data previously generated as part of an independent study conducted alongside whole-genome bisulfite sequencing (WGBS) [28]. Samples were preserved in TRIzol at − 80 °C until extraction. RNA extraction was conducted with the RNeasy Mini Kit (Qiagen). Libraries were prepared using the NEBNext Ultra RNA Library Prep Kit and sequenced on a Novaseq X Plus Series (PE150bp, ~ 9.5 to 30.9 million reads per sample). These transcriptome data were recently used to investigate their association with DNA methylation patterns; however, the study did not include a detailed analysis of the expression patterns themselves (BioProject PRJNA1297301; [28]). In addition, we used published cestode RNA-seq data to identify neuropeptides (BioProject PRJNA950591; [29]).

### Gene expression analysis

We examined transcriptomic variation across castes, tissues, and infection states in *T. nylanderi* by analysing 42 RNA-seq libraries from brains and fat bodies of queens, infected workers, and uninfected workers from infected colonies and 10 cestode RNA-seq libraries (BioProject PRJNA950591; [29]). Ant reads were quality-trimmed with Trimmomatic v.0.39 [30] and aligned to the *T. nylanderi* genome (GCA_048541665.1) [31] using STAR v.2.7.3a [32]. Gene counts were generated with featureCounts v.2.0.6 [33], and differential expression was tested in DESeq2 v.1.44.0 (R v.4.4.1) [34] using Wald tests with Benjamini–Hochberg correction to identify differentially expressed genes between different categories. To minimise statistical bias, we excluded genes for which the total read count was below 10 in the 6 lowest-expressing samples out of 7 for queen-worker and infection comparisons. Co-expression patterns of candidate neuropeptide genes and candidate receptor genes were analysed separately in brain and fat body using the *degPatterns* function in DEGreport v.1.40.1 [35]. Normalised expression values were clustered across the three treatment groups, with nClusters = 3 for neuropeptides, nClusters = 2 for receptors, and a minimum cluster size of three genes (minc = 3). Transcript reconstruction was performed with StringTie v.2.2.3 [36] guided by the GAGA annotation to validate existing gene models. Coding sequences were predicted with TransDecoder v.5.7.1 [37], retaining open reading frames with more than 3 amino acids. Differentially expressed gene (DEG) overlap was assessed separately for each tissue using Fisher’s exact tests. Genes were classified as differentially expressed based on pairwise DESeq2 contrasts (p_adj_ < 0.05) in uninfected queens, infected workers, and uninfected workers. We identified upregulated genes in each category and tested whether pairwise overlaps deviated from random expectation using Fisher’s exact tests. For each comparison, we constructed contingency tables based on the number of genes upregulated in each category, the number shared between categories, and the number remaining in the background set. The background set comprised all genes upregulated in at least one of the three categories within a given tissue and defined the gene universe for these tests.

To functionally annotate the shared DEGs between uninfected queens and infected workers, genes were matched to insect homologues using BLASTp v.2.12.0 [38]. against the insect proteome from NCBI RefSeq (assessed October 2025). For each query, the best hit was retained, provided it had a minimum e-value of <1e–5 and ≥ 50% query coverage. When the top hit was annotated as an uncharacterized protein, sequences were manually re-checked with the online NCBI BLASTp tool [38] against the non-redundant (nr) protein database to identify the most likely functional match. The names of the closest *Drosophila melanogaster* homologues were used to search the literature for functional studies, particularly those addressing ageing (Supplementary File 2). In addition, Gene Ontology (GO) enrichment analyses were conducted using the goseq package [39] with GO annotations derived from eggNOG-mapper v6.0 [40] via the publicly available web server (http://eggnog-mapper.embl.de, accessed February 2025), using tissue-specific sets of differentially expressed genes (p_adj_ < 0.05) as input.

### Neuropeptide and receptor functional characterisation

Although neuropeptides are highly conserved, automated genome annotations often leave them incompletely predicted or inconsistently named, so functional re-annotation was required to obtain a reliable and comparable set across ants and cestodes. Orthologous relationships were inferred with OrthoFinder v.3.0.1b1 [41] using proteomes from *T. nylanderi*, a panel of ants and bees, and *Drosophila melanogaster* (UniProt; accessed December 2024; see Supplementary File 4). Cestode neuropeptides and receptors were annotated by comparison with cestodes and *Caenorhabditis elegans* proteomes (UniProt; accessed February 2025; see Supplementary File 5). Literature-curated peptide sequences were retrieved with PANZZER v.3 [42]. to obtain GO-based functional information, which allowed us to verify their expected neuropeptide roles and to reliably recover already annotated peptide families for comparison with the predicted *A. brevis* and *T. nylanderi* proteomes. Candidate neuropeptide receptors were identified from orthogroups and screened for transmembrane domains with TMHMM v.2.0 [43]. Peptide processing features were predicted with ProP v.1.0 (cleavage sites [44]), and SignalP v.3.0 (signal peptides [45], , while conserved domains were identified with InterProScan v.5.71 [46]. Candidate sequences were validated using the online NCBI BLASTp tool [38] against the non-redundant (nr) protein database, and by comparison to curated insect neuropeptide datasets to confirm homology and annotation consistency.

### Parasite–host peptide similarity

To compare cestode-derived peptides with ant neuropeptides, we extracted predicted proteins from the cestode genome (GCA_030710315.1) and annotation [47] using gffread v.0.12.7 [48]. To identify cestode proteins secreted in the host´s haemolymph, we BLAST haemolymph proteins identified in Hartke et al. (2023) [21] using an older genome assembly version against the new predicted *A. brevis* proteome with BLASTp v.2.12.0 [38]., retaining the best hit per query. Putative secreted peptides were further filtered with DeepLocPro v.1.0 (score > 0.7) [49], a deep-learning predictor of subcellular protein localisation. Pairwise similarity to *T. nylanderi* neuropeptides was assessed by constructing sequence alignments with MAFFT v.7 [50], and calculating pairwise sequence similarity scores with the FASTA36 suite v. 36.3.8 g [51]. We acknowledge the use of ChatGPT to assist with English proofreading and script debugging.

## Results

**Fig. 1:**
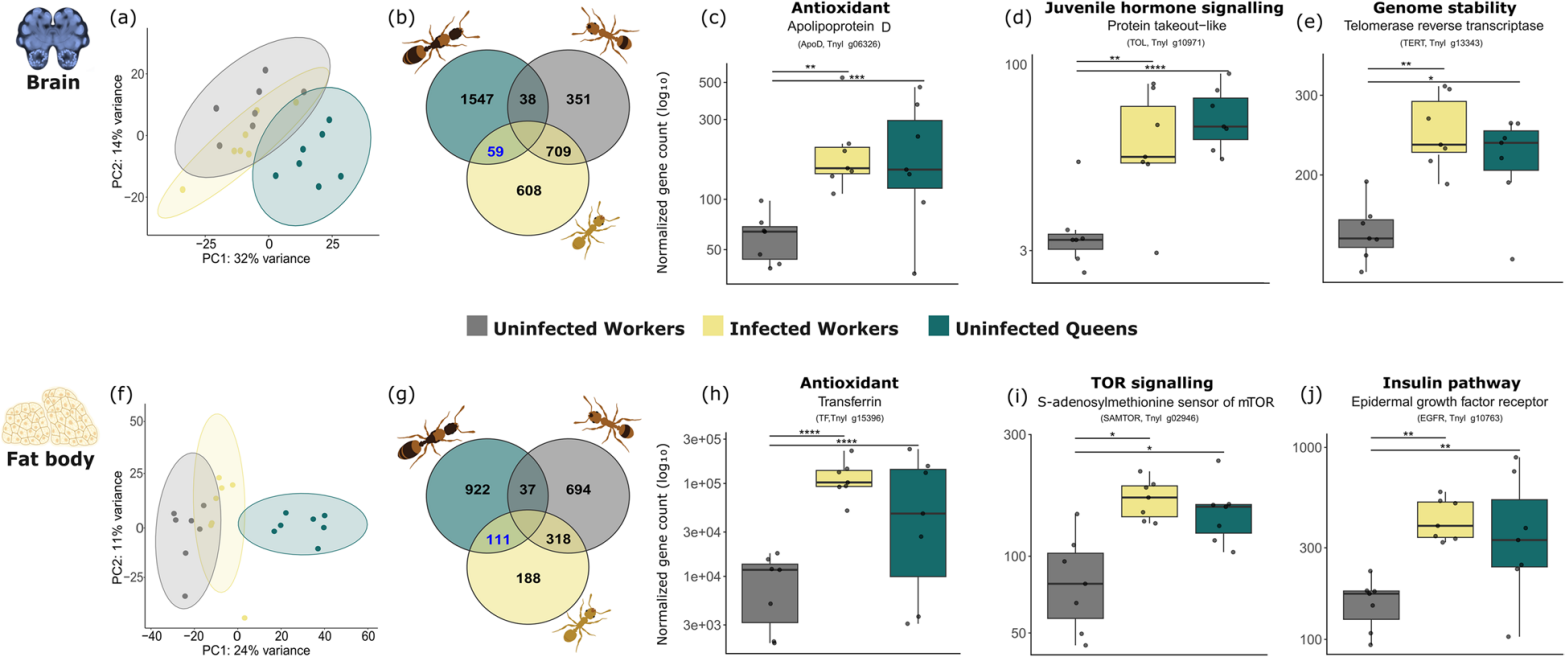
Differential gene expression and transcriptomic variation across castes and infection states in the brain and fat body. Principal component analyses (PCA) (**a**, **f**) show sample clustering for the brain (**a**) and fat body (**f**) based on variance-stabilised RNA-seq expression values. Each point represents one sample: queens (teal), uninfected workers (grey), and infected workers (yellow). Venn diagrams (**b**, **g**) show overlaps among genes upregulated in each group (DEGs; p_adj_ < 0.05, **** p_adj_ < 0.0001) across castes and infection states. Boxplots showing example genes upregulated in both queens and infected workers, linked to ageing pathways in the brain (**c**-**e**) and fat body (**h**-**j)**. Some graphical elements were created with BioRender.com

Global gene expression analyses separated queens from uninfected workers in the tissue-specific principal component analyses (PCA). In the fat body, infected workers occupied an intermediate position between queens and uninfected workers, consistent with our prediction that infection induces a partially queen-like transcriptional profile. This pattern was captured along the first principal component (PC1), which represented the main caste and infection-related axis of variation, explained 24% of the total variance and was associated with genes enriched for functions related to muscle function, ion transport, and membrane potential regulation (top 500 PC1-contributing genes; Fig. 1a; Supplementary File 1). In the brain, PC1 accounted for 32% of the variance and was associated with genes enriched for functions related to signalling, stimulus response, and nervous system-related processes (top 500 PC1-contributing genes; Fig. 1f; Supplementary File 1). However, separation among groups was weak and not clearly resolved along this or subsequent components, indicating that brain variation was more heterogeneous and not structured by the same caste- and infection-related axis observed in the fat body.

To identify group-specific transcriptional differences, we next analysed DEGs. In the brain, uniquely upregulated genes, defined as genes with higher expression in one group relative to both others, were most abundant in uninfected queens (1,547), followed by infected workers (608) and uninfected workers (351). When focusing on genes commonly upregulated across groups, the greatest overlap was observed between the two worker groups (709 genes). The other two pairwise overlaps were substantially lower, with 59 genes shared between uninfected queens and infected workers and 38 genes shared between uninfected queens and uninfected workers. This pattern is consistent with the weak group separation observed in the brain PCA (Fig. 1b). 

In the fat body, queens exhibited 922 exclusively overexpressed genes, followed by uninfected workers (694) and infected workers (188). Across pairwise comparisons, 318 DEGs were shared between the two worker groups, 111 between uninfected queens and infected workers, and 37 between uninfected queens and uninfected workers (Fig. 1g). DEG sharing was non-random and strongly structured by caste and infection status, as supported by Fisher’s exact tests using, for each tissue, a background defined as the union of genes that were differentially expressed in at least one of the three categories. In the fat body, overlap between genes upregulated in the two worker types was significantly enriched (worker types: odds ratio = 1.34, *p* = 0.0021), whereas overlaps involving uninfected queens were significantly depleted, although depletion was weaker between uninfected queens and infected workers than between uninfected queens and uninfected workers (uninfected queens–infected workers: odds ratio = 0.16, p < 2.2 × 10⁻¹⁶; uninfected queens–uninfected workers: odds ratio = 0.0067, *p* < 2.2 × 10⁻¹⁶). These patterns are consistent with a relative shift toward queen-like gene expression in infected workers compared with uninfected workers. 

Next, we focused on those genes which were commonly upregulated in infected workers and healthy queens. The overall expression profiles showed clear tissue-specific trajectories. In the fat body, the 111 commonly upregulated genes include genes associated with immunity, metabolism, and ageing (Fig. 1h-j), reflecting core physiological processes affected by infection and caste (Supplementary File 2). Among these, we found prominent immune effectors comprising lysozymes, and scavenger receptors, together with proteases and transporters linked to detoxification and pathogen recognition. Metabolic and longevity-related genes such as trehalose transporter, α-glucosidase, and chaperonins further supported metabolic and ageing-related modulation (Supplementary File 2). In addition, the fat body showed convergent upregulation of stress-response and maintenance factors, including transferrin (TF; [52]; Fig. 1h), S-adenosylmethionine sensor upstream of mTORC1 (SAMTOR [53], ; Fig. 1i), and epidermal grow factor (EGFR; [54]; Fig. 1j) suggesting modulation of antioxidation, insulin/IGF (IIS) and juvenile hormone (JH) pathways, consistent with findings by Stoldt et al. [20].

In contrast, the 59 genes commonly upregulated in the brain of queens and infected workers were enriched for metabolic and translational components, including ribosomal subunits, translation factors, and oxidative stress-related enzymes such as α-tocopherol transferase and uricase-like protein. This pattern aligns with the expectation that brain transcription largely reflects changes in cellular maintenance and metabolic regulation rather than immune activation (Supplementary File 2). Additional differential expressions of ribosomal genes, together with stress-associated antioxidant and neuroprotective factors, including Apolipoprotein D (ApoD) [55] (Fig. 1c), take-out like (TOL) [56] (Fig. 1d), and telomerase reverse transcriptase (TERT) [57] (Fig. 1e), are consistent with the activation of conserved stress-resistance and translational control mechanisms. However, whether the parasite may mediate these changes directly through neuronal signalling, interfering with the host system or indirectly, cannot be assessed from the present transcriptome data alone. However, only thirteen genes were commonly upregulated in queens and infected workers in both the brain and the fat body, consistent with clear tissue-specific trajectories. These include eleven characterised genes (Ankyrin, Cytochrome P450, two Peptidase M1 paralogs, TERT, transferrin, 4-coumarate–CoA ligase 1-like, condensin complex subunit 1, protein takeout-like, protein artichoke-like, and α-glucosidase) and two uncharacterized ones (Supplementary File 2), however, no significant or biologically informative GO enrichment was detected for either tissue (Supplementary File 3).

Because many of the transcriptional differences involved pathways linked to behaviour, metabolism, and ageing, we specifically examined the neuropeptide system, a major regulator of these processes. We identified 34 neuropeptides in the *T. nylanderi* genome. Of these, 32 had existing functional annotations in *T. nylanderi*. CAPA (capability peptide) and ITG (ion transport peptide) lacked prior functional annotation and were assigned based on homology in this study. Both CAPA and ITG showed > 97% identity to the corresponding proteins in the NCBI *Temnothorax curvispinosus* annotation (ASM307098v1) and clustered with Formicinae in phylogenetic analyses. Because these two peptides were newly annotated, we specifically report their expression patterns, showing expression in both brain and fat body with multiple isoforms detected (Supplementary File 4). CAPA expression was highest in uninfected workers, compared to infected ones (p_adj_ = 0.01) and queens (p_adj_ = 8 × 10⁻^8^), while infected workers and queens did not differ (p_adj_ = 0.07). ITG expression peaked in queens, with lower levels in infected workers (p_adj_ = 0.01); other contrasts were not significant (p_adj_ > 0.05). Of the 34 neuropeptides, 25 were actively expressed in both tissues (see Methods for expression thresholds). Their expression was generally higher in the brain than in the fat body and only weakly correlated between tissues (LM: slope = 0.41, R² = 0.15, *p* = 0.054; Supplementary Figs. 1).

Annotation of neuropeptide receptors in *T. nylanderi* revealed 23 candidates, including several receptors for which we corrected or refined the functional annotation based on updated sequence similarity searches and domain analyses. Within this set, we identified an Adipokinetic Hormone (AKH) receptor and two isoforms of the Pyrokinin 1 receptor and re-annotated a previously ambiguous receptor (DAR2) as an Allatostatin-A (AstA) receptor based on BLASTp searches. Because several candidates represented duplicated copies of the same receptor type, these paralogues were grouped into receptor families for structural annotation, yielding 18 distinct receptor loci, although each paralogue was retained separately in the gene-expression analyses. Among these 18 candidate receptors, 13 displayed the canonical seven transmembrane helices and were annotated as GPCRs. The remaining five receptor candidates (Diuretic Hormone Receptor, Tachykinin-like 99D, Tachykinin-like 86 C, Chamid/NmB, and Prokineticin Receptor 2) were retained in the dataset despite deviating from the expected number of transmembrane helices [58]. This may reflect incomplete transcript models, isoform variation, or alternative splicing rather than loss of function. Because these candidates showed similarity to established neuropeptide receptor families, they were retained as putative receptors. However, pseudogenization cannot be fully excluded, and their functionality remains to be confirmed. Expression of the 18 (see Methods for expression thresholds) actively expressed receptors showed no correlation between brain and fat body (LM: slope = 0.13, R² = 0.01, *p* = 0.71, Supplementary Figs. 2).

**Fig. 2:**
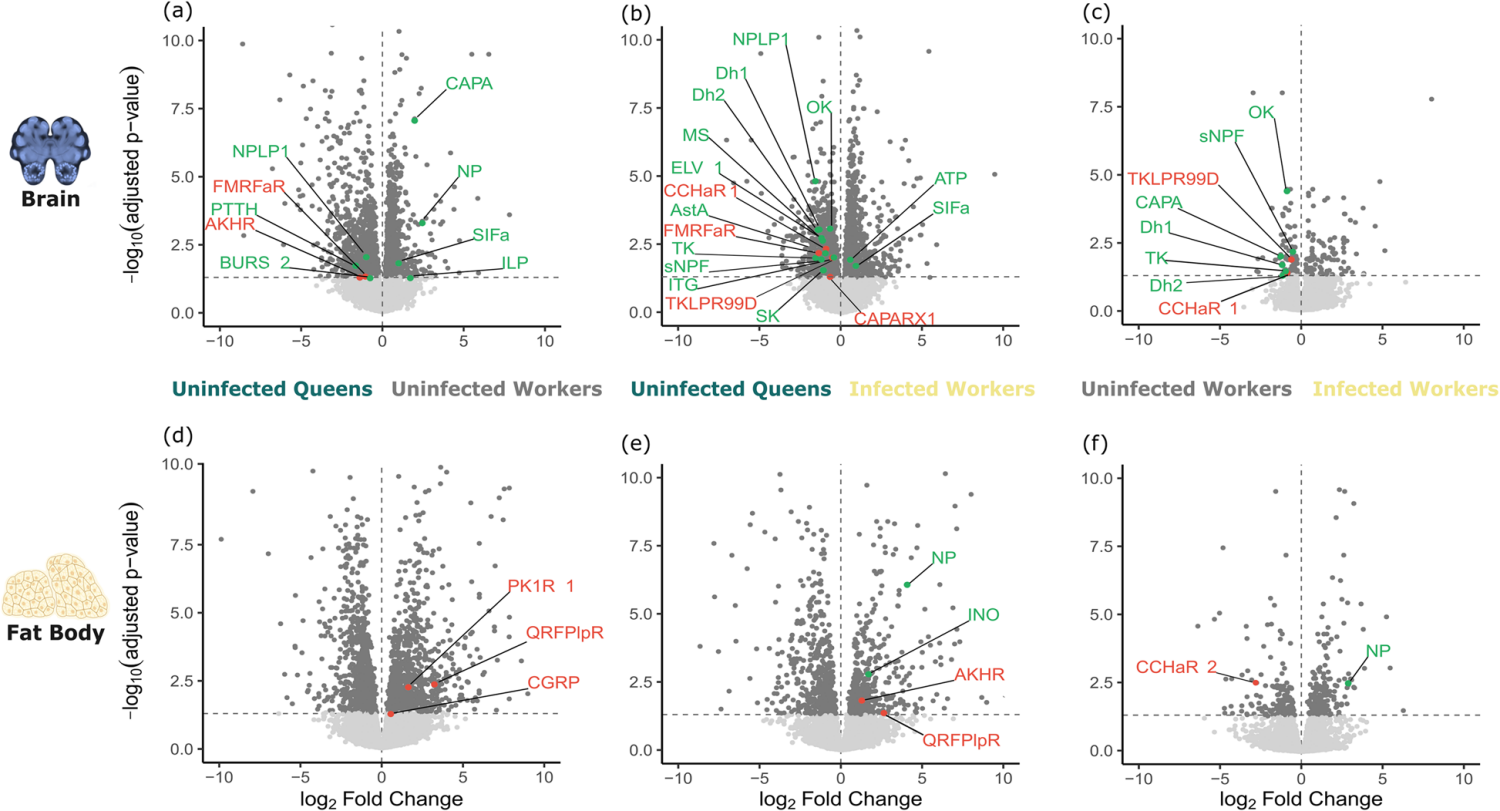
Differential expression of neuropeptides and their receptors in the brain and fat body. Volcano plots (**a**–**c**, **d**–**f**) highlight DEGs, with red points indicating differentially expressed neuropeptide receptors and green points indicating differentially expressed neuropeptides (padj < 0.05): (**a**) queens vs uninfected workers, (**b**) queens vs infected workers, (**c**) uninfected vs infected workers in the fat body; (**d**) queens vs uninfected workers, (**e**) queens vs infected workers, and (**f**) uninfected vs infected workers in the brain. Some graphical elements were created with BioRender.com

**Fig. 3:**
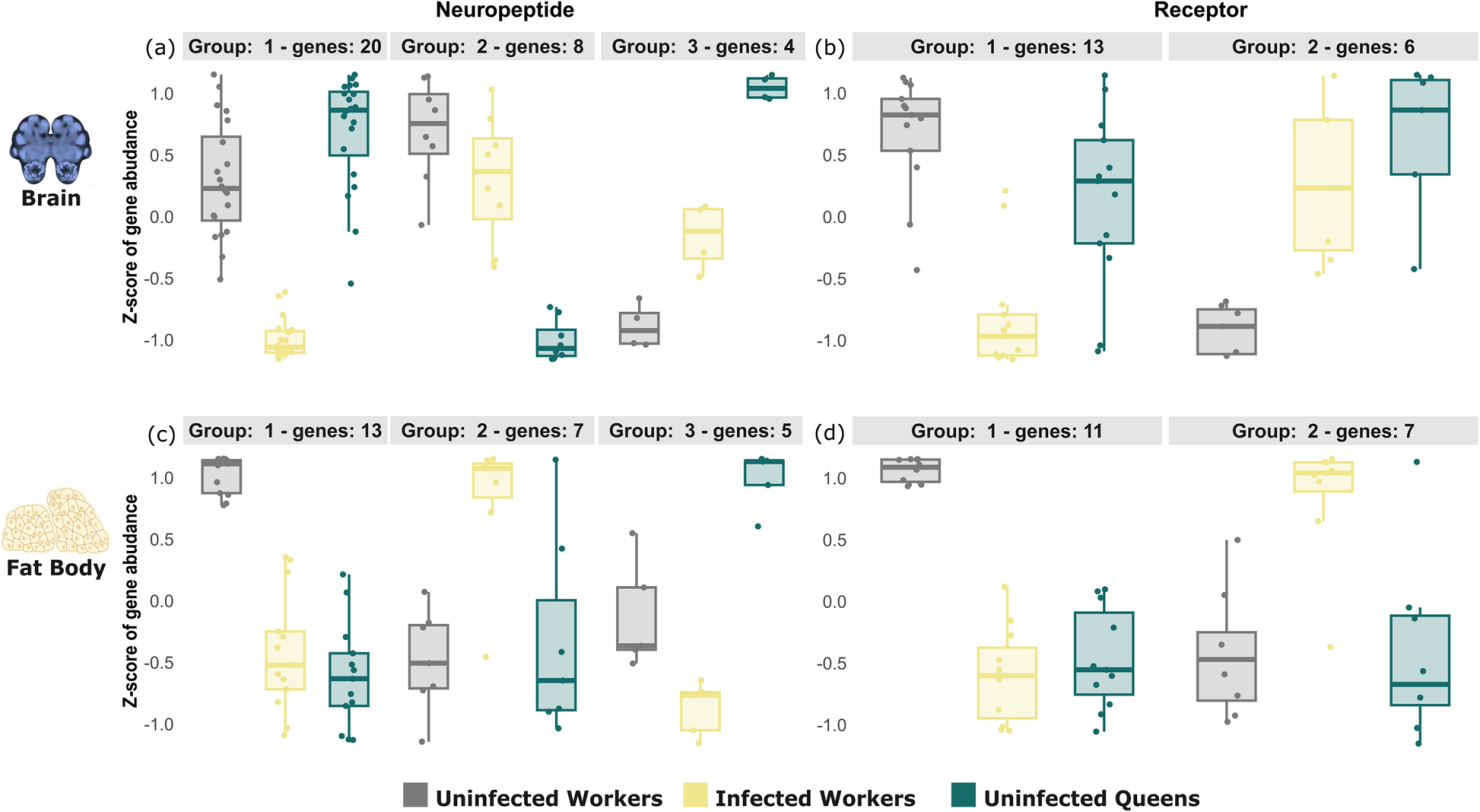
Expression profiles of neuropeptides and their receptors in the brain and fat body. Boxplots display clustered expression patterns of neuropeptides (**a**-**c**) and receptors (**b**-**d**) across castes and infection states in two tissues. Colours indicate caste and infection status: queens (teal), uninfected workers (grey), and infected workers (yellow). Some graphical elements were created with BioRender.com

Clustering of brain neuropeptide expression patterns resolved three groups: the largest cluster comprised 20 neuropeptides that showed low expression in infected workers and higher levels in uninfected workers and queens; a second cluster showed high expression of eight genes in queens; and a third displayed intermediate expression of four genes in infected workers (Fig. 3a). Within the first group, most neuropeptides were significantly downregulated in infected workers, including TK, orcokinin (OK), sNPF, AstA, CAPA, and the diuretic hormones Dh1 and Dh2 (p_adj_  < 0.05; Fig. 2b-c). Pairwise caste contrasts further showed that queens differed from uninfected workers in seven neuropeptides (CAPA, Neuroparsin (NP), SIFamide (SIFa), Neuropeptide-like precursor 1 (NPLP1), Insulin-like peptide (ILP), Bursicon subunit 2 (BURS2), and Prothoracicotropic hormone (PTTH)) (Fig. 2a). Receptor expression in the brain revealed a similar structure: clustering identified two groups, one consisting of 13 genes that were largely downregulated in infected workers and another with 6 genes where infected workers resembled queens (Fig. 3b). Four receptors were significantly differentially expressed in the comparison between queens and infected workers, all showing higher expression in queens. These included Tachykinin receptor 99D (TKR99D; p_adj_ = 0.01), FMRFamide receptor (FMRFaR; p_adj_ = 0.006), CCHamide/Neuromedin B receptor (CCHaR/NmBR; padj = 0.004), and CAPA receptor-like X1 (CAPARX1; p_adj_ = 0.047) (Fig. 2b). In summary, infected workers exhibited a mixed brain profile: broad neuropeptide downregulation and a distinct cluster structure, yet partial resemblance to queens for a subset of receptors.

Fat body clustering supported this pattern: the major neuropeptide group (13 genes, including NP) and the corresponding receptor group (11 genes) both displayed queen-like expression in infected workers (Fig. 3c-d). Only the neuropeptides NP and INO were differentially expressed, both upregulated in infected workers relative to queens (p_adj_ = 8.59 × 10⁻⁷ and 0.002, respectively; Fig. 2e). NP showed opposite regulation across tissues, being higher in uninfected workers in the brain (padj = 5 × 10⁻³; Fig. 2a), but higher in infected workers in the fat body (padj =8.59 × 10⁻⁷ and 0.0037; Fig. 2e-f). Together, these results show that infection reshapes neuropeptide signalling in a strong tissue-specific manner, suppressing many brain neuropeptides and receptors while shifting fat body expression in infected workers toward a more queen-like profile.

Finally, we functionally annotated six candidate cestode neuropeptide families (Supplementary File 5). Four were single-copy, while the remaining families consisted of duplicated genes (g10556/g10560 and g9037–g9039). None were predicted to be secreted in ant haemolymph based on proteomic analyses [21]. Although mature neuropeptides are typically too small to detect by standard proteomics, their larger preproprotein precursors would be identifiable if secreted. Finally, we tested whether any cestode neuropeptides may be mimicking those of the host for manipulation. Remarkably, neither the cestode candidate neuropeptides nor any previously reported secreted proteins showed significant similarity to *T. nylanderi* neuropeptides (MAFFT/FASTA; > 30% identity).

## Discussion

Our results show that infection by the cestode *A. brevis* profoundly alters host gene expression in *T. nylanderi*, shifting the molecular profile of infected workers toward that of queens in a tissue-specific manner. This shared transcriptional pattern was strongest in the fat body, where infected workers and queens shared a limited but biologically meaningful set of differentially expressed genes, including genes associated with longevity, reproduction, and stress resistance [59]. Some of these genes are associated with insulin/IGF, TOR, and juvenile hormone ageing pathways, suggesting that infection may affect components of molecular programs also linked to queen physiology. Elevated expression of immune- and stress-related genes in queens may not reflect higher direct pathogen exposure, as queens experience a socially protected environment, but may instead represent a constitutive protective and maintenance physiology associated with extended lifespan [60, 61], consistent with the remodeling of endocrine and physiological trade-offs in social insects [62]. These transcriptional shifts also involve several neuropeptides and their receptors, which may underlie the reduced activity observed in both infected workers and queens. In contrast, similarity in the brain was much weaker: only ~ 12% of neuropeptide- and receptor-related DEGs showed similar changes in queens and infected workers, and overall expression patterns remained distinct, suggesting that any queen-like shift in the brain is limited and that parasite-induced effects on brain regulation may also involve mechanisms beyond caste-related pathways. However, this pattern was much stronger in the fat body than in the brain, where expression changes were more heterogeneous and overlap between queens and infected workers was lower. This weaker and more variable pattern in the brain may reflect both a limited queen-like component of the parasite-induced response and the cellular complexity of this tissue, where bulk transcriptomic profiles integrate distinct changes across multiple cell types.

The queen-like traits and associated transcriptional shifts observed in infected workers appear to result from the cestode infection. Nevertheless, some differences may reflect age-related variation among queens, infected workers, and uninfected workers. An ongoing study comparing recently emerged and older infected and uninfected workers allowed us to control for age and revealed strong effects of infection alone and in interaction with age on tissue-specific gene expression (Chitadze et al., *unpubl. data*). Because infection prolongs worker lifespan, infected individuals are likely older than their uninfected nestmates [18]. This increased age may partly explain altered fat body transcription, but the reproductive benefits and metabolic buffering cannot be attributed to age alone. Age-related adjustments could explain the upregulation of IIS, TOR, and JH pathway components, as well as the enrichment of chaperonins and detoxification enzymes, observed in both queens and infected workers. These pathways are central to longevity regulation in insects [2, 63, 64], suggesting that the parasite either co-opts or reinforces existing ageing mechanisms to stabilise host metabolism and prolong lifespan. This infection-associated shift was evident in both tissues but followed distinct trajectories. In the fat body, infection was associated with activation of pathways related to cellular maintenance, stress tolerance, and longevity, consistent with a more queen-like metabolic state. In contrast, the brain showed transcriptional signatures associated with general stress resistance and translational control rather than a clear modulation of neural signalling pathways. These tissue-specific responses suggest that infection primarily engages conserved ageing and metabolic networks and stabilises infected worker physiology in a manner that partially resembles the queen phenotype without recapitulating queen-like neuronal profiles.

A core set of behaviourally relevant neuropeptides was suppressed in infected workers, and these did not overlap with queen-worker differentially expressed genes. TK, which modulates aggression, locomotion, immunity and feeding in insects [7, 65, 66], was consistently downregulated in infected ants, together with its receptor (TkR99D). The same pattern was reported by Feldmeyer et al. (2016) [67], suggesting that tachykinin signalling is an important target of parasite manipulation. Similarly, the transcriptional activity of sNPF, a key regulator of feeding and energy balance [11, 68, 69], was consistently reduced in infected worker brains, supporting parasite-induced suppression of foraging activity. Its low or undetectable expression in the fat body is consistent with predominantly neural expression. Finally, OK, a poorly characterised neuropeptide linked to reproduction and social behaviour in insects [70, 71], showed a stable reduction in expression in infected workers, possibly contributing to their intermediate reproductive phenotype [18].

Multiple diuretic hormone pathways were consistently downregulated in infected workers. CRF/DH and the calcitonin-like peptide DH31, which regulate satiety, diuresis and reproduction [72], were both significantly reduced. CAPA peptides, which control ion–water balance, stress tolerance and reproduction via their GPCR receptor [73, 74], showed the same trend. Because these peptides act as satiety factors and inhibitors of oogenesis in other insects, their suppression may promote increased feeding and reproductive investment. The coordinated decline of these pathways may weaken satiety signalling and alter fluid and energy homeostasis, thereby promoting nutrient intake and reallocating resources towards reproduction.

Neuroparsin (NP) showed a more complex, tissue-specific pattern. Queens expressed lower levels than workers in both brain and fat body, consistent with their inhibitory effect on juvenile hormone and the greater reproductive investment of queens [75–77]. The upregulation of NP in the fat body of infected workers may therefore reflect activation of the same pathway described by Zhang et al. (2024) [77], where NP regulates reproductive plasticity through modulation of juvenile hormone binding protein (JHBP) and the ecdysteroid biosynthesis gene *shadow*, thereby influencing ovary activity. In workers, NP was upregulated in uninfected brains but downregulated in infected ones, suggesting that the parasite suppresses a factor normally reinforcing worker sterility and behaviour. In contrast, NP was elevated in the fat body of infected workers, indicating a shift in metabolic regulation during infection. These patterns suggest that NP acts at the interface of caste differentiation and parasite manipulation. Supporting this interpretation, NP has been linked to division of labour in *Atta cephalotes* (where it programs worker behavioural phenotypes) [78] and to phase transition in the locust *Schistocerca gregaria* [79].

Infected workers showed a pronounced reduction in neuropeptide and receptor expression in the brain, whereas a small subset of peptides was upregulated in the fat body. This indicates that infection affects neuropeptide signalling in a selective and tissue-specific manner, rather than through a uniform system-wide response. In the fat body, the peptides that did increase in expression partly resembled queen-like profiles, whereas brain expression in infected workers consistently diverged from both uninfected workers and queens in ways that reduce worker activity while supporting a more long-lived physiological state [80, 81]. The parasite would benefit from such alterations, as infected hosts would be less likely to be attacked or expelled by nestmates, thereby increasing the chances of successful transmission. Recent studies show that helminths use neuropeptides to mediate communication, potentially influencing host physiology [81]. Similarly, parasitic wasps such as *Cotesia vestalis* manipulate host enteroendocrine cells to reduce lipid production, essential for parasite development, through the upregulation of TK signalling [82].

Beyond this system, it has been reported that parasite infections can influence host phenotypes along pre-existing life-history variation axes, rather than creating new regulatory states [83]. These phenotypic states include extreme life-history traits observed in nature [84, 85] and have been linked to altered octopaminergic [86], serotonergic [87], or neuroendocrine signalling [88] in diverse invertebrate systems, where parasites exploit host-derived neuromodulators to reshape activity, feeding, or reproductive behaviour. Importantly, these shifts are thought to arise not from the direct production of neuromodulators by parasites, but rather as adaptations to evade the host’s immune system [89].

## Conclusions

These findings show that *A. brevis* exploits physiological plasticity in its ant host by recruiting conserved ageing and metabolic pathways. Coordinated shifts in metabolic, immune, and neuropeptidergic signalling are associated with a queen-like, long-lived, and behaviourally inactive phenotype in infected workers. The overlap between caste-related and infection-induced plasticity indicates that parasites can redirect social insect physiology through pre-existing regulatory networks. This system, therefore, provides a tractable model for studying the evolution of host manipulation and the molecular mechanisms underlying social life-history transitions. Coordinated shifts in metabolic, immune, and neuropeptidergic signalling are associated with aspects of a queen-like phenotype in infected workers, particularly in the fat body, whereas brain expression profiles remain more distinct. These findings suggest that infection-associated lifespan extension and behavioural decline may not be mediated by the same mechanisms that underlie queen-like traits. Together, these results show that infection reshapes neuropeptide signalling in a tissue-specific manner, with infected workers showing suppression of many brain neuropeptides and receptors, but a more queen-like expression profile in the fat body.

## Supplementary Information

Supplementary File 1.

Supplementary File 2.

Supplementary File 3.

Supplementary File 4.

Supplementary File 5.

Supplementary File 6.

Supplementary File 7.

Supplementary File 8.

Supplementary Figs. 1.

## Data Availability

All data supporting the conclusions of this study are included within the article and its Supplementary Materials. Sequencing reads are available in the NCBI database under BioProject accession numbers PRJNA1297301 and PRJNA950591.
