# Supplementary material for: Cestode infection is linked to transcriptional shifts in neuropeptide signalling and caste-specific ageing pathways in a social insect
Source: BMC Genomics. 2026 Jun 15;27:547. doi: 10.1186/s12864-026-12959-6 (PMC13267302; doi:10.1186/s12864-026-12959-6)
Supplement: Supplementary file 1 [file 12864_2026_12959_MOESM1_ESM.docx]

**Supplementary Information**

**• Supplementary File 1
Table 1.** Gene-level raw read count matrix for RNA-seq samples from brain and fat body in queens, uninfected workers, and infected workers.

**Table 2.** PC1 and PC2 gene loadings from principal component analyses of fat body and brain transcriptomes.

**Table 3.** Separate GO Biological Process enrichment tables for the top 500 genes contributing to PC1 in the fat body and brain. **Table 4.** Differential expression results from DESeq2 for all pairwise caste and infection-status comparisons, analysed separately for brain and fat body.

**• Supplementary File 2
Table 1.** BLAST-based annotation of *Temnothorax nylanderi* brain-expressed proteins commonly upregulated in infected and uninfected workers versus queens. **Table 2.** BLAST-based annotation of *T. nylanderi* fat body-expressed proteins commonly upregulated in infected and uninfected workers versus queens.

**• Supplementary File 3
Table 1.** Gene Ontology (GO) Biological Process enrichment analysis of genes commonly upregulated in infected and uninfected workers versus queens in the brain. **Table 2.** Gene Ontology (GO) Biological Process enrichment analysis of genes commonly upregulated in infected and uninfected workers versus queens in the fat body.

**• Supplementary File 4
Table 1.** Neuropeptides and neuropeptide receptors´ abbreviations used throughout the manuscript and supplementary materials. **Table 2.** Orthogroup composition across ant species (A), bee species (B), and outgroup taxa used for comparative analyses, including *Drosophila melanogaster* and proteomes derived from the *T. nylanderi* genome annotation and TransDecoder-based transcriptome assemblies. **Table 3.** Functional annotation of neuropeptides and neuropeptide receptors identified in *T. nylanderi*.
**Table 4.** Predicted transmembrane domain architecture of neuropeptide receptors identified in *T. nylanderi*.

**• Supplementary File 5
Table 1.** Orthogroup composition across cestode species, with *Caenorhabditis elegans* and *T. nylanderi* as outgroups, and *Anomotaenia brevis* TransDecoder-based transcriptome assembly. **Table 2.** Neuropeptide- and receptor-associated orthogroups of *A. brevis* across cestodes, including *C. elegans* and *T. nylanderi*. **Table 3.** Annotated neuropeptides and neuropeptide receptors identified in *A. brevis*.

**• Supplementary File 6
Table 1.** Pairwise sequence similarity among neuropeptides from *T. nylanderi*, *A. brevis*, and sequences found in ant haemolymph released by the cestode (Hartke et al., 2023).

**• Supplementary File 7**Quarto HTML report providing a reproducible RNA-seq analysis workflow from raw count processing through differential expression and downstream analyses. All figures were automatically generated within the report and subsequently edited in Inkscape for layout and formatting.

**• Supplementary Figures**This file contains supplementary figures related to neuropeptide and receptor analyses, including expression profiles of newly annotated neuropeptides (CAPA and ITG) in brain and fat body, tissue-specific heatmaps of neuropeptide and receptor expression, and clustered expression patterns with labelled genes. In addition, it includes comparative orthogroup distributions across ants, other social insects, and cestode species.
