# Supplementary material for: Cestode infection is linked to transcriptional shifts in neuropeptide signalling and caste-specific ageing pathways in a social insect
Source: BMC Genomics. 2026 Jun 15;27:547. doi: 10.1186/s12864-026-12959-6 (PMC13267302; doi:10.1186/s12864-026-12959-6)
Supplement: Supplementary file 8 [file 12864_2026_12959_MOESM8_ESM.html]

Cestode Infection Is Linked to Neuropeptide Signalling and Caste-Specific Ageing Pathways in a Social Insect, Blasi et al., 2026


# Cestode Infection Is Linked to Neuropeptide Signalling and Caste-Specific Ageing Pathways in a Social Insect, Blasi et al., 2026

## Supplementary File 7: RNA-seq analysis pipeline

This Quarto document contains the complete computational workflow used for RNA-seq data analysis of the ant *Temnothorax nylanderi* and its cestode parasite. The pipeline includes all steps from read processing and mapping (Bash), through differential expression analysis in R, to neuropeptide-focused annotation, functional enrichment, and data visualisation.

Analyses are organised in sequential sections reflecting the order of execution. Input files, intermediate objects, and output tables correspond to the datasets generated and analysed in this study.

All analyses were performed in a Linux environment using Bash and R, with standard bioinformatics and Bioconductor packages as specified within the main manuscript.

**Gene Expression Analysis (*Temnothorax nylanderi*)**

```
# -----------------------
# 0) Paths
# -----------------------

#Retrieve the gft from the gff with agat
agat_convert_sp_gff2gtf.pl \
  --gff GAGA-0510_final_annotation_repfilt_addreannot.gff3 \
  --gtf output_old.gtf
  
#########################################################################################################################################################
RAW_DIR="PATH_TO_FASTQ"
WORKDIR="Analysis"
GENOME_FA="GAGA-0510_tnyl_nextpolish_final_dupsrm_filt.softMasked.fasta"
ANNOT_GTF="output_old.gtf"   # ensure GTF (not GFF3) for STAR/featureCounts
THREADS=12
READLEN=130   # set correctly
SJDB_OVERHANG=$((READLEN-1))

mkdir -p "${WORKDIR}"/{01_fastqc,02_trim,03_star,04_counts,05_stringtie,06_transdecoder,07_multiqc}
cd "${WORKDIR}"

# (optional, safer than mv): link raw fastq into project
mkdir -p data
find "${RAW_DIR}" -name "*fq.gz" -exec ln -sf {} data/ \;

# -----------------------
# 1) FastQC + MultiQC (raw)
# -----------------------
mkdir -p 01_fastqc/raw
fastqc data/*.fq.gz -t "${THREADS}" -o 01_fastqc/raw
multiqc 01_fastqc/raw -o 07_multiqc/raw_fastqc

# -----------------------
# 2) Trimming (paired-end) - Trimmomatic
# -----------------------
mkdir -p 02_trim
# expects files like sample_1.fq.gz and sample_2.fq.gz
for R1 in data/*_1.fq.gz; do
  BASE=$(basename "${R1}" _1.fq.gz)
  R2="data/${BASE}_2.fq.gz"

  trimmomatic PE -phred33 -threads "${THREADS}" \
    "${R1}" "${R2}" \
    "02_trim/${BASE}_1P.fq.gz" "02_trim/${BASE}_1U.fq.gz" \
    "02_trim/${BASE}_2P.fq.gz" "02_trim/${BASE}_2U.fq.gz" \
    ILLUMINACLIP:Adapters.fasta:2:30:10 \
    HEADCROP:10 CROP:140 MINLEN:35
done

# QC after trimming
mkdir -p 01_fastqc/trimmed
fastqc 02_trim/*P.fq.gz -t "${THREADS}" -o 01_fastqc/trimmed
multiqc 01_fastqc/trimmed -o 07_multiqc/trimmed_fastqc

# -----------------------
# 3) STAR index + mapping
# -----------------------
mkdir -p 03_star/Genome
STAR --runThreadN "${THREADS}" \
  --runMode genomeGenerate \
  --genomeDir 03_star/Genome \
  --genomeFastaFiles "${GENOME_FA}" \
  --sjdbGTFfile "${ANNOT_GTF}" \
  --sjdbOverhang "${SJDB_OVERHANG}"

mkdir -p 03_star/bam
for R1 in 02_trim/*_1P.fq.gz; do
  BASE=$(basename "${R1}" _1P.fq.gz)
  R2="02_trim/${BASE}_2P.fq.gz"

  STAR --runThreadN "${THREADS}" \
    --genomeDir 03_star/Genome \
    --readFilesIn "${R1}" "${R2}" \
    --readFilesCommand zcat \
    --outSAMtype BAM SortedByCoordinate \
    --outFileNamePrefix 03_star/bam/"${BASE}." \
    --outFilterMultimapNmax 1 \
    --alignSJoverhangMin 8 --alignSJDBoverhangMin 1 \
    --outFilterMismatchNmax 999 --outFilterMismatchNoverLmax 0.04 \
    --alignIntronMin 20 --alignIntronMax 1000000
done

multiqc 03_star/bam -o 07_multiqc/star

# -----------------------
# 4) featureCounts (gene counts)
# -----------------------
mkdir -p 04_counts
featureCounts -T "${THREADS}" -p -C\
  -a "${ANNOT_GTF}" \
  -t CDS --countReadPairs --largestOverlap -g gene_id \
  -o 04_counts/gene_counts.txt \
  03_star/bam/*.bam

# -----------------------
# 5) StringTie (optional transcript assembly/quant)
# -----------------------
mkdir -p 05_stringtie
for BAM in 03_star/bam/*.bam; do
  SAMPLE=$(basename "${BAM}" .bam)
  stringtie "${BAM}" -p "${THREADS}" -G "${ANNOT_GTF}" -o 05_stringtie/"${SAMPLE}".gtf
done

ls 05_stringtie/*.gtf > 05_stringtie/mergelist.txt
stringtie --merge -p "${THREADS}" -G "${ANNOT_GTF}" \
  -o 05_stringtie/merged.gtf 05_stringtie/mergelist.txt

# -----------------------
# 6) TransDecoder (ORFs from merged transcripts)
# -----------------------
mkdir -p 06_transdecoder
gffread 05_stringtie/merged.gtf -g "${GENOME_FA}" -w 06_transdecoder/merged_transcripts.fasta

TransDecoder.LongOrfs -t 06_transdecoder/merged_transcripts.fasta -m 3
TransDecoder.Predict  -t 06_transdecoder/merged_transcripts.fasta

# -----------------------
# 7) OrthoFinder (proteomes). REMEMEBER the "proteomes" folder contains all the proteomes downloaded from Uniprot, the GAGA ones and the 2 ones retrieved from Transdecoder
# -----------------------
orthofinder -f proteomes -t 12

# -----------------------
# 8) PANZZER (proteomes). REMEMEBER the "proteomes" folder contains all the proteomes downloaded from Uniprot, the GAGA ones and the 2 ones retrieved from Transdecoder in order to get the GO associated.
# -----------------------
cd /c/Users/Startklar/Downloads/proteomes

# ---- run PANNZER annotation ----
python runsanspanz.py \
  -R \
  --PANZ_FILTER_PERMISSIVE \
  -m Pannzer \
  -i sans.tab \
  -f tab \
  -o ",DE.out3,GO.out3,anno.out3"
```

**Gene Expression Analysis (*Anamotaenia brevis*)**

```
# CESTODE RNA-seq PIPELINE (paired-end) + ORF prediction + OrthoFinder
# Works for any species: just point GENOME_FA / ANNOT_GTF to the cestode reference
# -----------------------
# 0) Paths (CESTODE)
# -----------------------
RAW_DIR="PATH_TO_CESTODE_FASTQ"
WORKDIR="cestode_analysis"

GENOME_FA="JAUOZQ01.1.fasta"          # cestode reference genome
ANNOT_GTF="JAUOZQ01.1.annotation_BRAKER3-v1.0.gtf"     # GTF (recommended)

THREADS=12
READLEN=150                             # set correctly (e.g., 150)
SJDB_OVERHANG=$((READLEN-1))

mkdir -p "${WORKDIR}"/{01_fastqc,02_trim,03_star,04_counts,05_stringtie,06_transdecoder,07_multiqc,proteomes}
cd "${WORKDIR}"

mkdir -p data
find "${RAW_DIR}" -name "*fq.gz" -exec ln -sf {} data/ \;

# -----------------------
# 1) FastQC + MultiQC (raw)
# -----------------------
mkdir -p 01_fastqc/raw
fastqc data/*.fq.gz -t "${THREADS}" -o 01_fastqc/raw
multiqc 01_fastqc/raw -o 07_multiqc/raw_fastqc

# -----------------------
# 2) Trimming (paired-end) - Trimmomatic
# expects: sample_1.fq.gz and sample_2.fq.gz
# -----------------------
mkdir -p 02_trim
for R1 in data/*_1.fq.gz; do
  BASE=$(basename "${R1}" _1.fq.gz)
  R2="data/${BASE}_2.fq.gz"

  trimmomatic PE -phred33 -threads "${THREADS}" \
    "${R1}" "${R2}" \
    "02_trim/${BASE}_1P.fq.gz" "02_trim/${BASE}_1U.fq.gz" \
    "02_trim/${BASE}_2P.fq.gz" "02_trim/${BASE}_2U.fq.gz" \
    ILLUMINACLIP:Adapters.fasta:2:30:10 \
    HEADCROP:10 CROP:140 MINLEN:35
done

mkdir -p 01_fastqc/trimmed
fastqc 02_trim/*P.fq.gz -t "${THREADS}" -o 01_fastqc/trimmed
multiqc 01_fastqc/trimmed -o 07_multiqc/trimmed_fastqc

# -----------------------
# 3) STAR index + mapping (CESTODE)
# -----------------------
mkdir -p 03_star/Genome
STAR --runThreadN "${THREADS}" \
  --runMode genomeGenerate \
  --genomeDir 03_star/Genome \
  --genomeFastaFiles "${GENOME_FA}" \
  --sjdbGTFfile "${ANNOT_GTF}" \
  --sjdbOverhang "${SJDB_OVERHANG}"

mkdir -p 03_star/bam
for R1 in 02_trim/*_1P.fq.gz; do
  BASE=$(basename "${R1}" _1P.fq.gz)
  R2="02_trim/${BASE}_2P.fq.gz"

  STAR --runThreadN "${THREADS}" \
    --genomeDir 03_star/Genome \
    --readFilesIn "${R1}" "${R2}" \
    --readFilesCommand zcat \
    --outSAMtype BAM SortedByCoordinate \
    --outFileNamePrefix 03_star/bam/"${BASE}." \
    --outFilterMultimapNmax 1 \
    --alignSJoverhangMin 8 --alignSJDBoverhangMin 1 \
    --outFilterMismatchNmax 999 --outFilterMismatchNoverLmax 0.04 \
    --alignIntronMin 20 --alignIntronMax 1000000
done

multiqc 03_star/bam -o 07_multiqc/star

# -----------------------
# 5) StringTie (optional transcript assembly/quant)
# -----------------------
mkdir -p 05_stringtie
for BAM in 03_star/bam/*.bam; do
  SAMPLE=$(basename "${BAM}" .bam)
  stringtie "${BAM}" -p "${THREADS}" -G "${ANNOT_GTF}" -o 05_stringtie/"${SAMPLE}".gtf
done

ls 05_stringtie/*.gtf > 05_stringtie/mergelist.txt
stringtie --merge -p "${THREADS}" -G "${ANNOT_GTF}" \
  -o 05_stringtie/merged.gtf 05_stringtie/mergelist.txt

# -----------------------
# 6) TransDecoder (ORFs from merged cestode transcripts)
# -----------------------
mkdir -p 06_transdecoder
gffread 05_stringtie/merged.gtf -g "${GENOME_FA}" -w 06_transdecoder/merged_transcripts.fasta

TransDecoder.LongOrfs -t 06_transdecoder/merged_transcripts.fasta -m 3
TransDecoder.Predict  -t 06_transdecoder/merged_transcripts.fasta

# -----------------------
# 7) OrthoFinder (proteomes)
# Put here:
#  - cestode proteome(s) from UniProt (or reference annotation)
#  - Cestode proteome(s)
#  - TransDecoder predicted proteins (from 06_transdecoder)

#Retrieve the cestode proteins from the cestode genome
gffread JAUOZQ01.1.annotation_BRAKER3-v1.0.gtf \
  -g JAUOZQ01.1.fasta \
  -y JAUOZQ01.1.proteins.faa

#Run Orthofinder as previously described from Uniprot and located in proteomes_cestode.
orthofinder -f proteomes_cestode -t 12

# -----------------------
# 8) PANZZER (proteomes_cestode). REMEMEBER the "proteomes" folder contains all the proteomes downloaded from Uniprot, the A.brevis one and the etrieved from Transdecoder in order to get the GO associated.
# -----------------------
cd /c/Users/Startklar/Downloads/proteomes_cestode

# ---- run PANNZER annotation ----
python runsanspanz.py \
  -R \
  --PANZ_FILTER_PERMISSIVE \
  -m Pannzer \
  -i sans.tab \
  -f tab \
  -o ",DE.out3,GO.out3,anno.out3"
```

**Similiraty Matrix Generation**

```
#Extract the proteic sequences of the ID released in the ant´s henolymph from Hartke et al. (2023)
seqkit grep -f query_ids.txt trinity_proteins.fasta > trinity_subset.fasta

# 1) Make BLAST database from the cestode proteome
makeblastdb \
  -in JAUOZQ01.1.proteins.fasta \
  -dbtype prot \
  -out JAUOZQ01.1.proteins_db

# 2) BLASTP: Trinity/TransDecoder proteins (subset or full) vs cestode proteome
blastp \
  -query trinity_subset.fasta \
  -db JAUOZQ01.1.proteins_db \
  -evalue 1e-5 \
  -max_target_seqs 1 \
  -num_threads 12 \
  -outfmt "6 qseqid sseqid pident length qcovhsp evalue bitscore" \
  > best_hits_trinity_vs_cestode.tsv
  
#Retrieve the best hit name and the protein sequences
cut -f2 best_hits_trinity_vs_cestode.tsv | sort -u > cestode_protein_list.txt
seqkit grep -f cestode_protein_list.txt \
  JAUOZQ01.1.proteins.faa \
  > cestode_besthit_proteins.fasta
##Add these sequences to the sequences of the ant´s neuropeptides to create the Everything.fasta

#get the proteins name from the fasta
grep "^>" Everything.fasta | sed 's/^>//; s/\..*//' > list_gene.txt

#########################################################################################################################################################
FASTA="Everything.fasta"
LIST="list_gene.txt"   
SIM_CMD="/mnt/c/Users/Startklar/Desktop/fasta_tools/bin/fasta_similarity"
MAFFT_OPTS="--auto --anysymbol --quiet --thread 10"

# normalize the list (remove > and spaces, but don´t touch the text to maintain the substring)
sed -i 's/\r$//' "$LIST"
sed -i 's/^[[:space:]]*>//' "$LIST"
sed -i 's/^[[:space:]]*//; s/[[:space:]]*$//' "$LIST"

echo -e "gene1\tgene2\tsimilarity" > similarity.tsv
mapfile -t IDS < "$LIST"

extract_two_substr() {
  local q1="$1" q2="$2" outfa="$3"
  awk -v q1="$q1" -v q2="$q2" '
    BEGIN{ RS=">"; ORS="" }
    NR>1{
      full=$0                   # header + sequenza (senza il > iniziale)
      hdr=$1; sub(/\r$/,"",hdr) # primo token
      # normalizza anche un "core" senza suffissi comuni
      split(hdr,a,/[ \t\r\n]/); h=a[1]
      base=h; sub(/[:|].*/,"",base)

      keep =  index(full,q1)>0 || index(full,q2)>0 || h==q1 || h==q2 || base==q1 || base==q2
      if (keep) print ">"full
      # duplica per self-pair se il primo match corrisponde
      if (q1==q2 && (index(full,q1)>0 || h==q1 || base==q1)) print ">"full
    }' "$FASTA" > "$outfa"
}

for ((i=0;i<${#IDS[@]};i++)); do
  for ((j=0;j<${#IDS[@]};j++)); do
    g1="${IDS[i]}"; g2="${IDS[j]}"
    tmpfa=$(mktemp); tmpaln=$(mktemp)

    extract_two_substr "$g1" "$g2" "$tmpfa"
    nrec=$(grep -c '^>' "$tmpfa" || true)
    if [[ "$nrec" -ne 2 ]]; then
      echo -e "${g1}\t${g2}\tMISSING_SEQ" >> similarity.tsv
      rm -f "$tmpfa" "$tmpaln"; continue
    fi

    if ! mafft $MAFFT_OPTS "$tmpfa" > "$tmpaln" 2>/dev/null; then
      echo -e "${g1}\t${g2}\tMAFFT_FAIL" >> similarity.tsv
      rm -f "$tmpfa" "$tmpaln"; continue
    fi

    sim=$("$SIM_CMD" "$tmpaln" 2>/dev/null | grep -Eo '[-+]?[0-9]*\.?[0-9]+' | tail -n1 || true)
    [[ -z "$sim" ]] && sim="NA"
    echo -e "${g1}\t${g2}\t${sim}" >> similarity.tsv

    rm -f "$tmpfa" "$tmpaln"
  done
done

echo "Done: similarity.tsv"
```

Conitnue the analysis in R

**Load the required Llibraries**

```
library(DESeq2)
library(ggplot2)
library(dplyr)
library(tidyr)
library(tidyverse)
library(ggrastr)
library(ggrepel)
library(ggsignif)
library(patchwork)
library(data.table)
library(readxl)
library(GenomicFeatures)
library(GenomicRanges)
library(goseq)
library(GO.db)
library(AnnotationDbi)
library(stringr)
library(forcats)
library(patchwork)
library(clusterProfiler)
library(svglite)
library(gridExtra)
library(grid)
library(pheatmap)
```

**Differentially Expressed Genes**

```
#The tables are seseparated since the analysis was perfomed in 2 steps, it is sufficient to delete the first columns and continue wuth the "colnames" command
fatbody_counts <- read.table("C:/Users/Startklar/Downloads/sample.counts_afterCorrection (1).txt", header=TRUE, row.names=1, comment.char="#")
brain_counts <- read.table("C:/Users/Startklar/Downloads/sample.counts_CDSBrain (1).txt", header=TRUE, row.names=1, comment.char="#")
fatbody_counts <- fatbody_counts[,6:ncol(fatbody_counts)]
brain_counts <- brain_counts[,6:ncol(brain_counts)]
merged_counts <- merge(fatbody_counts, brain_counts, by="row.names", all=TRUE)
colnames(merged_counts)[1] <- "GeneID"
rownames(merged_counts) <- merged_counts$GeneID
merged_counts <- merged_counts[,-1]

colnames(merged_counts) <- c("MD1InfFB", "MD1NotFB","MD2InfFB", "MD2NotFB", 
                       "MD3InfFB", "MD3NotFB","MD4InfFB", "MD4NotFB", 
                        "MD5InfFB", "MD5NotFB", "Q12_FB", "Q13_FB", 
                        "Q14_FB", "Q44_FB","Q46_FB", "Q5_FB", 
                        "Q9_FB", "S14InfFB", "S14NotFB", "S5InfFB", 
                        "S5NotFB", "MD1InfBr", "MD1NotBr","MD2InfBr", "MD2NotBr", 
                        "MD3InfBr", "MD3NotBr","MD4InfBr", "MD4NotBr", 
                        "MD5InfBr", "MD5NotBr", "Q12_Br", "Q13_Br", 
                        "Q14_Br", "Q44_Br","Q46_Br", "Q5_Br", 
                        "Q9_Br", "S14InfBr", "S14NotBr", "S5InfBr", 
                        "S5NotBr")

df2<-merged_counts
##############################################################################################
#Second All the comparisons for the Fat Body
m_FB <- df2 %>%
  dplyr::select(ends_with("FB"))

m_FB <- m_FB[rowSums(m_FB >9)>=6,]

# Load sample metadata
coldata <- read.csv2("C:/Users/Startklar/Downloads/Info_data.csv", sep=",", header=TRUE, row.names = 1)
coldata$Sample_ID <- rownames(coldata)
coldataFB <- coldata[grepl("FB", coldata$Sample_ID), ]
coldataFB$Treatment <- factor(coldataFB$Treatment) 
coldataFB$ColonyID <- factor(coldataFB$ColonyID) 

all(rownames(coldataFB) %in% colnames(m_FB))
m_FB <- m_FB[, rownames(coldataFB)]
all(rownames(coldataFB) == colnames(m_FB))

dds <- DESeqDataSetFromMatrix(countData = m_FB,
                              colData = coldataFB,
                              design = ~ Treatment)


dds_FB <- DESeq(dds)
res_FB <- results(dds_FB)

#Create the normalized gene count table for later on analysis
normalized_counts_FB <- counts(dds_FB, normalize=TRUE)
normalized_counts_FB<-as.data.frame(normalized_counts_FB)
normalized_counts_FB$GeneID<-row.names(normalized_counts_FB)

#Create the PCA plot
#Transformed to rlog the data
rld <- rlog(dds)
# PCA loadings (PC1 and PC2) for supplementary table
pca_obj <- prcomp(t(assay(rld)), center = TRUE, scale. = FALSE)

pca_loadings_FB <- as.data.frame(pca_obj$rotation)
pca_loadings_FB$GeneID <- rownames(pca_loadings_FB)

pca_loadings_FB_PC12 <- pca_loadings_FB %>%
  dplyr::select(GeneID, PC1, PC2)

write.csv(
  pca_loadings_FB_PC12,
  "C:/Users/Startklar/Downloads/PCA_loadings_PC1_PC2_FatBody.csv",
  row.names = FALSE
)
#head( assay(rld) )
par( mfrow = c( 1, 2))
dds2 <- estimateSizeFactors(dds)
#plot( log2( 1+counts(dds, normalized=TRUE)[, 1:2]), col="#00000020", pch=20, cex=0.3 )
#plot( assay(rld)[, 1:2], col="#00000020", pch=20, cex=0.3 )

pcaData <- plotPCA(rld, intgroup=c("Treatment", "ColonyID"), returnData=TRUE, ntop=length(rld)) 
percentVar <- round(100 * attr(pcaData, "percentVar"))

#PCA and clustering should be done on normalized and preferably transformed read counts (rld= rlog), so that the high variability of low read counts does not occlude potentially informative data trends
colors <- c(
  "Queen" = "#166B6B",      
  "Uninfected" = "#7A7A7A",      
  "Infected" = "#F0E68C"
)

# PCA plot
plot2 <- ggplot(pcaData, aes(PC1, PC2, color=Treatment, label=name)) +
  geom_point(size = 3, stroke = 1) +
 # geom_text_repel(size = 4, box.padding = 0.6, point.padding = 2, max.overlaps = Inf) + 
  xlab(paste0("PC1: ", percentVar[1], "% variance")) +
  ylab(paste0("PC2: ", percentVar[2], "% variance")) +
  scale_color_manual(values = colors, labels= c("Uninfected"= "Uninfected Workers", "Infected" = "Infected Workers", "Queen" = "Uninfected Queens")) +
  theme(
    panel.grid.major = element_blank(), 
    panel.grid.minor = element_blank(),
    panel.background = element_blank(), 
    axis.line = element_line(colour = "black"), 
    axis.text.y = element_text(size = 18),
    axis.text.x = element_text(size = 18, color = "black"), 
    axis.title.x = element_text(size = 18), 
    axis.title.y = element_text(size = 18), 
    legend.position = "right", 
    legend.text = element_text(size = 18, color = "black"), 
    legend.title = element_text(size = 18, face = "bold")
  ) +
  coord_fixed()


# Define fill colors for ellipses
ellipse_fill_colors <- c(
  "Queen" = "#166B6B",      
  "Uninfected" = "#7A7A7A",      
  "Infected" = "#F0E68C"
)

plot2_ellipse_FB <- plot2 +
  stat_ellipse(aes(color=Treatment, fill=Treatment), alpha=0.2, geom="polygon") +  
  scale_fill_manual(values = colors, guide = "none")

ggsave("C:/Users/Startklar/Downloads/PCA_Fat_Body_old_annotation.pdf", plot2_ellipse_FB, width = 9, height = 9, dpi=300)

##############################################################################################
#Third All the comparisons for the Brain
m_Br <- df2 %>%
  dplyr::select(ends_with("Br"))

m_Br <- m_Br[rowSums(m_Br >9)>=6,]

coldata$Sample_ID <- rownames(coldata) 
coldataBr <- coldata[grepl("Br", coldata$Sample_ID), ]
coldataBr$Treatment <- factor(coldataBr$Treatment) 
coldataBr$ColonyID<- factor(coldataBr$ColonyID) 

all(rownames(coldataBr) %in% colnames(m_Br))
m_Br <- m_Br[, rownames(coldataBr)]
all(rownames(coldataBr) == colnames(m_Br))

dds <- DESeqDataSetFromMatrix(countData = m_Br,
                              colData = coldataBr,
                              design = ~ Treatment)
dds_Br <- DESeq(dds)
res_Br <- results(dds_Br)

#Create the normalized gene count table for later on analysis
normalized_counts_Br <- counts(dds_Br, normalize=TRUE)
normalized_counts_Br<-as.data.frame(normalized_counts_Br)
normalized_counts_Br$GeneID<-row.names(normalized_counts_Br)

#Create the PCA plot
#Transformed to rlog the data
rld <- rlog(dds)
# PCA loadings (PC1 and PC2) for supplementary table
pca_obj <- prcomp(t(assay(rld)), center = TRUE, scale. = FALSE)

pca_loadings_Br <- as.data.frame(pca_obj$rotation)
pca_loadings_Br$GeneID <- rownames(pca_loadings_Br)

pca_loadings_Br_PC12 <- pca_loadings_Br %>%
  dplyr::select(GeneID, PC1, PC2)

write.csv(
  pca_loadings_Br_PC12,
  "C:/Users/Startklar/Downloads/PCA_loadings_PC1_PC2_Brain.csv",
  row.names = FALSE
)
#head( assay(rld) )
par( mfrow = c( 1, 2))
#plot( log2( 1+counts(dds, normalized=TRUE)[, 1:2]), col="#00000020", pch=20, cex=0.3 )
#plot( assay(rld)[, 1:2], col="#00000020", pch=20, cex=0.3 )

pcaData <- plotPCA(rld, intgroup=c("Treatment", "ColonyID"), returnData=TRUE, ntop=length(rld)) 
percentVar <- round(100 * attr(pcaData, "percentVar"))

#PCA and clustering should be done on normalized and preferably transformed read counts (rld= rlog), so that the high variability of low read counts does not occlude potentially
colors <- c(
  "Queen" = "#166B6B",      
  "Uninfected" = "#7A7A7A",      
  "Infected" = "#F0E68C"
)

# PCA plot
plot2 <- ggplot(pcaData, aes(PC1, PC2, color=Treatment, label=name)) +
  geom_point(size = 3, stroke = 1) +
 # geom_text_repel(size = 4, box.padding = 0.6, point.padding = 2, max.overlaps = Inf) + 
  xlab(paste0("PC1: ", percentVar[1], "% variance")) +
  ylab(paste0("PC2: ", percentVar[2], "% variance")) +
  scale_color_manual(values = colors, labels= c("Uninfected"= "Uninfected Workers", "Infected" = "Infected Workers", "Queen" = "Uninfected Queens")) +
  theme(
    panel.grid.major = element_blank(), 
    panel.grid.minor = element_blank(),
    panel.background = element_blank(), 
    axis.line = element_line(colour = "black"), 
    axis.text.y = element_text(size = 18),
    axis.text.x = element_text(size = 18, color = "black"), 
    axis.title.x = element_text(size = 18), 
    axis.title.y = element_text(size = 18), 
    legend.position = "right", 
    legend.text = element_text(size = 18, color = "black"), 
    legend.title = element_text(size = 18, face = "bold")
  ) +
  coord_fixed()


# Define fill colors for ellipses
ellipse_fill_colors <- c(
  "Queen" = "#166B6B",      
  "Uninfected" = "#7A7A7A",      
  "Infected" = "#F0E68C"
)

plot2_ellipse_Br <- plot2 +
  stat_ellipse(aes(color=Treatment, fill=Treatment), alpha=0.2, geom="polygon") +  
  scale_fill_manual(values = colors, guide = "none")


ggsave("C:/Users/Startklar/Downloads/PCA_Brain.pdf", plot2_ellipse_Br, width = 9, height = 9, dpi=300)

###############################################################################################
##Extract the top 200 genes contributing to the PC! for brain and fat body in order to retrieve the main biological functions##
genes_PC1_FB <- pca_loadings_FB %>%
  dplyr::arrange(desc(abs(PC1))) %>%
  dplyr::slice(1:500) %>%
  dplyr::pull(GeneID)

genes_PC1_Br <- pca_loadings_Br %>%
  dplyr::arrange(desc(abs(PC1))) %>%
  dplyr::slice(1:500) %>%
  dplyr::pull(GeneID)
```

**Correlation Plots (Supplementary Figures)**

```
# ============================================================
# Correlation scatter: Brain vs Fat body (median log2 expression) for Neuropeptides
#
# REQUIRED OBJECTS (must already exist in your environment):
#   normalized_counts_Br : data.frame of normalized counts for BRAIN
#   normalized_counts_FB : data.frame of normalized counts for FAT BODY
#   (Each must contain a column "GeneID" OR have rownames = GeneID)
#
# ============================================================
# -------------------------------
# 0) USER INPUTS
# -------------------------------
list_path <- "C:/Users/Startklar/Desktop/Neuropeptide_Paper/List_for_R.csv"
out_pdf   <- "correlation_neuropeptides_brain_vs_fatbody.pdf"  # set NULL to not save
save_pdf  <- TRUE
pdf_w     <- 10
pdf_h     <- 6

# -------------------------------
# 1) Load candidate list and keep ONLY neuropeptides
#    Assumptions about columns in List_for_R.csv:
#      V2 = GeneID
#      V3 = Type (contains 'Neuropeptide' or 'Receptor')
#      V4 = Abbreviation (label to show on plot)
# -------------------------------
candidates <- read.csv(list_path, header = FALSE, stringsAsFactors = FALSE)

neuro_map <- candidates %>%
  filter(grepl("neuropeptide", V3, ignore.case = TRUE)) %>%
  transmute(GeneID = V2, Abbrev = V4) %>%
  distinct()

# -------------------------------
# 2) Filter counts to ONLY neuropeptides present in neuro_map
# -------------------------------
normalized_counts_Br_np <- normalized_counts_Br %>% filter(GeneID %in% neuro_map$GeneID)
normalized_counts_FB_np <- normalized_counts_FB %>% filter(GeneID %in% neuro_map$GeneID)

# -------------------------------
# 3) Build expr_long (GeneID, Sample, CountNorm, Tissue)
# -------------------------------
expr_long <- bind_rows(
  normalized_counts_Br_np %>%
    pivot_longer(cols = -GeneID, names_to = "Sample", values_to = "CountNorm") %>%
    mutate(Tissue = "Brain"),
  normalized_counts_FB_np %>%
    pivot_longer(cols = -GeneID, names_to = "Sample", values_to = "CountNorm") %>%
    mutate(Tissue = "Fat body")
)

# -------------------------------
# 4) Compute median log2 expression per GeneID × Tissue
# -------------------------------
med <- expr_long %>%
  mutate(log2expr = log2(CountNorm + 1)) %>%
  group_by(GeneID, Tissue) %>%
  summarise(median_log2 = median(log2expr, na.rm = TRUE), .groups = "drop") %>%
  filter(Tissue %in% c("Brain", "Fat body")) %>%
  pivot_wider(names_from = Tissue, values_from = median_log2)

# Attach abbreviations (labels)
med <- med %>%
  left_join(neuro_map, by = "GeneID")

# Drop any rows missing one tissue median
med <- med %>%
  filter(!is.na(Brain) & !is.na(`Fat body`)) %>%
  mutate(Difference = abs(Brain - `Fat body`))

# -------------------------------
# 5) Linear model stats (Fat body ~ Brain)
# -------------------------------
fit <- lm(`Fat body` ~ Brain, data = med)
slope <- unname(coef(fit)[2])
r2 <- summary(fit)$r.squared
pval <- summary(fit)$coefficients[2, 4]

subtitle_txt <- sprintf(
  "lm: Fat body ~ Brain    slope=%.3f   R²=%.3f   p=%.3g",
  slope, r2, pval
)

# -------------------------------
# 6) Plot (BLUE color scale)
# -------------------------------
p <- ggplot(med, aes(x = Brain, y = `Fat body`)) +
  geom_smooth(method = "lm", se = TRUE, linetype = "dashed") +
  geom_point(aes(color = Difference), size = 3, alpha = 0.9) +
  ggrepel::geom_text_repel(aes(label = Abbrev), size = 3, max.overlaps = Inf) +
  scale_color_gradient(
    name = "Difference",
    low  = "#deebf7",
    high = "#08519c"
  ) +
  labs(
    title = "Neuropeptides — median log2 expression",
    subtitle = subtitle_txt,
    x = "median Log2 Expression (Brain)",
    y = "median Log2 Expression (Fat body)"
  ) +
  theme_classic(base_size = 12)

# -------------------------------
if (isTRUE(save_pdf) && !is.null(out_pdf)) {
  ggsave(out_pdf, plot = p, width = pdf_w, height = pdf_h)
}

# ============================================================
# Correlation scatter: Brain vs Fat body (median log2 expression) for RECEPTORS
# ============================================================

# -------------------------------
# 0) USER INPUTS
# -------------------------------
list_path <- "C:/Users/Startklar/Desktop/Neuropeptide_Paper/List_for_R.csv"
out_pdf   <- "correlation_receptors_brain_vs_fatbody.pdf"  # set NULL to not save
save_pdf  <- TRUE
pdf_w     <- 10
pdf_h     <- 6

# -------------------------------
# 1) Load candidate list and keep ONLY RECEPTORS
#    Assumptions about columns in List_for_R.csv:
#      V2 = GeneID
#      V3 = Type (contains 'Neuropeptide' or 'Receptor')
#      V4 = Abbreviation (label to show on plot)
# -------------------------------
candidates <- read.csv(list_path, header = FALSE, stringsAsFactors = FALSE)

receptor_map <- candidates %>%
  filter(grepl("Receptor", V3, ignore.case = TRUE)) %>%
  transmute(GeneID = V2, Abbrev = V4) %>%
  distinct()

# -------------------------------
# 2) Filter counts to ONLY receptors present in receptor_map
# -------------------------------
normalized_counts_Br_rec <- normalized_counts_Br %>% filter(GeneID %in% receptor_map$GeneID)
normalized_counts_FB_rec <- normalized_counts_FB %>% filter(GeneID %in% receptor_map$GeneID)

# -------------------------------
# 3) Build expr_long (GeneID, Sample, CountNorm, Tissue)
# -------------------------------
expr_long <- bind_rows(
  normalized_counts_Br_rec %>%
    pivot_longer(cols = -GeneID, names_to = "Sample", values_to = "CountNorm") %>%
    mutate(Tissue = "Brain"),
  normalized_counts_FB_rec %>%
    pivot_longer(cols = -GeneID, names_to = "Sample", values_to = "CountNorm") %>%
    mutate(Tissue = "Fat body")
)

# -------------------------------
# 4) Compute median log2 expression per GeneID × Tissue
# -------------------------------
med <- expr_long %>%
  mutate(log2expr = log2(CountNorm + 1)) %>%
  group_by(GeneID, Tissue) %>%
  summarise(median_log2 = median(log2expr, na.rm = TRUE), .groups = "drop") %>%
  filter(Tissue %in% c("Brain", "Fat body")) %>%
  pivot_wider(names_from = Tissue, values_from = median_log2)

# Attach abbreviations (labels)
med <- med %>%
  left_join(receptor_map, by = "GeneID")

# Fallback to GeneID if abbreviation missing
med <- med %>%
  mutate(Label = ifelse(is.na(Abbrev) | Abbrev == "", GeneID, Abbrev))

# Drop any rows missing one tissue median
med <- med %>%
  filter(!is.na(Brain) & !is.na(`Fat body`)) %>%
  mutate(Difference = abs(Brain - `Fat body`))

# -------------------------------
# 5) Linear model stats (Fat body ~ Brain)
# -------------------------------
fit <- lm(`Fat body` ~ Brain, data = med)
slope <- unname(coef(fit)[2])
r2 <- summary(fit)$r.squared
pval <- summary(fit)$coefficients[2, 4]

subtitle_txt <- sprintf(
  "lm: Fat body ~ Brain    slope=%.3f   R²=%.3f   p=%.3g",
  slope, r2, pval
)

# -------------------------------
# 6) Plot (BLUE color scale)
# -------------------------------
p2 <- ggplot(med, aes(x = Brain, y = `Fat body`)) +
  geom_smooth(method = "lm", se = TRUE, linetype = "dashed") +
  geom_point(aes(color = Difference), size = 3, alpha = 0.9) +
  ggrepel::geom_text_repel(aes(label = Abbrev), size = 3, max.overlaps = Inf) +
  scale_color_gradient(
    name = "Difference",
    low  = "#deebf7",
    high = "#08519c"
  ) +
  labs(
    title = "Neuropeptide receptors — median log2 expression",
    subtitle = subtitle_txt,
    x = "median Log2 Expression (Brain)",
    y = "median Log2 Expression (Fat body)"
  ) +
  theme_classic(base_size = 12)

# -------------------------------
# 7) Save (optional)
# -------------------------------
if (isTRUE(save_pdf) && !is.null(out_pdf)) {
  ggsave(out_pdf, plot = p2, width = pdf_w, height = pdf_h)
}
```

**Volcano plots**

```
# ============================================================
# VOLCANO PLOTS (one per contrast) with:
# - x = log2FoldChange
# - y = -log10(padj)
# ============================================================

# --------------------------
# 1) DESeq2 results list
# --------------------------
res_list <- list(
  Queen_vs_Uninfected_FB    = results(dds_FB, contrast = c("Treatment", "Uninfected", "Queen")),
  Queen_vs_Uninfected_Br    = results(dds_Br, contrast = c("Treatment", "Uninfected", "Queen")),
  Queen_vs_Infected_FB      = results(dds_FB, contrast = c("Treatment", "Infected", "Queen")),
  Queen_vs_Infected_Br      = results(dds_Br, contrast = c("Treatment", "Infected", "Queen")),
  Uninfected_vs_Infected_FB = results(dds_FB, contrast = c("Treatment", "Infected", "Uninfected")),
  Uninfected_vs_Infected_Br = results(dds_Br, contrast = c("Treatment", "Infected", "Uninfected"))
)

alpha <- 0.05

# --------------------------
# 2) Annotation table
# --------------------------
annot <- read.csv(
  "C:/Users/Startklar/Desktop/Neuropeptide_Paper/List_for_R.csv", #Csv manually created with the annotated receptors and neuropeptides
  header = FALSE, stringsAsFactors = FALSE
) %>%
  setNames(c("Neuropeptide","GeneID","Category","Acronymus")) %>%
  mutate(GeneID = sub("_i[0-9]+$", "", GeneID))

# --------------------------
# 3) Colors (Category only)
#    (edit if your Category strings differ)
# --------------------------
cat_cols <- c(
  "Neuropeptide" = "#166B6B",
  "Receptor"     = "#F0A202"
)

# --------------------------
# 4) Build one combined DF to set global axis limits
# --------------------------
all_volc <- bind_rows(lapply(names(res_list), function(nm){
  df <- as.data.frame(res_list[[nm]])
  df$GeneID <- sub("_i[0-9]+$", "", rownames(df))
  df$contrast <- nm
  df
})) %>%
  left_join(annot, by = "GeneID") %>%
  filter(!is.na(padj)) %>%
  mutate(
    neglog10padj = -log10(padj),
    sig = padj < alpha
  )

xlims <- range(all_volc$log2FoldChange, na.rm = TRUE)
xpad  <- diff(xlims) * 0.05
xlims <- xlims + c(-xpad, xpad)

ylims <- range(all_volc$neglog10padj, na.rm = TRUE)
ypad  <- diff(ylims) * 0.05
ylims <- ylims + c(0, ypad)  # only pad upwards

# --------------------------
# 5) Loop & plot
# --------------------------
for(nm in names(res_list)){

  res_v <- as.data.frame(res_list[[nm]])
  res_v$GeneID <- sub("_i[0-9]+$", "", rownames(res_v))

  res_v <- res_v %>%
    left_join(annot, by = "GeneID") %>%
    filter(!is.na(padj)) %>%
    mutate(
      neglog10padj = -log10(padj),
      Significance = case_when(
        padj < alpha & log2FoldChange > 0 ~ "Up",
        padj < alpha & log2FoldChange < 0 ~ "Down",
        TRUE                              ~ "NS"
      )
    )

  # background points (all genes)
  # highlighted points: significant + annotated
  sig_annot <- res_v %>%
    filter(padj < alpha, !is.na(Category), !is.na(Acronymus))

  p <- ggplot(res_v, aes(x = log2FoldChange, y = neglog10padj)) +
    geom_vline(xintercept = 0, linetype = "dashed", colour = "grey60") +
    geom_hline(yintercept = -log10(alpha), linetype = "dashed", colour = "grey60") +

    # rasterized background (grey)
    geom_point_rast(color = "grey70", alpha = 0.35, size = 1.6) +

    # highlighted annotated points (color by Category: neuropeptide vs receptor)
    geom_point(
      data = sig_annot,
      aes(color = Category),
      size = 3
    ) +
    scale_color_manual(values = cat_cols, drop = FALSE) +

    # labels
    geom_label_repel(
      data = sig_annot,
      aes(label = Acronymus, color = Category),
      size = 3,
      fill = "white",
      label.size = 0.25,
      label.padding = unit(0.25, "lines"),
      box.padding = unit(0.35, "lines"),
      point.padding = unit(0.25, "lines"),
      max.overlaps = Inf,
      show.legend = FALSE
    ) +

    coord_cartesian(xlim = xlims, ylim = ylims) +
    labs(
      x = "log2 Fold Change",
      y = expression(-log[10]~"(padj)"),
      title = paste0("Volcano: ", nm),
      color = "Type"
    ) +
    theme_minimal(base_size = 16)

  ggsave(paste0(nm, "_VOLCANO.pdf"), p, width = 6.5, height = 6.5, dpi = 300)
}

##The outputs were later modified in Inkscape##
```

**Cluster Analysis**

```
library(DEGreport)
library(dplyr)
library(ggplot2)
library(ggrepel)
library(patchwork)

# -----------------------------
# Settings
# -----------------------------
treat_order <- c("Uninfected", "Infected", "Queen")

colors <- c(
  "Queen"      = "#166B6B",
  "Uninfected" = "#7A7A7A",
  "Infected"   = "#F0E68C"
)

# -----------------------------
# Load candidate list
# V2 = gene ID
# V4 = acronym / label to show
# -----------------------------
candidates <- read.csv(
  "C:/Users/Startklar/Desktop/Neuropeptide_Paper/List_for_R.csv",
  header = FALSE,
  stringsAsFactors = FALSE
)

# neuropeptides
neuro_tbl <- candidates %>%
  dplyr::filter(grepl("neuropeptide", V3, ignore.case = TRUE)) %>%
  dplyr::transmute(
    gene_id = V2,
    label   = ifelse(is.na(V4) | V4 == "", V2, V4)
  )

# receptors
recep_tbl <- candidates %>%
  dplyr::filter(grepl("receptor", V3, ignore.case = TRUE)) %>%
  dplyr::transmute(
    gene_id = V2,
    label   = ifelse(is.na(V4) | V4 == "", V2, V4)
  )

# -----------------------------
# Reusable function
# norm_counts = normalized_counts_Br or normalized_counts_FB
# dds         = dds_Br or dds_FB
# gene_tbl    = neuro_tbl or recep_tbl
# -----------------------------
make_cluster_plot <- function(norm_counts, dds, gene_tbl, panel_title = NULL) {

  # subset selected genes
  sel <- norm_counts[rownames(norm_counts) %in% gene_tbl$gene_id, , drop = FALSE]

  if (nrow(sel) == 0) {
    stop("No selected genes found in the expression matrix.")
  }

  # metadata
  meta <- as.data.frame(colData(dds))
  meta$Treatment <- factor(meta$Treatment, levels = treat_order)

  # clustering
  patt <- degPatterns(
    sel,
    metadata  = meta,
    time      = "Treatment",
    nClusters = 3,
    minc      = 3
  )

  # add labels from V4
  df_plot <- patt$normalized %>%
    dplyr::left_join(gene_tbl, by = c("genes" = "gene_id")) %>%
    dplyr::mutate(
      label = ifelse(is.na(label) | label == "", genes, label),
      Treatment = factor(Treatment, levels = treat_order)
    )

  # base plot from DEGreport
  p <- degPlotCluster(
    df_plot,
    time      = "Treatment",
    col       = "Treatment",
    lines     = FALSE,
    facet     = TRUE,
    min_genes = 3
  )

  # remove default tiny points from degPlotCluster
  p$layers <- Filter(function(l) !inherits(l$geom, "GeomPoint"), p$layers)

  # add custom layers
  p <- p +
    scale_fill_manual(values = colors) +
    scale_color_manual(values = colors) +
    geom_boxplot(
      aes(x = Treatment, y = value, fill = Treatment, color = Treatment),
      width = 0.6,
      alpha = 0.25,
      linewidth = 1.1,
      outlier.shape = NA
    ) +
    geom_point(
      aes(x = Treatment, y = value, color = Treatment),
      position = position_jitter(width = 0.12, height = 0),
      size = 2,
      alpha = 1
    ) +
    ggrepel::geom_text_repel(
      aes(x = Treatment, y = value, label = label, color = Treatment),
      size = 3,
      max.overlaps = Inf,
      box.padding = 0.25,
      point.padding = 0.15,
      segment.alpha = 0.4,
      min.segment.length = 0,
      show.legend = FALSE
    ) +
    guides(color = "none", fill = "none") +
    labs(
      title = panel_title,
      x = NULL,
      y = "Normalized expression"
    ) +
    theme_minimal(base_size = 12) +
    theme(
      legend.position = "none",
      strip.background = element_rect(fill = "grey90", color = NA),
      strip.text = element_text(size = 18, face = "bold"),
      plot.title = element_text(size = 20, face = "bold", hjust = 0.5),
      axis.title.y = element_text(size = 18, face = "bold"),
      axis.text.y  = element_text(size = 16),
      panel.grid.major.x = element_blank(),
      panel.grid.minor = element_blank(),
      axis.text.x  = element_blank(),
      axis.ticks.x = element_blank()
    )

  return(p)
}

# -----------------------------
# Build the four plots
# Assumes these objects already exist:
# normalized_counts_Br, normalized_counts_FB, dds_Br, dds_FB
# -----------------------------
p_Br_NP <- make_cluster_plot(
  norm_counts = normalized_counts_Br,
  dds         = dds_Br,
  gene_tbl    = neuro_tbl,
  panel_title = "Brain - Neuropeptides"
)

p_Br_R <- make_cluster_plot(
  norm_counts = normalized_counts_Br,
  dds         = dds_Br,
  gene_tbl    = recep_tbl,
  panel_title = "Brain - Receptors"
)

p_FB_NP <- make_cluster_plot(
  norm_counts = normalized_counts_FB,
  dds         = dds_FB,
  gene_tbl    = neuro_tbl,
  panel_title = "Fat body - Neuropeptides"
)

p_FB_R <- make_cluster_plot(
  norm_counts = normalized_counts_FB,
  dds         = dds_FB,
  gene_tbl    = recep_tbl,
  panel_title = "Fat body - Receptors"
)

# -----------------------------
# Combine plots
# -----------------------------
combined <- (p_Br_NP | p_Br_R) / (p_FB_NP | p_FB_R)

# save combined figure
ggsave(
  filename = "degPatterns_grid_labeled.svg",
  plot     = combined,
  width    = 18,
  height   = 10,
  dpi      = 300
)
```

**Venn Diagrms**

```
# One Venn per tissue (Brain, Fat body). Three sets = genes UP in Queen, Infected, Uninfected
# “UP in GROUP” = significantly higher (padj < 0.05) than BOTH other groups, using your contrasts.
library(ggVennDiagram)

# ---------- inputs ----------
# res_list already created as in your code (six DESeq2 results)

alpha <- 0.05
strip_iso <- function(x) sub("_i[0-9]+$", "", x)

sig_set <- function(res, dir = c("pos","neg"), alpha = 0.05){
  dir  <- match.arg(dir)
  df   <- as.data.frame(res)
  keep <- !is.na(df$padj) & df$padj < alpha
  if (dir == "pos") keep <- keep & df$log2FoldChange > 0
  if (dir == "neg") keep <- keep & df$log2FoldChange < 0
  strip_iso(rownames(df)[keep])
}

# ---------- Brain ----------
# Names encode contrasts you used:
# Queen_vs_Uninfected_Br: contrast = c("Treatment","Uninfected","Queen")   → pos: Uninfected > Queen
# Queen_vs_Infected_Br:   contrast = c("Treatment","Infected","Queen")     → pos: Infected   > Queen
# Uninfected_vs_Infected_Br: c("Treatment","Infected","Uninfected")        → pos: Infected   > Uninfected

Q_up_Br <- union(
  sig_set(res_list$Queen_vs_Uninfected_Br,  "neg", alpha),  # Queen > Uninfected
  sig_set(res_list$Queen_vs_Infected_Br,    "neg", alpha)   # Queen > Infected
)

I_up_Br <- union(
  sig_set(res_list$Queen_vs_Infected_Br,     "pos", alpha), # Infected > Queen
  sig_set(res_list$Uninfected_vs_Infected_Br,"pos", alpha)  # Infected > Uninfected
)

U_up_Br <- union(
  sig_set(res_list$Queen_vs_Uninfected_Br,   "pos", alpha), # Uninfected > Queen
  sig_set(res_list$Uninfected_vs_Infected_Br,"neg", alpha)  # Uninfected > Infected
)

sets_br <- list(Queen = Q_up_Br, Infected = I_up_Br, Uninfected = U_up_Br)

# ---------- Fat body ----------
Q_up_FB <- union(
  sig_set(res_list$Queen_vs_Uninfected_FB,  "neg", alpha),
  sig_set(res_list$Queen_vs_Infected_FB,    "neg", alpha)
)

I_up_FB <- union(
  sig_set(res_list$Queen_vs_Infected_FB,     "pos", alpha),
  sig_set(res_list$Uninfected_vs_Infected_FB,"pos", alpha)
)

U_up_FB <- union(
  sig_set(res_list$Queen_vs_Uninfected_FB,   "pos", alpha),
  sig_set(res_list$Uninfected_vs_Infected_FB,"neg", alpha)
)

sets_fb <- list(Queen = Q_up_FB, Infected = I_up_FB, Uninfected = U_up_FB)

#------------Statistical test---------------------------
# Brain
all_genes <- unique(unlist(sets_br))

# Overlap tables
tab_QI_br <- matrix(c(
  length(intersect(sets_br$Queen, sets_br$Infected)),
  length(setdiff(sets_br$Infected, sets_br$Queen)),
  length(setdiff(sets_br$Queen, sets_br$Infected)),
  length(setdiff(all_genes, union(sets_br$Queen, sets_br$Infected)))
), nrow = 2)

tab_QU_br <- matrix(c(
  length(intersect(sets_br$Queen, sets_br$Uninfected)),
  length(setdiff(sets_br$Uninfected, sets_br$Queen)),
  length(setdiff(sets_br$Queen, sets_br$Uninfected)),
  length(setdiff(all_genes, union(sets_br$Queen, sets_br$Uninfected)))
), nrow = 2)

# Fisher’s tests
fisher_QI_br <- fisher.test(tab_QI_br)
fisher_QU_br <- fisher.test(tab_QU_br)

fisher_QI_br
fisher_QU_br

# Fat body
all_genes_fb <- unique(unlist(sets_fb))

# Overlap tables
tab_QI_fb <- matrix(c(
  length(intersect(sets_fb$Queen, sets_fb$Infected)),
  length(setdiff(sets_fb$Infected, sets_fb$Queen)),
  length(setdiff(sets_fb$Queen, sets_fb$Infected)),
  length(setdiff(all_genes_fb, union(sets_fb$Queen, sets_fb$Infected)))
), nrow = 2)

tab_QU_fb <- matrix(c(
  length(intersect(sets_fb$Queen, sets_fb$Uninfected)),
  length(setdiff(sets_fb$Uninfected, sets_fb$Queen)),
  length(setdiff(sets_fb$Queen, sets_fb$Uninfected)),
  length(setdiff(all_genes_fb, union(sets_fb$Queen, sets_fb$Uninfected)))
), nrow = 2)

# Fisher’s tests
fisher_QI_fb <- fisher.test(tab_QI_fb)
fisher_QU_fb <- fisher.test(tab_QU_fb)

fisher_QI_fb
fisher_QU_fb

# ---------- colors ----------
pal_sets <- c(Queen = "#166B6B", Uninfected = "#7A7A7A", Infected = "#F0E68C")


p_br <- ggVennDiagram(sets_br, label_alpha = 0) +
  scale_color_manual(values = pal_sets) +
  theme_void(base_size = 12) + theme(legend.position = "none") +
  ggtitle("Brain: genes up vs both (padj < 0.05)")

p_fb <- ggVennDiagram(sets_fb, label_alpha = 0) +
  scale_color_manual(values = pal_sets) +
  theme_void(base_size = 12) + theme(legend.position = "none") +
  ggtitle("Fat body: genes up vs both (padj < 0.05)")

# ---------- save ----------

ggsave("Venn_Brain_Q_I_U.pdf", p_br, width = 6, height = 6, dpi = 300)
ggsave("Venn_FatBody_Q_I_U.pdf", p_fb, width = 6, height = 6, dpi = 300)
ggsave("Venn_Brain_FatBody_Q_I_U_side_by_side.pdf", p_br | p_fb, width = 12, height = 6, dpi = 300)

# ---------- save gene lists (write.csv only) ----------

dir.create("Venn_gene_lists", showWarnings = FALSE)

save_sets <- function(sets, tissue){

  # individual sets
  write.csv(
    data.frame(gene = sets$Queen),
    file = paste0("Venn_gene_lists/", tissue, "_UP_Queen.csv"),
    row.names = FALSE
  )

  write.csv(
    data.frame(gene = sets$Infected),
    file = paste0("Venn_gene_lists/", tissue, "_UP_Infected.csv"),
    row.names = FALSE
  )

  write.csv(
    data.frame(gene = sets$Uninfected),
    file = paste0("Venn_gene_lists/", tissue, "_UP_Uninfected.csv"),
    row.names = FALSE
  )

  # overlaps
  write.csv(
    data.frame(gene = intersect(sets$Queen, sets$Infected)),
    file = paste0("Venn_gene_lists/", tissue, "_OVERLAP_Queen_Infected.csv"),
    row.names = FALSE
  )

  write.csv(
    data.frame(gene = intersect(sets$Queen, sets$Uninfected)),
    file = paste0("Venn_gene_lists/", tissue, "_OVERLAP_Queen_Uninfected.csv"),
    row.names = FALSE
  )

  write.csv(
    data.frame(gene = intersect(sets$Infected, sets$Uninfected)),
    file = paste0("Venn_gene_lists/", tissue, "_OVERLAP_Infected_Uninfected.csv"),
    row.names = FALSE
  )

  write.csv(
    data.frame(gene = Reduce(intersect, sets)),
    file = paste0("Venn_gene_lists/", tissue, "_OVERLAP_Queen_Infected_Uninfected.csv"),
    row.names = FALSE
  )
}

# Brain
save_sets(sets_br, "Brain")

# Fat body
save_sets(sets_fb, "FatBody")


#################################################################################################Additional Reviewer Analysis##
# ---------------------------
# Missing comparison: Infected vs Uninfected
# ---------------------------

# Brain
tab_IU_br <- matrix(c(
  length(intersect(sets_br$Infected, sets_br$Uninfected)),
  length(setdiff(sets_br$Uninfected, sets_br$Infected)),
  length(setdiff(sets_br$Infected, sets_br$Uninfected)),
  length(setdiff(all_genes, union(sets_br$Infected, sets_br$Uninfected)))
), nrow = 2)

fisher_IU_br <- fisher.test(tab_IU_br)

fisher_IU_br


# Fat body
tab_IU_fb <- matrix(c(
  length(intersect(sets_fb$Infected, sets_fb$Uninfected)),
  length(setdiff(sets_fb$Uninfected, sets_fb$Infected)),
  length(setdiff(sets_fb$Infected, sets_fb$Uninfected)),
  length(setdiff(all_genes_fb, union(sets_fb$Infected, sets_fb$Uninfected)))
), nrow = 2)

fisher_IU_fb <- fisher.test(tab_IU_fb)

fisher_IU_fb
```

**Overlaps Sequence**

```
# ===========================

cd /c/Users/Startklar/Downloads || exit 1

# ---- inputs ----
PEP="GAGA-0510_final_annotation_repfilt_addreannot.pep"
DBDIR="blastdb"
DBNAME="${DBDIR}/insecta_proteomes"

# ---- 1) download Insecta RefSeq proteomes from NCBI (protein FASTA) ----
datasets download genome taxon Insecta --refseq --include protein --filename insecta_proteomes.zip
rm -rf insecta_proteomes
unzip -o insecta_proteomes.zip -d insecta_proteomes

# ---- 2) combine all protein FASTAs and create BLAST DB ----
mkdir -p "$DBDIR"
find insecta_proteomes -type f \( -name "*.faa" -o -name "*.fa" -o -name "*.fasta" -o -name "*.faa.gz" -o -name "*.fa.gz" -o -name "*.fasta.gz" \) \
  | while read -r f; do
      if [[ "$f" == *.gz ]]; then zcat "$f"; else cat "$f"; fi
    done > "${DBDIR}/insecta_proteomes.faa"

makeblastdb -in "${DBDIR}/insecta_proteomes.faa" -dbtype prot -parse_seqids -out "$DBNAME"

# ---- 3) extract peptides for Brain/FatBody overlap Queen-Infected lists (CSV -> FASTA) ----
for t in Brain FatBody; do
  cut -d, -f1 "Venn_gene_lists/${t}_OVERLAP_Queen_Infected.csv" \
    | tail -n +2 | tr -d '"' \
    | seqkit grep -n -f - "$PEP" \
    > "${t}_QI.pep.fa"
done

# ---- 4) BLASTP (evalue 1e-6) and keep best match per query (highest bitscore; tie lowest evalue) ----
for t in Brain FatBody; do
  blastp -query "${t}_QI.pep.fa" \
    -db "$DBNAME" \
    -evalue 1e-6 \
    -outfmt "6 qseqid sseqid evalue bitscore pident length stitle" \
    -num_threads 8 \
  | awk -F'\t' '{
      q=$1; e=$3+0; b=$4+0;
      if(!(q in best) || b>bb[q] || (b==bb[q] && e<be[q])){
        best[q]=$0; bb[q]=b; be[q]=e
      }
    } END{for(q in best) print best[q]}' \
  | sort -t$'\t' -k1,1 \
  > "${t}_QI.best.tsv"
done
```

**GO Enrichment**

```
## ------------------------- USER INPUTS ------------------------------------ ##
gff_path    <- "C:/Users/Startklar/Downloads/GAGA-0510_final_annotation_repfilt_addreannot.gff3"
eggnog_path <- "C:/Users/Startklar/Downloads/out.emapper.annotations_insecta.xlsx"

out_all_bp <- "GOseq_fatbody_BP_results.tsv"
out_sig_bp <- "GOseq_fatbody_BP_significant.tsv"
out_plot   <- "GOseq_fatbody_BP_top10.png"  # set NULL to skip saving

## ---------------------- 0) Required objects ------------------------------- ##
stopifnot(exists("Q_up_FB"), exists("I_up_FB"), exists("U_up_FB"), exists("sets_fb"))
stopifnot(exists("dds_FB"))  # universe must come from fat body DESeq2 dataset

## ---------------------- 1) Build DEG sets (your info) --------------------- ##
common_Q_I_fb <- intersect(Q_up_FB, I_up_FB)          # up in Queen and Infected
common_Q_U_fb <- intersect(Q_up_FB, U_up_FB)          # up in Queen and Uninfected
common_I_U_fb <- intersect(I_up_FB, U_up_FB)          # up in Infected and Uninfected
all_up_fb     <- unique(unlist(sets_fb))              # up in any caste

# Choose ONE biological question:
# (A) Convergence Q ∩ I (your previous choice)
deg_list <- unique(sub("_i\\d+$", "", common_Q_I_fb))

# (B) Any-up (uncomment if you prefer)
# deg_list <- unique(sub("_i\\d+$", "", all_up_fb))

cat("FB DEG-set sizes (isoform-level):\n")
print(lengths(list(
  Q_only = setdiff(Q_up_FB, union(I_up_FB, U_up_FB)),
  I_only = setdiff(I_up_FB, union(Q_up_FB, U_up_FB)),
  U_only = setdiff(U_up_FB, union(Q_up_FB, I_up_FB)),
  Q_I    = common_Q_I_fb,
  Q_U    = common_Q_U_fb,
  I_U    = common_I_U_fb,
  Any_up = all_up_fb
)))

## ---------------------- 2) TxDb from GFF ---------------------------------- ##
tnyl <- GenomicFeatures::makeTxDbFromGFF(
  file     = gff_path,
  format   = "gff3",
  organism = "Temnothorax nylanderi"
)

## ---------------------- 3) Build gene->GO from eggNOG Excel --------------- ##
em <- as.data.table(readxl::read_xlsx(eggnog_path))

qcol <- intersect(c("query", "#query", "Query"), names(em))[1]
if (is.na(qcol)) stop("Non trovo la colonna query (#query/query) nel file eggNOG Excel.")

gocol <- intersect(c("GOs", "GO_terms", "GO", "GO term(s)"), names(em))[1]
if (is.na(gocol)) stop("Non trovo la colonna GO (GOs/GO_terms/GO) nel file eggNOG Excel.")

em <- em[, .(q = as.character(get(qcol)), GOs = as.character(get(gocol)))]
em <- em[!is.na(GOs) & GOs != "-" & !grepl("^\\s*$", GOs)]

# collapse isoforms to gene level
em[, gene := sub("_i\\d+$", "", q)]

# split GO list (commas; sometimes semicolons)
em[, GOs := gsub(";", ",", GOs)]
em[, gene := stringr::str_extract(gene, "Tnyl_g\\d+")]
em_long <- em[, .(category = unlist(strsplit(GOs, ",", fixed = TRUE))), by = .(gene, isoform = q)]
em_long[, category := trimws(category)]
em_long <- em_long[grepl("^GO:\\d{7}$", category)]
# from "5-HT1A_Tnyl_g05209" -> "Tnyl_g05209"

# Recommended: keep ONE isoform per gene (most GO terms)
iso_go <- unique(em_long[, .(gene, isoform, category)])
go_per_iso <- iso_go[, .(GO_count = .N), by = .(gene, isoform)]
setorder(go_per_iso, gene, -GO_count, isoform)
top_iso <- go_per_iso[, .SD[1], by = gene]

gomap_df <- unique(
  iso_go[isoform %in% top_iso$isoform, .(gene, category)]
)

gomap_df[, gene := stringr::str_extract(gene, "Tnyl_g\\d+")]
gomap_df <- gomap_df[!is.na(gene)]
gomap_df <- unique(gomap_df)


# Alternative: use ALL GO per gene (comment the block above and uncomment below)
# gomap_df <- unique(em_long[, .(gene, category)])

cat("eggNOG GO mapping:\n")
cat("  genes with >=1 GO:", length(unique(gomap_df$gene)), "\n")
cat("  gene-GO pairs:", nrow(gomap_df), "\n")

## ---------------------- 4) Gene lengths (exonic) per gene ----------------- ##
exons_by_gene <- GenomicFeatures::exonsBy(tnyl, by = "gene")
gene_len <- vapply(
  exons_by_gene,
  function(gr) base::sum(GenomicRanges::width(GenomicRanges::reduce(gr))),
  numeric(1)
)

len <- data.table(gene = names(gene_len), length = as.numeric(gene_len))
len[, gene := sub("_i\\d+$", "", gene)]
len <- len[, .(length = median(length, na.rm = TRUE)), by = gene]

## ---------------------- 5) Universe = genes TESTED in fat body ------------ ##
tested_genes <- unique(sub("_i\\d+$", "", rownames(dds_FB)))

# universe must have length values (required by goseq)
universe <- sort(intersect(tested_genes, unique(len$gene)))

# keep DEG list within universe
deg_list <- intersect(deg_list, universe)

## ---------------------- 6) Align vectors for GOseq ------------------------ ##
devec <- as.integer(universe %in% deg_list)
names(devec) <- universe

len_vec <- setNames(len$length, len$gene)[universe]
len_vec[is.na(len_vec)] <- median(len_vec, na.rm = TRUE)

stopifnot(identical(names(devec), names(len_vec)))

## ---------------------- 7) Bias fit -------------------------------------- ##
pwf <- nullp(devec, bias.data = len_vec, plot.fit = FALSE)

## ---------------------- 8) Restrict GO mapping to universe ---------------- ##
gomap_use <- gomap_df[gene %in% universe]

## ---------------------- 9) GOseq (BP) ------------------------------------- ##
gores <- goseq(
  pwf,
  gene2cat                = as.data.frame(gomap_use),
  method                  = "Wallenius",
  test.cats               = "GO:BP",
  use_genes_without_cat   = TRUE
)

if (is.null(gores) || nrow(gores) == 0) stop("GOseq returned no results for fat body.")

## ---------------------- 10) Adjust + annotate ----------------------------- ##
gores$over_padj <- p.adjust(gores$over_represented_pvalue, "BH")

ann <- AnnotationDbi::select(
  GO.db, keys = gores$category,
  columns = c("TERM","ONTOLOGY"), keytype = "GOID"
)

res <- merge(gores, ann, by.x = "category", by.y = "GOID", all.x = TRUE)
res_bp <- subset(res, ONTOLOGY == "BP" & is.finite(over_padj))
res_bp <- res_bp[order(res_bp$over_padj), ]

cat(
  "FAT BODY -> Universe:", length(universe),
  " DE:", sum(devec),
  " BP tested:", nrow(res_bp),
  " BP FDR<0.05:", sum(res_bp$over_padj < 0.05), "\n"
)

write.table(res_bp, out_all_bp, sep = "\t", quote = FALSE, row.names = FALSE)

## ---------------------- 11) Significant table + DE gene lists ------------- ##
sig_fb <- subset(res_bp, over_padj < 0.05)

if (nrow(sig_fb) > 0) {
  sig_fb <- sig_fb[, c("category","TERM","numDEInCat","numInCat",
                       "over_represented_pvalue","over_padj")]
  names(sig_fb)[1:5] <- c("GO","Term","DE.in.term","Genes.in.term","P.over")

  go2genes <- split(gomap_use$gene, gomap_use$category)
  is_de <- setNames(as.logical(devec), names(devec))

  get_de <- function(goid) {
    genes <- intersect(go2genes[[goid]], names(is_de))
    paste(genes[is_de[genes]], collapse = ";")
  }
  sig_fb$DE.genes <- vapply(sig_fb$GO, get_de, character(1))

  fwrite(sig_fb, out_sig_bp, sep = "\t")
} else {
  message("No BP terms with FDR < 0.05 for fat body.")
}

## ---------------------- 12) Plot top 10 (optional) ------------------------ ##
if (nrow(sig_fb) > 0) {
  sig_fb$score <- -log10(sig_fb$over_padj)
  sig_fb$Term_clean <- forcats::fct_reorder(
    stringr::str_to_sentence(gsub("_"," ", sig_fb$Term)), sig_fb$score
  )

  p <- ggplot(sig_fb[1:min(10, nrow(sig_fb)), ],
              aes(x = Term_clean, y = score, fill = score)) +
    geom_col(color = "black", width = 0.7) +
    coord_flip() +
    labs(x = NULL, y = expression(-log[10]("FDR")),
         title = "Top enriched Biological Process terms (fat body)") +
    theme_classic(base_size = 13) +
    theme(legend.position = "none")

  print(p)
  if (!is.null(out_plot)) ggsave(out_plot, p, width = 8, height = 5, dpi = 300)
}


######################################################################################
# -------------------------- GOseq for BRAIN (eggNOG-only) -------------------
# Assumes you already built: tnyl (TxDb), len (gene lengths table),
# and gomap_df (gene->GO mapping from eggNOG Excel) as in the fat body script.

stopifnot(exists("dds_Br"))   # universe must come from brain DESeq2 dataset
stopifnot(exists("Q_up_Br"), exists("I_up_Br"), exists("U_up_Br"), exists("sets_br"))

out_all_bp_br <- "GOseq_brain_BP_results.tsv"
out_sig_bp_br <- "GOseq_brain_BP_significant.tsv"
out_plot_br   <- "GOseq_brain_BP_top10.png"  # set NULL to skip saving

## ---------------------- 1) Build DEG sets (brain) --------------------------
common_Q_I_br <- intersect(Q_up_Br, I_up_Br)
common_Q_U_br <- intersect(Q_up_Br, U_up_Br)
common_I_U_br <- intersect(I_up_Br, U_up_Br)
all_up_br     <- unique(unlist(sets_br))

# Choose ONE biological question:
# (A) Convergence Q ∩ I (consistent with your fat body choice)
deg_list_br <- unique(sub("_i\\d+$", "", common_Q_I_br))

# (B) Any-up (uncomment if you prefer)
# deg_list_br <- unique(sub("_i\\d+$", "", all_up_br))

cat("BR DEG-set sizes (isoform-level):\n")
print(lengths(list(
  Q_only = setdiff(Q_up_Br, union(I_up_Br, U_up_Br)),
  I_only = setdiff(I_up_Br, union(Q_up_Br, U_up_Br)),
  U_only = setdiff(U_up_Br, union(Q_up_Br, I_up_Br)),
  Q_I    = common_Q_I_br,
  Q_U    = common_Q_U_br,
  I_U    = common_I_U_br,
  Any_up = all_up_br
)))

## ---------------------- 2) Universe = genes TESTED in brain ----------------
tested_genes_br <- unique(sub("_i\\d+$", "", rownames(dds_Br)))
universe_br <- sort(intersect(tested_genes_br, unique(len$gene)))

# keep DEG list within universe
deg_list_br <- intersect(deg_list_br, universe_br)

## ---------------------- 3) Align vectors for GOseq -------------------------
devec_br <- as.integer(universe_br %in% deg_list_br)
names(devec_br) <- universe_br

len_vec_br <- setNames(len$length, len$gene)[universe_br]
len_vec_br[is.na(len_vec_br)] <- median(len_vec_br, na.rm = TRUE)

stopifnot(identical(names(devec_br), names(len_vec_br)))

## ---------------------- 4) Bias fit ----------------------------------------
pwf_br <- nullp(devec_br, bias.data = len_vec_br, plot.fit = FALSE)

## ---------------------- 5) Restrict GO mapping to universe -----------------
gomap_use_br <- gomap_df[gene %in% universe_br]

## ---------------------- 6) GOseq (BP) --------------------------------------
gores_br <- goseq(
  pwf_br,
  gene2cat               = as.data.frame(gomap_use_br),
  method                 = "Wallenius",
  test.cats              = "GO:BP",
  use_genes_without_cat  = TRUE
)

if (is.null(gores_br) || nrow(gores_br) == 0) stop("GOseq returned no results for brain.")

## ---------------------- 7) Adjust + annotate --------------------------------
gores_br$over_padj <- p.adjust(gores_br$over_represented_pvalue, "BH")

ann_br <- AnnotationDbi::select(
  GO.db, keys = gores_br$category,
  columns = c("TERM","ONTOLOGY"), keytype = "GOID"
)

res_br <- merge(gores_br, ann_br, by.x = "category", by.y = "GOID", all.x = TRUE)
res_bp_br <- subset(res_br, ONTOLOGY == "BP" & is.finite(over_padj))
res_bp_br <- res_bp_br[order(res_bp_br$over_padj), ]

cat(
  "BRAIN -> Universe:", length(universe_br),
  " DE:", sum(devec_br),
  " BP tested:", nrow(res_bp_br),
  " BP FDR<0.05:", sum(res_bp_br$over_padj < 0.05), "\n"
)

write.table(res_bp_br, out_all_bp_br, sep = "\t", quote = FALSE, row.names = FALSE)

## ---------------------- 8) Significant table + DE gene lists ----------------
sig_br <- subset(res_bp_br, over_padj < 0.05)

if (nrow(sig_br) > 0) {
  sig_br <- sig_br[, c("category","TERM","numDEInCat","numInCat",
                       "over_represented_pvalue","over_padj")]
  names(sig_br)[1:5] <- c("GO","Term","DE.in.term","Genes.in.term","P.over")

  go2genes_br <- split(gomap_use_br$gene, gomap_use_br$category)
  is_de_br <- setNames(as.logical(devec_br), names(devec_br))

  get_de_br <- function(goid) {
    genes <- intersect(go2genes_br[[goid]], names(is_de_br))
    paste(genes[is_de_br[genes]], collapse = ";")
  }
  sig_br$DE.genes <- vapply(sig_br$GO, get_de_br, character(1))

  fwrite(sig_br, out_sig_bp_br, sep = "\t")
} else {
  message("No BP terms with FDR < 0.05 for brain.")
}

## ---------------------- 9) Plot top 10 (optional) ---------------------------
if (nrow(sig_br) > 0) {
  sig_br$score <- -log10(sig_br$over_padj)
  sig_br$Term_clean <- forcats::fct_reorder(
    stringr::str_to_sentence(gsub("_"," ", sig_br$Term)), sig_br$score
  )

  p_br <- ggplot(sig_br[1:min(10, nrow(sig_br)), ],
                 aes(x = Term_clean, y = score, fill = score)) +
    geom_col(color = "black", width = 0.7) +
    coord_flip() +
    labs(x = NULL, y = expression(-log[10]("FDR")),
         title = "Top enriched Biological Process terms (brain)") +
    theme_classic(base_size = 13) +
    theme(legend.position = "none")

  print(p_br)
  if (!is.null(out_plot_br)) ggsave(out_plot_br, p_br, width = 8, height = 5, dpi = 300)
}
```

**GO Enrichment for PC1 Brain and Fat Body (Supplementary Materil)**

```
## ------------------------- USER INPUTS ------------------------------------ ##
gff_path    <- "C:/Users/Startklar/Downloads/GAGA-0510_final_annotation_repfilt_addreannot.gff3"
eggnog_path <- "C:/Users/Startklar/Downloads/out.emapper.annotations_insecta.xlsx"

out_all_bp    <- "GOseq_fatbody_PC1_BP_results.tsv"
out_sig_bp    <- "GOseq_fatbody_PC1_BP_significant.tsv"
out_plot      <- "GOseq_fatbody_PC1_BP_top10.png"   # set NULL to skip saving

out_all_bp_br <- "GOseq_brain_PC1_BP_results.tsv"
out_sig_bp_br <- "GOseq_brain_PC1_BP_significant.tsv"
out_plot_br   <- "GOseq_brain_PC1_BP_top10.png"     # set NULL to skip saving

## ---------------------- 0) Required objects ------------------------------- ##
stopifnot(exists("genes_PC1_FB"))
stopifnot(exists("genes_PC1_Br"))
stopifnot(exists("dds_FB"))
stopifnot(exists("dds_Br"))

## ---------------------- 1) PCA-PC1 gene lists ----------------------------- ##
deg_list <- unique(sub("_i\\d+$", "", genes_PC1_FB))
deg_list_br <- unique(sub("_i\\d+$", "", genes_PC1_Br))

cat("Fat body PCA-PC1 gene set size:", length(deg_list), "\n")
cat("Brain PCA-PC1 gene set size:", length(deg_list_br), "\n")

## ---------------------- 2) TxDb from GFF ---------------------------------- ##
tnyl <- GenomicFeatures::makeTxDbFromGFF(
  file     = gff_path,
  format   = "gff3",
  organism = "Temnothorax nylanderi"
)

## ---------------------- 3) Build gene->GO from eggNOG Excel --------------- ##
em <- as.data.table(readxl::read_xlsx(eggnog_path))

qcol <- intersect(c("query", "#query", "Query"), names(em))[1]
if (is.na(qcol)) stop("Non trovo la colonna query (#query/query) nel file eggNOG Excel.")

gocol <- intersect(c("GOs", "GO_terms", "GO", "GO term(s)"), names(em))[1]
if (is.na(gocol)) stop("Non trovo la colonna GO (GOs/GO_terms/GO) nel file eggNOG Excel.")

em <- em[, .(q = as.character(get(qcol)), GOs = as.character(get(gocol)))]
em <- em[!is.na(GOs) & GOs != "-" & !grepl("^\\s*$", GOs)]

# collapse isoforms to gene level
em[, gene := sub("_i\\d+$", "", q)]

# split GO list (commas; sometimes semicolons)
em[, GOs := gsub(";", ",", GOs)]
em[, gene := stringr::str_extract(gene, "Tnyl_g\\d+")]
em_long <- em[, .(category = unlist(strsplit(GOs, ",", fixed = TRUE))), by = .(gene, isoform = q)]
em_long[, category := trimws(category)]
em_long <- em_long[grepl("^GO:\\d{7}$", category)]

# keep ONE isoform per gene (most GO terms)
iso_go <- unique(em_long[, .(gene, isoform, category)])
go_per_iso <- iso_go[, .(GO_count = .N), by = .(gene, isoform)]
setorder(go_per_iso, gene, -GO_count, isoform)
top_iso <- go_per_iso[, .SD[1], by = gene]

gomap_df <- unique(
  iso_go[isoform %in% top_iso$isoform, .(gene, category)]
)

gomap_df[, gene := stringr::str_extract(gene, "Tnyl_g\\d+")]
gomap_df <- gomap_df[!is.na(gene)]
gomap_df <- unique(gomap_df)

cat("eggNOG GO mapping:\n")
cat("  genes with >=1 GO:", length(unique(gomap_df$gene)), "\n")
cat("  gene-GO pairs:", nrow(gomap_df), "\n")

## ---------------------- 4) Gene lengths (exonic) per gene ----------------- ##
exons_by_gene <- GenomicFeatures::exonsBy(tnyl, by = "gene")

gene_len <- sapply(seq_along(exons_by_gene), function(i) {
  sum(GenomicRanges::width(GenomicRanges::reduce(exons_by_gene[i])))
})

len <- data.table(gene = names(gene_len), length = as.numeric(gene_len))
len[, gene := sub("_i\\d+$", "", gene)]
len <- len[, .(length = median(length, na.rm = TRUE)), by = gene]

################################################################################
#                                 FAT BODY
################################################################################

## ---------------------- 5) Universe = genes TESTED in fat body ------------ ##
tested_genes <- unique(sub("_i\\d+$", "", rownames(dds_FB)))

# universe must have length values
universe <- sort(intersect(tested_genes, unique(len$gene)))

# keep PCA list within universe
deg_list <- intersect(deg_list, universe)

## ---------------------- 6) Align vectors for GOseq ------------------------ ##
devec <- as.integer(universe %in% deg_list)
names(devec) <- universe

len_vec <- setNames(len$length, len$gene)[universe]
len_vec[is.na(len_vec)] <- median(len_vec, na.rm = TRUE)

stopifnot(identical(names(devec), names(len_vec)))

## ---------------------- 7) Bias fit --------------------------------------- ##
pwf <- nullp(devec, bias.data = len_vec, plot.fit = FALSE)

## ---------------------- 8) Restrict GO mapping to universe ---------------- ##
gomap_use <- gomap_df[gene %in% universe]

## ---------------------- 9) GOseq (BP) ------------------------------------- ##
gores <- goseq(
  pwf,
  gene2cat              = as.data.frame(gomap_use),
  method                = "Wallenius",
  test.cats             = "GO:BP",
  use_genes_without_cat = TRUE
)

if (is.null(gores) || nrow(gores) == 0) stop("GOseq returned no results for fat body.")

## ---------------------- 10) Adjust + annotate ----------------------------- ##
gores$over_padj <- p.adjust(gores$over_represented_pvalue, "BH")

ann <- AnnotationDbi::select(
  GO.db,
  keys    = gores$category,
  columns = c("TERM", "ONTOLOGY"),
  keytype = "GOID"
)

res <- merge(gores, ann, by.x = "category", by.y = "GOID", all.x = TRUE)
res_bp <- subset(res, ONTOLOGY == "BP" & is.finite(over_padj))
res_bp <- res_bp[order(res_bp$over_padj), ]

cat(
  "FAT BODY -> Universe:", length(universe),
  " DE:", sum(devec),
  " BP tested:", nrow(res_bp),
  " BP FDR<0.05:", sum(res_bp$over_padj < 0.05), "\n"
)

write.table(res_bp, out_all_bp, sep = "\t", quote = FALSE, row.names = FALSE)

## ---------------------- 11) Significant table + DE gene lists ------------- ##
sig_fb <- subset(res_bp, over_padj < 0.05)

if (nrow(sig_fb) > 0) {
  sig_fb <- sig_fb[, c("category", "TERM", "numDEInCat", "numInCat",
                       "over_represented_pvalue", "over_padj")]
  names(sig_fb)[1:6] <- c("GO", "Term", "DE.in.term", "Genes.in.term", "P.over", "FDR")

  go2genes <- split(gomap_use$gene, gomap_use$category)
  is_de <- setNames(as.logical(devec), names(devec))

  get_de <- function(goid) {
    genes <- intersect(go2genes[[goid]], names(is_de))
    paste(genes[is_de[genes]], collapse = ";")
  }

  sig_fb$DE.genes <- vapply(sig_fb$GO, get_de, character(1))
  data.table::fwrite(sig_fb, out_sig_bp, sep = "\t")
} else {
  message("No BP terms with FDR < 0.05 for fat body.")
}

## ---------------------- 12) Plot top 10 (optional) ------------------------ ##
if (nrow(sig_fb) > 0) {
  sig_fb$score <- -log10(sig_fb$FDR)
  sig_fb$Term_clean <- forcats::fct_reorder(
    stringr::str_to_sentence(gsub("_", " ", sig_fb$Term)),
    sig_fb$score
  )

  p <- ggplot(sig_fb[1:min(10, nrow(sig_fb)), ],
              aes(x = Term_clean, y = score, fill = score)) +
    geom_col(color = "black", width = 0.7) +
    coord_flip() +
    labs(x = NULL, y = expression(-log[10]("FDR")),
         title = "Top enriched Biological Process terms (fat body, PC1 genes)") +
    theme_classic(base_size = 13) +
    theme(legend.position = "none")

  print(p)
  if (!is.null(out_plot)) ggsave(out_plot, p, width = 8, height = 5, dpi = 300)
}

################################################################################
#                                   BRAIN
################################################################################

## ---------------------- 13) Universe = genes TESTED in brain -------------- ##
tested_genes_br <- unique(sub("_i\\d+$", "", rownames(dds_Br)))
universe_br <- sort(intersect(tested_genes_br, unique(len$gene)))

# keep PCA list within universe
deg_list_br <- intersect(deg_list_br, universe_br)

## ---------------------- 14) Align vectors for GOseq ----------------------- ##
devec_br <- as.integer(universe_br %in% deg_list_br)
names(devec_br) <- universe_br

len_vec_br <- setNames(len$length, len$gene)[universe_br]
len_vec_br[is.na(len_vec_br)] <- median(len_vec_br, na.rm = TRUE)

stopifnot(identical(names(devec_br), names(len_vec_br)))

## ---------------------- 15) Bias fit -------------------------------------- ##
pwf_br <- nullp(devec_br, bias.data = len_vec_br, plot.fit = FALSE)

## ---------------------- 16) Restrict GO mapping to universe --------------- ##
gomap_use_br <- gomap_df[gene %in% universe_br]

## ---------------------- 17) GOseq (BP) ------------------------------------ ##
gores_br <- goseq(
  pwf_br,
  gene2cat              = as.data.frame(gomap_use_br),
  method                = "Wallenius",
  test.cats             = "GO:BP",
  use_genes_without_cat = TRUE
)

if (is.null(gores_br) || nrow(gores_br) == 0) stop("GOseq returned no results for brain.")

## ---------------------- 18) Adjust + annotate ----------------------------- ##
gores_br$over_padj <- p.adjust(gores_br$over_represented_pvalue, "BH")

ann_br <- AnnotationDbi::select(
  GO.db,
  keys    = gores_br$category,
  columns = c("TERM", "ONTOLOGY"),
  keytype = "GOID"
)

res_br <- merge(gores_br, ann_br, by.x = "category", by.y = "GOID", all.x = TRUE)
res_bp_br <- subset(res_br, ONTOLOGY == "BP" & is.finite(over_padj))
res_bp_br <- res_bp_br[order(res_bp_br$over_padj), ]

cat(
  "BRAIN -> Universe:", length(universe_br),
  " DE:", sum(devec_br),
  " BP tested:", nrow(res_bp_br),
  " BP FDR<0.05:", sum(res_bp_br$over_padj < 0.05), "\n"
)

write.table(res_bp_br, out_all_bp_br, sep = "\t", quote = FALSE, row.names = FALSE)

## ---------------------- 19) Significant table + DE gene lists ------------- ##
sig_br <- subset(res_bp_br, over_padj < 0.05)

if (nrow(sig_br) > 0) {
  sig_br <- sig_br[, c("category", "TERM", "numDEInCat", "numInCat",
                       "over_represented_pvalue", "over_padj")]
  names(sig_br)[1:6] <- c("GO", "Term", "DE.in.term", "Genes.in.term", "P.over", "FDR")

  go2genes_br <- split(gomap_use_br$gene, gomap_use_br$category)
  is_de_br <- setNames(as.logical(devec_br), names(devec_br))

  get_de_br <- function(goid) {
    genes <- intersect(go2genes_br[[goid]], names(is_de_br))
    paste(genes[is_de_br[genes]], collapse = ";")
  }

  sig_br$DE.genes <- vapply(sig_br$GO, get_de_br, character(1))
  data.table::fwrite(sig_br, out_sig_bp_br, sep = "\t")
} else {
  message("No BP terms with FDR < 0.05 for brain.")
}

## ---------------------- 20) Plot top 10 (optional) ------------------------ ##
if (nrow(sig_br) > 0) {
  sig_br$score <- -log10(sig_br$FDR)
  sig_br$Term_clean <- forcats::fct_reorder(
    stringr::str_to_sentence(gsub("_", " ", sig_br$Term)),
    sig_br$score
  )

  p_br <- ggplot(sig_br[1:min(10, nrow(sig_br)), ],
                 aes(x = Term_clean, y = score, fill = score)) +
    geom_col(color = "black", width = 0.7) +
    coord_flip() +
    labs(x = NULL, y = expression(-log[10]("FDR")),
         title = "Top enriched Biological Process terms (brain, PC1 genes)") +
    theme_classic(base_size = 13) +
    theme(legend.position = "none")

  print(p_br)
  if (!is.null(out_plot_br)) ggsave(out_plot_br, p_br, width = 8, height = 5, dpi = 300)
}
```

**Boxplot for the Venn overlaps**

```
res_list <- list(
Queen_vs_Uninfected_FB = results(dds_FB, contrast = c("Treatment", "Uninfected", "Queen")),
Queen_vs_Uninfected_Br = results(dds_Br, contrast = c("Treatment", "Uninfected", "Queen")),
Queen_vs_Infected_FB = results(dds_FB, contrast = c("Treatment", "Infected", "Queen")),
Queen_vs_Infected_Br = results(dds_Br, contrast = c("Treatment", "Infected", "Queen")),
Uninfected_vs_Infected_FB = results(dds_FB, contrast = c("Treatment", "Infected", "Uninfected")),
Uninfected_vs_Infected_Br = results(dds_Br, contrast = c("Treatment", "Infected", "Uninfected"))
)

# Normalized counts for fat body
norm_counts_FB <- counts(dds_FB, normalized = TRUE)

# Normalized counts for brain
norm_counts_Br <- counts(dds_Br, normalized = TRUE)


#genes of interest
genes_FatBody <- c("Tnyl_g02946", "Tnyl_g13343", "Tnyl_g06350", "Tnyl_g06972", "Tnyl_g03690", "Tnyl_g15396", "Tnyl_g12751", "Tnyl_g10763")
genes_Brain <- c("Tnyl_g13343", "Tnyl_g10971", "Tnyl_g06326", "Tnyl_g06326") #"Tnyl_g03511", "Tnyl_g15396")

gene_labels_brain <- c(
  "Tnyl_g13343" = "<span style='font-weight:bold; font-size:14pt;'>Telomerase reverse transcriptase</span><br>(TERT, Tnyl_g13343)",
  "Tnyl_g10971" = "<span style='font-weight:bold; font-size:14pt;'>Protein takeout-like</span><br>(TOL, Tnyl_g10971)",
  "Tnyl_g06326" = "<span style='font-weight:bold; font-size:14pt;'>Apolipoprotein D</span><br>(ApoD, Tnyl_g06326)"
  # "Tnyl_g03511" = "<span style='font-weight:bold; font-size:14pt;'>transferrin</span><br>Tnyl_g03511",
  # "Tnyl_g15396" = "<span style='font-weight:bold; font-size:14pt;'>transferrin 1</span><br>Tnyl_g15396"
)

gene_labels_fb <- c(
  "Tnyl_g13343" = "<span style='font-weight:bold; font-size:14pt;'>Telomerase reverse transcriptase</span><br>(TERT, Tnyl_g13343)",
  "Tnyl_g02946" = "<span style='font-weight:bold; font-size:14pt;'>S-adenosylmethionine sensor upstream of mTORC1</span><br>(SAMTOR, Tnyl_g02946)",
  "Tnyl_g06350" = "<span style='font-weight:bold; font-size:14pt;'>Protein takeout-like</span><br>(TOL,Tnyl_g06350)",
  "Tnyl_g06972" = "<span style='font-weight:bold; font-size:14pt;'>Peroxiredoxin-like protein</span><br>(Prx-like, Tnyl_g06972)",
  "Tnyl_g03690" = "<span style='font-weight:bold; font-size:14pt;'>Protein yellow</span><br>(Tnyl_g03690)",
  "Tnyl_g15396" = "<span style='font-weight:bold; font-size:14pt;'>Transferrin</span><br>(TF,Tnyl_g15396)",
  "Tnyl_g12751" = "<span style='font-weight:bold; font-size:14pt;'>X-ray repair cross-complementing protein 3</span><br>(XRCC3, Tnyl_g12751)",
  "Tnyl_g10763" = "<span style='font-weight:bold; font-size:14pt;'>Epidermal growth factor receptor</span><br>(EGFR, Tnyl_g10763)"
)

## --------------------------------------------------------------------
## p-value -> stars
## --------------------------------------------------------------------
p_to_stars <- function(p) {
  if (is.na(p)) return(NA_character_)
  if (p <= 0.0001) return("****")
  if (p <= 0.001) return("***")
  if (p <= 0.01)  return("**")
  if (p <= 0.05)  return("*")
  return("")  # non significativo -> nessuna etichetta
}

## --------------------------------------------------------------------
## Significance data.frame: BRAIN
## --------------------------------------------------------------------
make_sig_df_brain <- function(genes, br_long, res_list) {

  range_y <- br_long |>
    mutate(norm_pos = ifelse(norm_count > 0, norm_count, NA)) |>
    group_by(gene) |>
    summarise(
      y_min = min(norm_pos, na.rm = TRUE),
      y_max = max(norm_pos, na.rm = TRUE),
      .groups = "drop"
    ) |>
    mutate(
      y_range = ifelse(
        is.finite(y_max - y_min) & (y_max - y_min) > 0,
        y_max - y_min,
        y_max * 0.2
      )
    )

  # comp order: Q–U (1), U–I (2), Q–I (3)
  sig_list <- list(
    data.frame(
      gene   = genes,
      group1 = "Queen",
      group2 = "Uninfected",
      padj   = res_list$Queen_vs_Uninfected_Br[genes, "padj"],
      comp   = 1L
    ),
    data.frame(
      gene   = genes,
      group1 = "Uninfected",
      group2 = "Infected",
      padj   = res_list$Uninfected_vs_Infected_Br[genes, "padj"],
      comp   = 2L
    ),
    data.frame(
      gene   = genes,
      group1 = "Queen",
      group2 = "Infected",
      padj   = res_list$Queen_vs_Infected_Br[genes, "padj"],
      comp   = 3L
    )
  )

  sig_df <- bind_rows(sig_list) |>
    mutate(
      label  = vapply(padj, p_to_stars, character(1)),
      group1 = factor(group1, levels = c("Queen", "Uninfected", "Infected")),
      group2 = factor(group2, levels = c("Queen", "Uninfected", "Infected"))
    ) |>
    left_join(range_y, by = "gene") |>
    mutate(
      y_raw      = y_max + 0.10 * y_range * comp,
      y_position = ifelse(is.finite(y_raw) & y_raw > 0,
                          log10(y_raw),
                          NA_real_)
    ) |>
    # tieni solo confronti con padj < 0.05 e posizioni valide
    filter(!is.na(y_position) & padj < 0.05)

  sig_df
}

## --------------------------------------------------------------------
## Significance data.frame: FAT BODY
## --------------------------------------------------------------------
make_sig_df_fb <- function(genes, fb_long, res_list) {

  range_y <- fb_long |>
    mutate(norm_pos = ifelse(norm_count > 0, norm_count, NA)) |>
    group_by(gene) |>
    summarise(
      y_min = min(norm_pos, na.rm = TRUE),
      y_max = max(norm_pos, na.rm = TRUE),
      .groups = "drop"
    ) |>
    mutate(
      y_range = ifelse(
        is.finite(y_max - y_min) & (y_max - y_min) > 0,
        y_max - y_min,
        y_max * 0.2
      )
    )

  # comp order: Q–U (1), U–I (2), Q–I (3)
  sig_list <- list(
    data.frame(
      gene   = genes,
      group1 = "Queen",
      group2 = "Uninfected",
      padj   = res_list$Queen_vs_Uninfected_FB[genes, "padj"],
      comp   = 1L
    ),
    data.frame(
      gene   = genes,
      group1 = "Uninfected",
      group2 = "Infected",
      padj   = res_list$Uninfected_vs_Infected_FB[genes, "padj"],
      comp   = 2L
    ),
    data.frame(
      gene   = genes,
      group1 = "Queen",
      group2 = "Infected",
      padj   = res_list$Queen_vs_Infected_FB[genes, "padj"],
      comp   = 3L
    )
  )

  sig_df <- bind_rows(sig_list) |>
    mutate(
      label  = vapply(padj, p_to_stars, character(1)),
      group1 = factor(group1, levels = c("Queen", "Uninfected", "Infected")),
      group2 = factor(group2, levels = c("Queen", "Uninfected", "Infected"))
    ) |>
    left_join(range_y, by = "gene") |>
    mutate(
      y_raw      = y_max + 0.10 * y_range * comp,
      y_position = ifelse(is.finite(y_raw) & y_raw > 0,
                          log10(y_raw),
                          NA_real_)
    ) |>
    filter(!is.na(y_position) & padj < 0.05)

  sig_df
}

## --------------------------------------------------------------------
## Build sig data.frames (assumes genes_Brain, genes_FatBody, br_long,
## fb_long, res_list already exist)
## --------------------------------------------------------------------
sample_info_br <- as.data.frame(colData(dds_Br)) |>
  rownames_to_column("sample")

sample_info_fb <- as.data.frame(colData(dds_FB)) |>
  rownames_to_column("sample")

## make sure Treatment has the right order
sample_info_br$Treatment <- factor(sample_info_br$Treatment,
                                   levels = c("Queen", "Uninfected", "Infected"))
sample_info_fb$Treatment <- factor(sample_info_fb$Treatment,
                                   levels = c("Queen", "Uninfected", "Infected"))

br_long <- norm_counts_Br[genes_Brain, , drop = FALSE] |>
  as.data.frame() |>
  rownames_to_column("gene") |>
  pivot_longer(
    cols      = -gene,
    names_to  = "sample",
    values_to = "norm_count"
  ) |>
  left_join(sample_info_br, by = "sample")

fb_long <- norm_counts_FB[genes_FatBody, , drop = FALSE] |>
  as.data.frame() |>
  rownames_to_column("gene") |>
  pivot_longer(
    cols      = -gene,
    names_to  = "sample",
    values_to = "norm_count"
  ) |>
  left_join(sample_info_fb, by = "sample")


sig_brain   <- make_sig_df_brain(genes_Brain,   br_long, res_list)
sig_fatbody <- make_sig_df_fb(genes_FatBody, fb_long,  res_list)

## --------------------------------------------------------------------
## Prepare data for plotting
## --------------------------------------------------------------------
br_long$group <- factor(br_long$Treatment,
                        levels = c("Queen", "Uninfected", "Infected"))
fb_long$group <- factor(fb_long$Treatment,
                        levels = c("Queen", "Uninfected", "Infected"))

br_long_plot <- br_long |> filter(gene %in% genes_Brain)
fb_long_plot <- fb_long |> filter(gene %in% genes_FatBody)

## --------------------------------------------------------------------
## Plots (assumes gene_labels_brain, gene_labels_fb, colors, base_theme)
## --------------------------------------------------------------------
sample_info_br <- as.data.frame(colData(dds_Br)) |>
  rownames_to_column("sample")

sample_info_fb <- as.data.frame(colData(dds_FB)) |>
  rownames_to_column("sample")

## make sure Treatment has the right order
sample_info_br$Treatment <- factor(sample_info_br$Treatment,
                                   levels = c("Queen", "Uninfected", "Infected"))
sample_info_fb$Treatment <- factor(sample_info_fb$Treatment,
                                   levels = c("Queen", "Uninfected", "Infected"))

p_brain <- ggplot(br_long_plot,
                  aes(x = group, y = norm_count, fill = group)) +
  geom_boxplot(outlier.shape = NA) +
  geom_jitter(width = 0.15, alpha = 0.6) +
  facet_wrap(~ gene, scales = "free_y",
             labeller = as_labeller(gene_labels_brain)) +
  scale_fill_manual(values = colors) +
  scale_x_discrete(limits=c("Uninfected","Infected","Queen")) +
  scale_y_continuous(trans = "log10") +
  labs(title = "Brain", y = "Normalized gene counts (log₁₀)") +
  theme_classic() +
  theme(
    strip.text = ggtext::element_markdown(), legend.position="none", axis.text.x=element_blank(),
        axis.ticks.x=element_blank(), axis.title.x = element_blank(), axis.text.y   = element_text(size = 14)) +
  ggsignif::geom_signif(
    data        = sig_brain,
    aes(xmin = group1, xmax = group2,
        annotations = label, y_position = y_position),
    manual      = TRUE,
    tip_length  = 0,
    textsize    = 10,
    vjust       = 0.3,
    inherit.aes = FALSE
  )

p_fatbody <- ggplot(fb_long_plot,
                    aes(x = group, y = norm_count, fill = group)) +
  geom_boxplot(outlier.shape = NA) +
  geom_jitter(width = 0.15, alpha = 0.6) +
  facet_wrap(~ gene, scales = "free_y",
             labeller = as_labeller(gene_labels_fb)) +
  scale_fill_manual(values = colors) +
  scale_y_continuous(trans = "log10") +
  scale_x_discrete(limits=c("Uninfected","Infected","Queen")) +
  labs(title = "Fat body", y = "Normalized gene counts (log₁₀)") +
  theme_classic() +
  theme(
    strip.text = ggtext::element_markdown(), legend.position="none", axis.text.x=element_blank(),
        axis.ticks.x=element_blank(), axis.title.x = element_blank(),  axis.text.y   = element_text(size = 14)  # <- important
  ) +
  ggsignif::geom_signif(
    data        = sig_fatbody,
    aes(xmin = group1, xmax = group2,
        annotations = label, y_position = y_position),
    manual      = TRUE,
    tip_length  = 0,
    textsize    = 5,
    vjust       = 0.3,
    inherit.aes = FALSE
  )

combined_plot <- p_brain / p_fatbody +
  plot_layout(heights = c(1, 3))

combined_plot

ggsave(
  "C:/Users/Startklar/Downloads/combined_brain_fatbody_significance.pdf",
  combined_plot,
  width = 12,
  height = 15
)
```

**Orthofinder heatmaps (Supplementary Material)**

```
# ========================
# GLOBAL PLOT SETTINGS
# =========================
x_tick_size <- 20   # Orthogroups labels
y_tick_size <- 20   # Species labels
legend_text_size <- 25
legend_title_size <- 25

# =========================
# ANTS
# =========================

list_ant <- read.csv("C:/Users/Startklar/Desktop/Neuropeptide_Paper/List_for_R.csv", header = FALSE)

Ortho_ant <- read.delim(
  "C:/Users/Startklar/Desktop/Orthogroups_new_formica.tsv",
  stringsAsFactors = FALSE,
  check.names = FALSE
)

gene_col_ant <- "GAGA-0510_final_annotation_repfilt_addreannot.pep"
targets_ant  <- unique(as.character(list_ant$V2))

Ortho_hits_long_ant <- Ortho_ant %>%
  dplyr::select(Orthogroup, all_of(gene_col_ant)) %>%
  separate_rows(.data[[gene_col_ant]], sep = ",\\s*") %>%
  mutate(
    token = str_trim(.data[[gene_col_ant]]),
    gene_base = str_extract(token, "Tnyl_g\\d+")
  ) %>%
  filter(!is.na(gene_base), gene_base %in% targets_ant) %>%
  distinct(Orthogroup, gene_base, token)

Ortho_hits_ant <- Ortho_ant %>%
  semi_join(Ortho_hits_long_ant %>% distinct(Orthogroup), by = "Orthogroup")

cat("ANTS | Orthogroups found:", length(unique(Ortho_hits_ant$Orthogroup)), "\n")
cat("ANTS | Genes found:", length(unique(Ortho_hits_long_ant$gene_base)),
    "out of", length(targets_ant), "\n")
print(setdiff(targets_ant, unique(Ortho_hits_long_ant$gene_base)))

a_ant <- read.delim("C:/Users/Startklar/Desktop/Neuropeptide_Paper/Orthogroups.GeneCount_ant.tsv")
a_ant <- a_ant[, -which(names(a_ant) %in% c("Total")), drop = FALSE]

a_joined_ant <- Ortho_hits_ant %>%
  left_join(a_ant, by = c("Orthogroup"))

# keep your original column drops
a_joined_ant <- a_joined_ant[, -(2:31)]
a_joined_ant <- a_joined_ant[, -(3:4)]

# species labels (ANTS)
species_labels_ant <- c(
  "Acromyrmex_charruanus"   = "A. charruanus",
  "Acromyrmex_echinatior"   = "A. echinatior",
  "Acromyrmex_heyeri"       = "A. heyeri",
  "Acromyrmex_insinuator"   = "A. insinuator",
  "Atta_cephalotes"         = "A. cephalotes",
  "Atta_colombica"          = "A. colombica",
  "Camponotus_floridanus"   = "C. floridanus",
  "Cardiocondyla_obscurior" = "C. obscurior",
  "Cyphomyrmex_costatus"    = "C. costatus",
  "Dinoponera_quadriceps"   = "D. quadriceps",
  "Harpegnathos.saltator"   = "H. saltator",
  "Lasius_niger"            = "L. niger",
  "Lasius_platythorax"      = "L. platythorax",
  "Ooceraea_biroi"          = "O. biroi",
  "Pogonomyrmex_barbatus"   = "P. barbatus",
  "Pseudoatta_argentina"    = "P. argentina",
  "Temnothorax_curvispinosus"  = "T. curvispinosus",
  "Temnothorax_longispinosus"  = "T. longispinosus",
  "Trachymyrmex_cornetzi"      = "T. cornetzi",
  "Trachymyrmex_septentrionalis" = "T. septentrionalis",
  "Trachymyrmex_zeteki"        = "T. zeteki",
  "Apis_cerana_cerana"      = "A. cerana",
  "Apis_mellifera"          = "A. mellifera",
  "Apis_mellifera_carnica"  = "A. mellifera (carnica)",
  "Bombus_bifarius"         = "B. bifarius",
  "Bombus_impatiens"        = "B. impatiens",
  "Bombus_terrestris"       = "B. terrestris",
  "Melipona_bicolor"        = "M. bicolor",
  "Tetragonisca_angustula"  = "T. angustula",
  "Drosophila_melanogaster.y" = "D. melanogaster",
  "GAGA.0510_final_annotation_repfilt_addreannot.pep" = "T. nylanderi (annotation)",
  "merged_Br.fasta..transdecoder" = "Transdecoder Brain",
  "merged_output.old_FB.fasta..transdecoder" = "Transdecoder Fat Body",
  "Acromyrmex_charruanus"   = "A. charruanus"
)

# infer / read Type from list_ant robustly
type_col_ant <- intersect(names(list_ant), c("Type", "TYPE", "type"))
if (length(type_col_ant) == 0) {
  tmp_txt <- list_ant %>%
    mutate(.row = row_number()) %>%
    mutate(across(everything(), ~ as.character(.x)))

  inferred <- tmp_txt %>%
    pivot_longer(-.row, names_to = "col", values_to = "val") %>%
    mutate(val_l = str_to_lower(val)) %>%
    group_by(.row) %>%
    summarise(
      Type = case_when(
        any(str_detect(val_l, "receptor|gpcr|rhodopsin")) ~ "Receptor",
        any(str_detect(val_l, "neuropeptide|peptide"))   ~ "Neuropeptide",
        TRUE ~ NA_character_
      ),
      .groups = "drop"
    )

  list_ant2 <- tmp_txt %>%
    dplyr::select(.row, everything()) %>%
    left_join(inferred, by = ".row") %>%
    dplyr::select(-.row)
} else {
  list_ant2 <- list_ant %>%
    rename(Type = all_of(type_col_ant[1]))
}

list_ant2 <- list_ant2 %>%
  mutate(
    gene_base = str_extract(as.character(V2), "Tnyl_g\\d+"),
    Type = case_when(
      str_detect(str_to_lower(Type), "receptor|recett") ~ "Receptor",
      str_detect(str_to_lower(Type), "neuropeptide|peptide|neuropept") ~ "Neuropeptide",
      TRUE ~ Type
    )
  ) %>%
  filter(!is.na(gene_base))

og_gene_map_ant <- Ortho_ant %>%
  dplyr::select(Orthogroup, all_of(gene_col_ant)) %>%
  separate_rows(all_of(gene_col_ant), sep = ",\\s*") %>%
  mutate(gene_base = str_extract(.data[[gene_col_ant]], "Tnyl_g\\d+")) %>%
  filter(!is.na(gene_base)) %>%
  distinct(Orthogroup, gene_base)

og_type_ant <- og_gene_map_ant %>%
  inner_join(list_ant2 %>% dplyr::select(gene_base, Type), by = "gene_base") %>%
  distinct(Orthogroup, Type) %>%
  group_by(Orthogroup) %>%
  summarise(
    Type = if (n_distinct(Type) == 1) first(Type) else "Unknown",
    .groups = "drop"
  )

df_ant <- a_joined_ant %>% dplyr::select(-2)
species_cols_ant <- setdiff(names(df_ant), "Orthogroup")

df_plot_ant <- df_ant %>%
  pivot_longer(
    cols = all_of(species_cols_ant),
    names_to = "Species",
    values_to = "Count",
    values_transform = list(Count = ~ suppressWarnings(as.numeric(.x)))
  ) %>%
  mutate(
    Species = Species %>%
      str_replace("^X\\.[AB]\\.", "") %>%
      str_replace("^X\\.", ""),
    Count = replace_na(Count, 0),
    Count_log = log10(Count + 1)
  ) %>%
  left_join(og_type_ant, by = "Orthogroup") %>%
  mutate(
    Type = replace_na(Type, "Unknown"),
    Type = factor(Type, levels = c("Neuropeptide", "Receptor", "Unknown"))
  )

og_order_ant <- df_plot_ant %>%
  distinct(Orthogroup, Type) %>%
  arrange(Type, Orthogroup) %>%
  pull(Orthogroup)

df_plot_ant <- df_plot_ant %>%
  mutate(Orthogroup = factor(Orthogroup, levels = og_order_ant))

# italic labels (ANTS)
species_labels_ant_expr <- lapply(species_labels_ant, function(x) bquote(italic(.(x))))

# =========================
# CESTODE
# =========================

list_cestode <- read.csv("C:/Users/Startklar/Desktop/Neuropeptide_Paper/List_for_R_cestode.csv", header = FALSE)

Ortho_cest <- read.delim2(
  "C:/Users/Startklar/Desktop/Neuropeptide_Paper/Orthogrops_cestode_complete.txt",
  stringsAsFactors = FALSE,
  check.names = FALSE
)

gene_col_cest <- "proteins_from_cestode_genome"
targets_cest  <- unique(as.character(list_cestode$V2))

Ortho_hits_long_cest <- Ortho_cest %>%
  dplyr::select(Orthogroup, all_of(gene_col_cest)) %>%
  separate_rows(.data[[gene_col_cest]], sep = ",\\s*") %>%
  mutate(
    token = str_trim(.data[[gene_col_cest]]),
    gene_base = str_extract(token, "g\\d+\\.t\\d+")
  ) %>%
  filter(!is.na(gene_base), gene_base %in% targets_cest) %>%
  distinct(Orthogroup, gene_base, token)

Ortho_hits_cest <- Ortho_cest %>%
  semi_join(Ortho_hits_long_cest %>% distinct(Orthogroup), by = "Orthogroup")

cat("CESTODE | Orthogroups found:", length(unique(Ortho_hits_cest$Orthogroup)), "\n")
cat("CESTODE | Genes found:", length(unique(Ortho_hits_long_cest$gene_base)),
    "out of", length(targets_cest), "\n")
print(setdiff(targets_cest, unique(Ortho_hits_long_cest$gene_base)))

a_cest <- read.delim("C:/Users/Startklar/Desktop/Neuropeptide_Paper/Orthogroups.GeneCount_cestode.tsv")
a_cest <- a_cest[, -which(names(a_cest) %in% c("Total")), drop = FALSE]

a_joined_cest <- Ortho_hits_cest %>%
  left_join(a_cest, by = c("Orthogroup"))

# keep your original column drops
a_joined_cest <- a_joined_cest[, -(2:13)]
a_joined_cest <- a_joined_cest[, -(3)]

species_labels_cest <- c(
  "C.elegans"= "C. elegans",
  "D.latus" =  "D. latus",
  "E.canadensis" =  "E. canadensis",
  "E.granulosus" = "E. granulosus",
  "E.multiocularis" = "E. multiocularis",
  "H.diminuta" = "H. diminuta",
  "H.taeniaformis" = "H. taeniaformis",
  "M.corti" = "M. corti",
  "R.nana" = "R. nana",
  "S.solidus" = "S. solidus",
  "T.asiatica" = "T. asiatica",
  "T.nylanderi" = "T. nylanderi",
  "proteins_from_cestode_genome" = "A. brevis (annotation)",
  "transcripts.fasta.transdecoder_New_Cestode" = "Transdecoder A. brevis"
)

colnames(list_cestode) <- c("Type", "gene_base")

og_gene_map_cest <- Ortho_cest %>%
  dplyr::select(Orthogroup, all_of(gene_col_cest)) %>%
  separate_rows(all_of(gene_col_cest), sep = ",\\s*") %>%
  mutate(gene_base = str_extract(.data[[gene_col_cest]], "g\\d+\\.t\\d+")) %>%
  filter(!is.na(gene_base)) %>%
  distinct(Orthogroup, gene_base)

og_type_cest <- og_gene_map_cest %>%
  inner_join(list_cestode %>% dplyr::select(gene_base, Type), by = "gene_base") %>%
  distinct(Orthogroup, Type) %>%
  group_by(Orthogroup) %>%
  summarise(
    Type = if (n_distinct(Type) == 1) first(Type) else "Unknown",
    .groups = "drop"
  )

df_cest <- a_joined_cest %>% dplyr::select(-2)
species_cols_cest <- setdiff(names(df_cest), "Orthogroup")

df_plot_cest <- df_cest %>%
  pivot_longer(
    cols = all_of(species_cols_cest),
    names_to = "Species",
    values_to = "Count",
    values_transform = list(Count = ~ suppressWarnings(as.numeric(.x)))
  ) %>%
  mutate(
    Species = str_replace(Species, "\\.y$", ""),
    Count = replace_na(Count, 0),
    Count_log = log10(Count + 1)
  ) %>%
  left_join(og_type_cest, by = "Orthogroup") %>%
  mutate(
    Type = replace_na(Type, "Unknown"),
    Type = factor(Type, levels = c("Neuropeptide", "Receptor", "Unknown"))
  )

og_order_cest <- df_plot_cest %>%
  distinct(Orthogroup, Type) %>%
  arrange(Type, Orthogroup) %>%
  pull(Orthogroup)

df_plot_cest <- df_plot_cest %>%
  mutate(Orthogroup = factor(Orthogroup, levels = og_order_cest))

# italic labels (CESTODE)
species_labels_cest_expr <- lapply(species_labels_cest, function(x) bquote(italic(.(x))))

# =========================
# COMMON SCALE (same fill range for both; saved independently)
# =========================
rng_fill <- range(c(df_plot_ant$Count_log, df_plot_cest$Count_log), na.rm = TRUE)

# =========================
# PLOTS: ANTS (FLIPPED)
# =========================

p_heat_ant <- ggplot(df_plot_ant, aes(x = Orthogroup, y = Species, fill = Count_log)) +
  geom_tile() +
  scale_fill_gradient(
    low = "white",
    high = "darkblue",
    limits = rng_fill,
    oob = squish,
    name = expression(log[10](count + 1))
  ) +
  theme_bw() +
  theme(
    panel.grid = element_blank(),
    axis.title = element_blank(),
    axis.text.x = element_text(angle = 90, vjust = 0.5, hjust = 1, size = x_tick_size),
    axis.text.y = element_text(size = y_tick_size),
    legend.title = element_text(size = legend_title_size),
    legend.text  = element_text(size = legend_text_size)
  ) +
  scale_y_discrete(labels = species_labels_ant_expr)

p_type_ant <- df_plot_ant %>%
  distinct(Orthogroup, Type) %>%
  ggplot(aes(x = Orthogroup, y = 1, fill = Type)) +
  geom_tile() +
  scale_fill_manual(
    values = c(Neuropeptide = "#00CD66", Receptor = "#CD0000", Unknown = "grey80"),
    name = "OG type"
  ) +
  theme_void() +
  theme(
    legend.title = element_text(size = legend_title_size),
    legend.text  = element_text(size = legend_text_size)
  )

# type strip on TOP
p_final_ant <- p_type_ant / p_heat_ant + plot_layout(heights = c(1, 12))

# =========================
# PLOTS: CESTODE (FLIPPED)
# =========================

p_heat_cest <- ggplot(df_plot_cest, aes(x = Orthogroup, y = Species, fill = Count_log)) +
  geom_tile() +
  scale_fill_gradient(
    low = "white",
    high = "darkblue",
    limits = rng_fill,
    oob = squish,
    name = expression(log[10](count + 1))
  ) +
  theme_bw() +
  theme(
    panel.grid = element_blank(),
    axis.title = element_blank(),
    axis.text.x = element_text(angle = 90, vjust = 0.5, hjust = 1, size = x_tick_size),
    axis.text.y = element_text(size = y_tick_size),
    legend.title = element_text(size = legend_title_size),
    legend.text  = element_text(size = legend_text_size)
  ) +
  scale_y_discrete(labels = species_labels_cest_expr)

p_type_cest <- df_plot_cest %>%
  distinct(Orthogroup, Type) %>%
  ggplot(aes(x = Orthogroup, y = 1, fill = Type)) +
  geom_tile() +
  scale_fill_manual(
    values = c(Neuropeptide = "#00CD66", Receptor = "#CD0000", Unknown = "grey80"),
    name = "OG type"
  ) +
  theme_void() +
  theme(
    legend.title = element_text(size = legend_title_size),
    legend.text  = element_text(size = legend_text_size)
  )

# type strip on TOP
p_final_cest <- p_type_cest / p_heat_cest + plot_layout(heights = c(1, 12))

# =========================
# SAVE INDEPENDENTLY (NO COMBINED)
# =========================

# With Orthogroups on X, you usually need MORE WIDTH.
ggsave(
  filename = "C:/Users/Startklar/Downloads/heatmap_ANTS_flipped.pdf",
  plot = p_final_ant,
  width = 18,
  height = 12,
  dpi = 300
)

ggsave(
  filename = "C:/Users/Startklar/Downloads/heatmap_CESTODE_flipped.pdf",
  plot = p_final_cest,
  width = 18,
  height = 12,
  dpi = 300
)
```

**Neuropeptide-Receptrs Heatmaps (Supplementary Figures)**

```
# -----------------------------
# Settings
# -----------------------------
treat_order <- c("Uninfected", "Infected", "Queen")

ann_colors <- list(
  Treatment = c(
    "Queen"      = "#166B6B",
    "Uninfected" = "#7A7A7A",
    "Infected"   = "#F0E68C"
  )
)

# -----------------------------
# Get normalized counts directly from DESeq2
# -----------------------------
normalized_counts_Br <- counts(dds_Br, normalized = TRUE)
normalized_counts_FB <- counts(dds_FB, normalized = TRUE)

# -----------------------------
# Load candidate list
# V2 = gene ID
# V4 = acronym / label to show
# -----------------------------
candidates <- read.csv(
  "C:/Users/Startklar/Desktop/Neuropeptide_Paper/List_for_R.csv",
  header = FALSE,
  stringsAsFactors = FALSE
)

# neuropeptides
neuro_tbl <- candidates %>%
  dplyr::filter(grepl("neuropeptide", V3, ignore.case = TRUE)) %>%
  dplyr::transmute(
    gene_id = as.character(V2),
    label   = ifelse(is.na(V4) | V4 == "", as.character(V2), as.character(V4))
  ) %>%
  distinct(gene_id, .keep_all = TRUE)

# receptors
recep_tbl <- candidates %>%
  dplyr::filter(grepl("receptor", V3, ignore.case = TRUE)) %>%
  dplyr::transmute(
    gene_id = as.character(V2),
    label   = ifelse(is.na(V4) | V4 == "", as.character(V2), as.character(V4))
  ) %>%
  distinct(gene_id, .keep_all = TRUE)

# -----------------------------
# Reusable function
# -----------------------------
make_heatmap_plot <- function(norm_counts, dds, gene_tbl, panel_title = NULL,
                              show_colnames = FALSE) {

  mat <- as.matrix(norm_counts)
  storage.mode(mat) <- "numeric"

  if (is.null(rownames(mat))) {
    stop("norm_counts must have gene IDs as rownames.")
  }

  if (is.null(colnames(mat))) {
    stop("norm_counts must have sample names as colnames.")
  }

  # subset selected genes
  sel_genes <- intersect(rownames(mat), gene_tbl$gene_id)

  if (length(sel_genes) == 0) {
    stop(paste("No selected genes found in expression matrix for:", panel_title))
  }

  sel <- mat[sel_genes, , drop = FALSE]

  # metadata
  meta <- as.data.frame(SummarizedExperiment::colData(dds))

  if (!"Treatment" %in% colnames(meta)) {
    stop("colData(dds) must contain a column named 'Treatment'.")
  }

  if (is.null(rownames(meta))) {
    stop("colData(dds) must have sample names as rownames.")
  }

  meta$Treatment <- factor(meta$Treatment, levels = treat_order)

  # keep only shared samples
  common_samples <- intersect(colnames(sel), rownames(meta))

  if (length(common_samples) == 0) {
    stop(paste("No matching sample names between matrix and colData for:", panel_title))
  }

  sel  <- sel[, common_samples, drop = FALSE]
  meta <- meta[common_samples, , drop = FALSE]

  # order samples by treatment
  meta <- meta[order(meta$Treatment), , drop = FALSE]
  sel  <- sel[, rownames(meta), drop = FALSE]

  # remove genes with non-finite values
  keep_rows <- apply(sel, 1, function(x) all(is.finite(x)))
  sel <- sel[keep_rows, , drop = FALSE]

  if (nrow(sel) == 0) {
    stop(paste("No valid numeric genes left for:", panel_title))
  }

  # log2 normalized expression
  log_mat <- log2(sel + 1)

  # -----------------------------
  # min-max scaling per gene
  # each gene row goes from 0 to 1
  # -----------------------------
  row_min   <- apply(log_mat, 1, min, na.rm = TRUE)
  row_max   <- apply(log_mat, 1, max, na.rm = TRUE)
  row_range <- row_max - row_min

  safe_range <- row_range
  safe_range[safe_range == 0] <- 1

  scaled_mat <- sweep(log_mat, 1, row_min, "-")
  scaled_mat <- sweep(scaled_mat, 1, safe_range, "/")

  # flat genes -> midpoint
  scaled_mat[row_range == 0, ] <- 0.5

  # gene labels
  gene_match <- match(rownames(scaled_mat), gene_tbl$gene_id)
  row_labels <- gene_tbl$label[gene_match]
  row_labels[is.na(row_labels) | row_labels == ""] <- rownames(scaled_mat)[is.na(row_labels) | row_labels == ""]
  rownames(scaled_mat) <- make.unique(as.character(row_labels))

  # column annotation
  ann_col <- data.frame(Treatment = meta$Treatment)
  rownames(ann_col) <- rownames(meta)

  # gaps between treatment groups
  group_sizes <- table(meta$Treatment)
  group_sizes <- group_sizes[treat_order[treat_order %in% names(group_sizes)]]
  gaps_col <- cumsum(as.numeric(group_sizes))
  gaps_col <- gaps_col[gaps_col < ncol(scaled_mat)]

  # fixed color scale: 0 to 1
  breaks_seq <- seq(0, 1, length.out = 101)
  heat_cols  <- colorRampPalette(c("#313695", "white", "#A50026"))(100)

  # IMPORTANT: plot scaled_mat, not log_mat
  ph <- pheatmap(
    mat               = scaled_mat,
    color             = heat_cols,
    breaks            = breaks_seq,
    cluster_rows      = TRUE,
    cluster_cols      = FALSE,
    annotation_col    = ann_col,
    annotation_colors = ann_colors,
    gaps_col          = gaps_col,
    show_colnames     = show_colnames,
    show_rownames     = TRUE,
    fontsize_row      = 9,
    fontsize_col      = 8,
    fontsize          = 10,
    border_color      = NA,
    main              = panel_title,
    legend_breaks     = c(0, 0.5, 1),
    legend_labels     = c("0", "0.5", "1"),
    silent            = TRUE
  )

  ph$gtable
}

# -----------------------------
# Build heatmaps
# -----------------------------
p_Br_NP <- make_heatmap_plot(
  norm_counts   = normalized_counts_Br,
  dds           = dds_Br,
  gene_tbl      = neuro_tbl,
  panel_title   = "Brain - Neuropeptides",
  show_colnames = FALSE
)

p_Br_R <- make_heatmap_plot(
  norm_counts   = normalized_counts_Br,
  dds           = dds_Br,
  gene_tbl      = recep_tbl,
  panel_title   = "Brain - Receptors",
  show_colnames = FALSE
)

p_FB_NP <- make_heatmap_plot(
  norm_counts   = normalized_counts_FB,
  dds           = dds_FB,
  gene_tbl      = neuro_tbl,
  panel_title   = "Fat body - Neuropeptides",
  show_colnames = FALSE
)

p_FB_R <- make_heatmap_plot(
  norm_counts   = normalized_counts_FB,
  dds           = dds_FB,
  gene_tbl      = recep_tbl,
  panel_title   = "Fat body - Receptors",
  show_colnames = FALSE
)

# -----------------------------
# Show in R
# -----------------------------
grid.newpage()
grid.arrange(
  p_Br_NP, p_Br_R,
  p_FB_NP, p_FB_R,
  ncol = 2,
  nrow = 2
)

# -----------------------------
# Save as SVG
# -----------------------------
svg("gene_expression_heatmaps_log2_minmax_per_gene.svg", width = 18, height = 10)
grid.arrange(
  p_Br_NP, p_Br_R,
  p_FB_NP, p_FB_R,
  ncol = 2,
  nrow = 2
)
dev.off()
```

**CAPA and ITG Boxplots (Supplementary Figures)**

```
res_list <- list(
  Queen_vs_Uninfected_FB = results(dds_FB, contrast = c("Treatment", "Uninfected", "Queen")),
  Queen_vs_Uninfected_Br = results(dds_Br, contrast = c("Treatment", "Uninfected", "Queen")),
  Queen_vs_Infected_FB = results(dds_FB, contrast = c("Treatment", "Infected", "Queen")),
  Queen_vs_Infected_Br = results(dds_Br, contrast = c("Treatment", "Infected", "Queen")),
  Uninfected_vs_Infected_FB = results(dds_FB, contrast = c("Treatment", "Infected", "Uninfected")),
  Uninfected_vs_Infected_Br = results(dds_Br, contrast = c("Treatment", "Infected", "Uninfected"))
)

# Normalized counts for fat body
norm_counts_FB <- counts(dds_FB, normalized = TRUE)

# Normalized counts for brain
norm_counts_Br <- counts(dds_Br, normalized = TRUE)


#genes of interest
genes_Brain<-c("Tnyl_g05222", "Tnyl_g16971")
genes_FatBody<-c("Tnyl_g05222", "Tnyl_g16971")
#genes_FatBody <- c("Tnyl_g02946", "Tnyl_g13343", "Tnyl_g06350", "Tnyl_g06972", "Tnyl_g03690", "Tnyl_g15396", "Tnyl_g12751", "Tnyl_g10763")
#genes_Brain <- c("Tnyl_g13343", "Tnyl_g10971", "Tnyl_g06326", "Tnyl_g06326") #"Tnyl_g03511", "Tnyl_g15396")

gene_labels_brain <- c(
  # "Tnyl_g13343" = "<span style='font-weight:bold; font-size:14pt;'>Telomerase reverse transcriptase</span><br>(TERT, Tnyl_g13343)",
  # "Tnyl_g10971" = "<span style='font-weight:bold; font-size:14pt;'>Protein takeout-like</span><br>(TOL, Tnyl_g10971)",
  # "Tnyl_g06326" = "<span style='font-weight:bold; font-size:14pt;'>Apolipoprotein D</span><br>(ApoD, Tnyl_g06326)"
  # "Tnyl_g03511" = "<span style='font-weight:bold; font-size:14pt;'>transferrin</span><br>Tnyl_g03511",
  # "Tnyl_g15396" = "<span style='font-weight:bold; font-size:14pt;'>transferrin 1</span><br>Tnyl_g15396"
  "Tnyl_g05222" = "<span style='font-weight:bold; font-size:14pt;'>ITG</span><br>Tnyl_g05222",
  "Tnyl_g16971" = "<span style='font-weight:bold; font-size:14pt;'>CAPA</span><br>Tnyl_g16971"
  )

gene_labels_fb <- c(
  # "Tnyl_g13343" = "<span style='font-weight:bold; font-size:14pt;'>Telomerase reverse transcriptase</span><br>(TERT, Tnyl_g13343)",
  # "Tnyl_g02946" = "<span style='font-weight:bold; font-size:14pt;'>S-adenosylmethionine sensor upstream of mTORC1</span><br>(SAMTOR, Tnyl_g02946)",
  # "Tnyl_g06350" = "<span style='font-weight:bold; font-size:14pt;'>Protein takeout-like</span><br>(TOL,Tnyl_g06350)",
  # "Tnyl_g06972" = "<span style='font-weight:bold; font-size:14pt;'>Peroxiredoxin-like protein</span><br>(Prx-like, Tnyl_g06972)",
  # "Tnyl_g03690" = "<span style='font-weight:bold; font-size:14pt;'>Protein yellow</span><br>(Tnyl_g03690)",
  # "Tnyl_g15396" = "<span style='font-weight:bold; font-size:14pt;'>Transferrin</span><br>(TF,Tnyl_g15396)",
  # "Tnyl_g12751" = "<span style='font-weight:bold; font-size:14pt;'>X-ray repair cross-complementing protein 3</span><br>(XRCC3, Tnyl_g12751)",
  # "Tnyl_g10763" = "<span style='font-weight:bold; font-size:14pt;'>Epidermal growth factor receptor</span><br>(EGFR, Tnyl_g10763)"
  "Tnyl_g05222" = "<span style='font-weight:bold; font-size:14pt;'>ITG</span><br>Tnyl_g05222",
  "Tnyl_g16971" = "<span style='font-weight:bold; font-size:14pt;'>CAPA</span><br>Tnyl_g16971"
  )

## --------------------------------------------------------------------
## p-value -> stars
## --------------------------------------------------------------------
p_to_stars <- function(p) {
  if (is.na(p)) return(NA_character_)
  if (p <= 0.0001) return("****")
  if (p <= 0.001) return("***")
  if (p <= 0.01)  return("**")
  if (p <= 0.05)  return("*")
  return("")  # non significativo -> nessuna etichetta
}

## --------------------------------------------------------------------
## Significance data.frame: BRAIN
## --------------------------------------------------------------------
make_sig_df_brain <- function(genes, br_long, res_list) {
  
  range_y <- br_long |>
    mutate(norm_pos = ifelse(norm_count > 0, norm_count, NA)) |>
    group_by(gene) |>
    summarise(
      y_min = min(norm_pos, na.rm = TRUE),
      y_max = max(norm_pos, na.rm = TRUE),
      .groups = "drop"
    ) |>
    mutate(
      y_range = ifelse(
        is.finite(y_max - y_min) & (y_max - y_min) > 0,
        y_max - y_min,
        y_max * 0.2
      )
    )
  
  # comp order: Q–U (1), U–I (2), Q–I (3)
  sig_list <- list(
    data.frame(
      gene   = genes,
      group1 = "Queen",
      group2 = "Uninfected",
      padj   = res_list$Queen_vs_Uninfected_Br[genes, "padj"],
      comp   = 1L
    ),
    data.frame(
      gene   = genes,
      group1 = "Uninfected",
      group2 = "Infected",
      padj   = res_list$Uninfected_vs_Infected_Br[genes, "padj"],
      comp   = 2L
    ),
    data.frame(
      gene   = genes,
      group1 = "Queen",
      group2 = "Infected",
      padj   = res_list$Queen_vs_Infected_Br[genes, "padj"],
      comp   = 3L
    )
  )
  
  sig_df <- bind_rows(sig_list) |>
    mutate(
      label  = vapply(padj, p_to_stars, character(1)),
      group1 = factor(group1, levels = c("Queen", "Uninfected", "Infected")),
      group2 = factor(group2, levels = c("Queen", "Uninfected", "Infected"))
    ) |>
    left_join(range_y, by = "gene") |>
    mutate(
      y_raw      = y_max + 0.10 * y_range * comp,
      y_position = ifelse(is.finite(y_raw) & y_raw > 0,
                          log10(y_raw),
                          NA_real_)
    ) |>
    # tieni solo confronti con padj < 0.05 e posizioni valide
    filter(!is.na(y_position) & padj < 0.05)
  
  sig_df
}

## --------------------------------------------------------------------
## Significance data.frame: FAT BODY
## --------------------------------------------------------------------
make_sig_df_fb <- function(genes, fb_long, res_list) {
  
  range_y <- fb_long |>
    mutate(norm_pos = ifelse(norm_count > 0, norm_count, NA)) |>
    group_by(gene) |>
    summarise(
      y_min = min(norm_pos, na.rm = TRUE),
      y_max = max(norm_pos, na.rm = TRUE),
      .groups = "drop"
    ) |>
    mutate(
      y_range = ifelse(
        is.finite(y_max - y_min) & (y_max - y_min) > 0,
        y_max - y_min,
        y_max * 0.2
      )
    )
  
  # comp order: Q–U (1), U–I (2), Q–I (3)
  sig_list <- list(
    data.frame(
      gene   = genes,
      group1 = "Queen",
      group2 = "Uninfected",
      padj   = res_list$Queen_vs_Uninfected_FB[genes, "padj"],
      comp   = 1L
    ),
    data.frame(
      gene   = genes,
      group1 = "Uninfected",
      group2 = "Infected",
      padj   = res_list$Uninfected_vs_Infected_FB[genes, "padj"],
      comp   = 2L
    ),
    data.frame(
      gene   = genes,
      group1 = "Queen",
      group2 = "Infected",
      padj   = res_list$Queen_vs_Infected_FB[genes, "padj"],
      comp   = 3L
    )
  )
  
  sig_df <- bind_rows(sig_list) |>
    mutate(
      label  = vapply(padj, p_to_stars, character(1)),
      group1 = factor(group1, levels = c("Queen", "Uninfected", "Infected")),
      group2 = factor(group2, levels = c("Queen", "Uninfected", "Infected"))
    ) |>
    left_join(range_y, by = "gene") |>
    mutate(
      y_raw      = y_max + 0.10 * y_range * comp,
      y_position = ifelse(is.finite(y_raw) & y_raw > 0,
                          log10(y_raw),
                          NA_real_)
    ) |>
    filter(!is.na(y_position) & padj < 0.05)
  
  sig_df
}

## --------------------------------------------------------------------
## Build sig data.frames (assumes genes_Brain, genes_FatBody, br_long,
## fb_long, res_list already exist)
## --------------------------------------------------------------------
sample_info_br <- as.data.frame(colData(dds_Br)) |>
  rownames_to_column("sample")

sample_info_fb <- as.data.frame(colData(dds_FB)) |>
  rownames_to_column("sample")

## make sure Treatment has the right order
sample_info_br$Treatment <- factor(sample_info_br$Treatment,
                                   levels = c("Queen", "Uninfected", "Infected"))
sample_info_fb$Treatment <- factor(sample_info_fb$Treatment,
                                   levels = c("Queen", "Uninfected", "Infected"))

br_long <- norm_counts_Br[genes_Brain, , drop = FALSE] |>
  as.data.frame() |>
  rownames_to_column("gene") |>
  pivot_longer(
    cols      = -gene,
    names_to  = "sample",
    values_to = "norm_count"
  ) |>
  left_join(sample_info_br, by = "sample")

fb_long <- norm_counts_FB[genes_FatBody, , drop = FALSE] |>
  as.data.frame() |>
  rownames_to_column("gene") |>
  pivot_longer(
    cols      = -gene,
    names_to  = "sample",
    values_to = "norm_count"
  ) |>
  left_join(sample_info_fb, by = "sample")


sig_brain   <- make_sig_df_brain(genes_Brain,   br_long, res_list)
sig_fatbody <- make_sig_df_fb(genes_FatBody, fb_long,  res_list)

## --------------------------------------------------------------------
## Prepare data for plotting
## --------------------------------------------------------------------
br_long$group <- factor(br_long$Treatment,
                        levels = c("Queen", "Uninfected", "Infected"))
fb_long$group <- factor(fb_long$Treatment,
                        levels = c("Queen", "Uninfected", "Infected"))

br_long_plot <- br_long |> filter(gene %in% genes_Brain)
fb_long_plot <- fb_long |> filter(gene %in% genes_FatBody)

## --------------------------------------------------------------------
## Plots (assumes gene_labels_brain, gene_labels_fb, colors, base_theme)
## --------------------------------------------------------------------
sample_info_br <- as.data.frame(colData(dds_Br)) |>
  rownames_to_column("sample")

sample_info_fb <- as.data.frame(colData(dds_FB)) |>
  rownames_to_column("sample")

## make sure Treatment has the right order
sample_info_br$Treatment <- factor(sample_info_br$Treatment,
                                   levels = c("Queen", "Uninfected", "Infected"))
sample_info_fb$Treatment <- factor(sample_info_fb$Treatment,
                                   levels = c("Queen", "Uninfected", "Infected"))

p_brain <- ggplot(br_long_plot,
                  aes(x = group, y = norm_count, fill = group)) +
  geom_boxplot(outlier.shape = NA) +
  geom_jitter(width = 0.15, alpha = 0.6) +
  facet_wrap(~ gene, scales = "free_y",
             labeller = as_labeller(gene_labels_brain)) +
  scale_fill_manual(values = colors) +
  scale_x_discrete(limits=c("Uninfected","Infected","Queen")) +
  scale_y_continuous(trans = "log10") +
  labs(title = "Brain", y = "Normalized gene counts (log₁₀)") +
  theme_classic() +
  theme(
    strip.text = ggtext::element_markdown(), legend.position="none", axis.text.x=element_blank(),
    axis.ticks.x=element_blank(), axis.title.x = element_blank(), axis.text.y   = element_text(size = 14)) +
  ggsignif::geom_signif(
    data        = sig_brain,
    aes(xmin = group1, xmax = group2,
        annotations = label, y_position = y_position),
    manual      = TRUE,
    tip_length  = 0,
    textsize    = 5,
    vjust       = 0.3,
    inherit.aes = FALSE
  )

p_fatbody <- ggplot(fb_long_plot,
                    aes(x = group, y = norm_count, fill = group)) +
  geom_boxplot(outlier.shape = NA) +
  geom_jitter(width = 0.15, alpha = 0.6) +
  facet_wrap(~ gene, scales = "free_y",
             labeller = as_labeller(gene_labels_fb)) +
  scale_fill_manual(values = colors) +
  scale_y_continuous(trans = "log10") +
  scale_x_discrete(limits=c("Uninfected","Infected","Queen")) +
  labs(title = "Fat body", y = "Normalized gene counts (log₁₀)") +
  theme_classic() +
  theme(
    strip.text = ggtext::element_markdown(), legend.position="none", axis.text.x=element_blank(),
    axis.ticks.x=element_blank(), axis.title.x = element_blank(),  axis.text.y   = element_text(size = 14)  # <- important
  ) +
  ggsignif::geom_signif(
    data        = sig_fatbody,
    aes(xmin = group1, xmax = group2,
        annotations = label, y_position = y_position),
    manual      = TRUE,
    tip_length  = 0,
    textsize    = 5,
    vjust       = 0.3,
    inherit.aes = FALSE
  )

combined_plot <- p_brain / p_fatbody +
  plot_layout(heights = c(1, 1))

combined_plot

ggsave(
  "C:/Users/Startklar/Downloads/combined_brain_fatbody_CAPA_ITG.pdf",
  combined_plot,
  width = 10,
  height = 10
)
```
