# Supplementary material for: Cestode infection is linked to transcriptional shifts in neuropeptide signalling and caste-specific ageing pathways in a social insect
Source: BMC Genomics. 2026 Jun 15;27:547. doi: 10.1186/s12864-026-12959-6 (PMC13267302; doi:10.1186/s12864-026-12959-6)
Supplement: Supplementary file 9 — Supplementary Figs. 1. [file 12864_2026_12959_MOESM9_ESM.docx]

**Supplementary Figures**

**
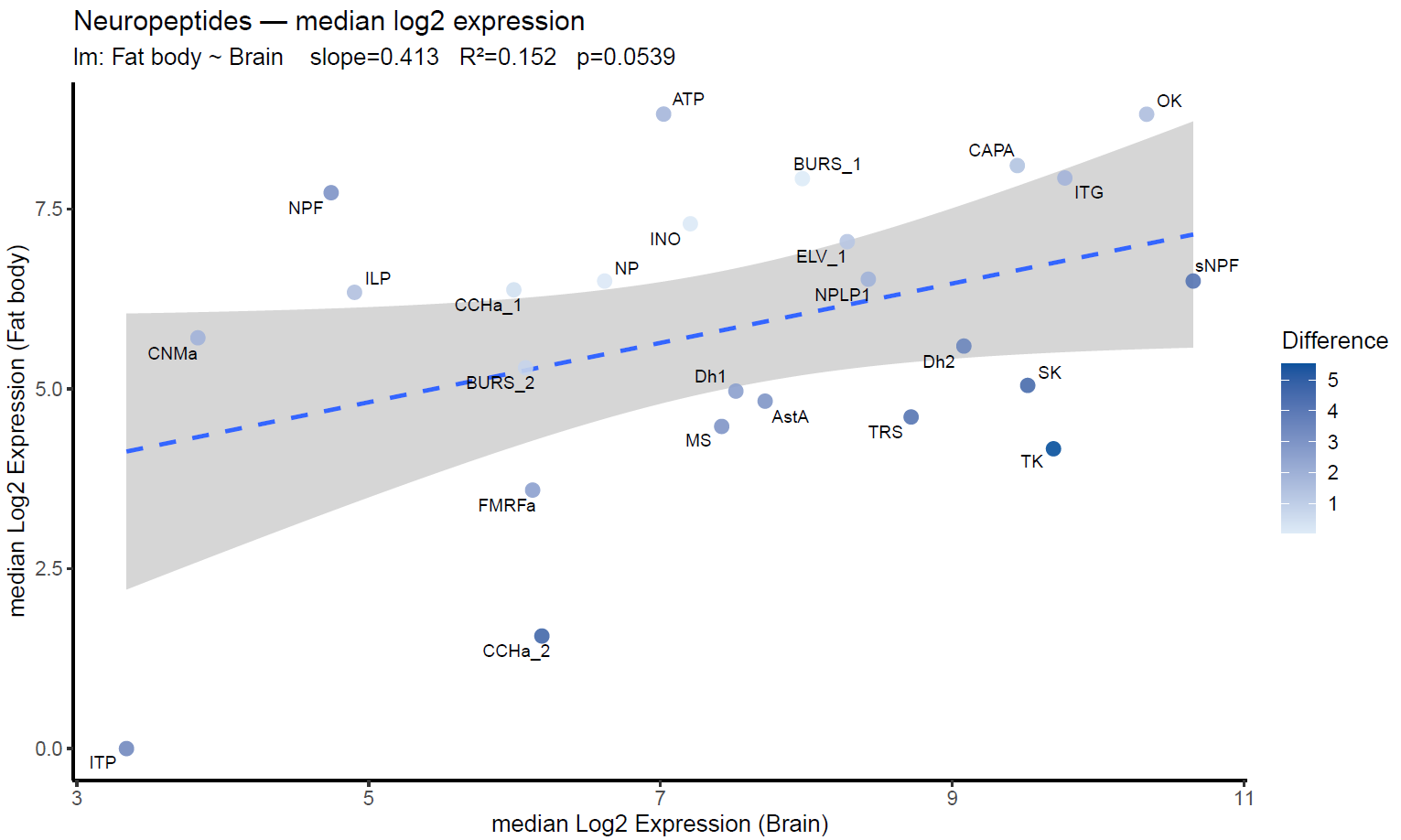
**

**Supplementary Figure 1. Median neuropeptide expression in brain and fat body.**

Scatterplot showing the median log2 expression of each annotated neuropeptide in the brain (x-axis) and fat body (y-axis) of *Temnothorax nylanderi*. Each point represents one neuropeptide and is coloured according to the absolute difference between its median expression in the brain and the fat body (darker colours indicate larger differences). The dashed blue line shows the linear regression (Fat body ~ Brain), with 95% confidence interval (grey shading). Labels denote peptide identities.


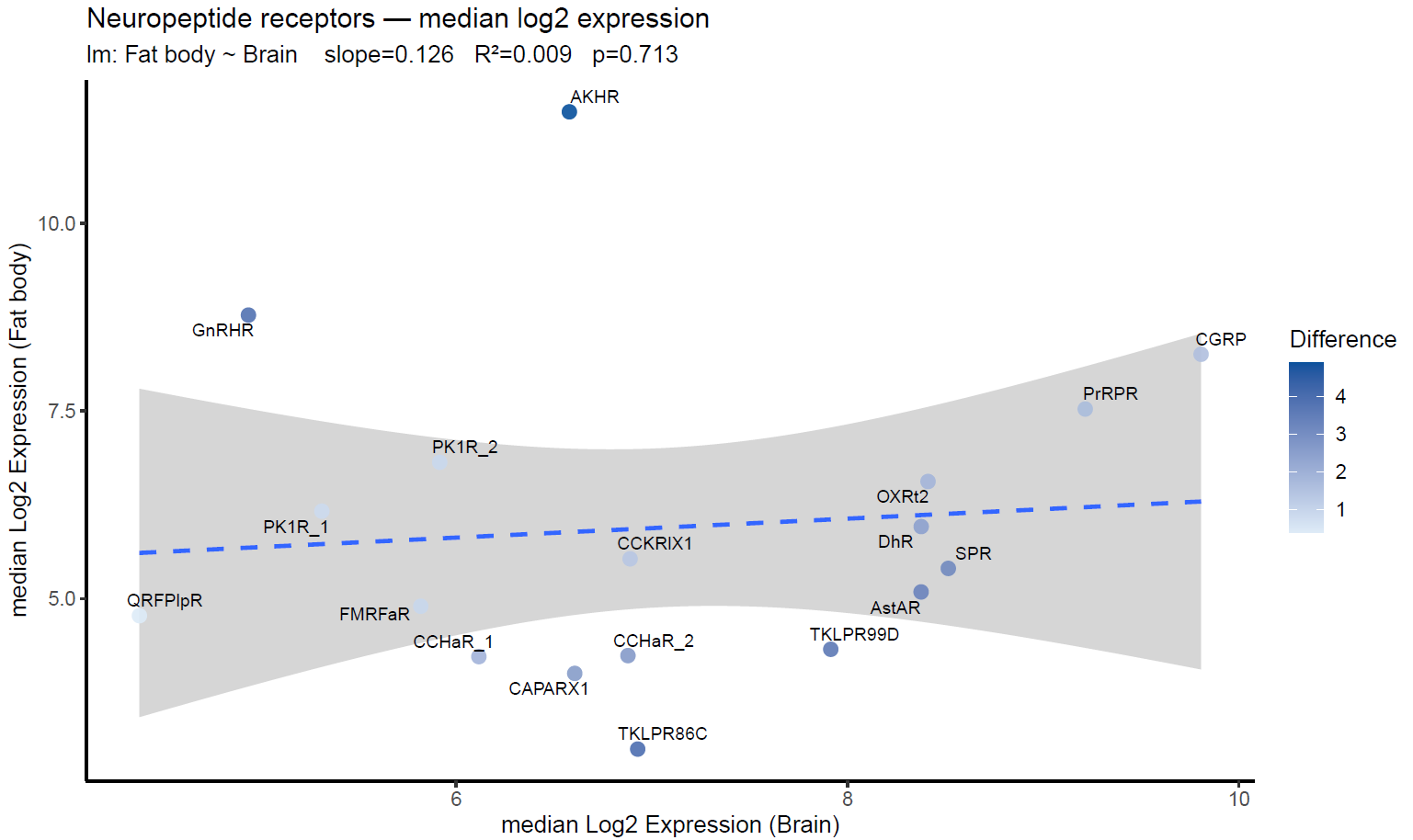


**Supplementary Figure 2. Median neuropeptide receptors expression in the brain and fat body.**

Scatterplot showing the median log2 expression of each annotated neuropeptide receptor in the brain (x-axis) and fat body (y-axis) of *T. nylanderi*. Each point represents one neuropeptide and is coloured according to the absolute difference between its median expression in the brain and the fat body (darker colours indicate larger differences). The dashed blue line shows the linear regression (Fat body ~ Brain), with 95% confidence interval (grey shading). Labels denote receptors identities.

**
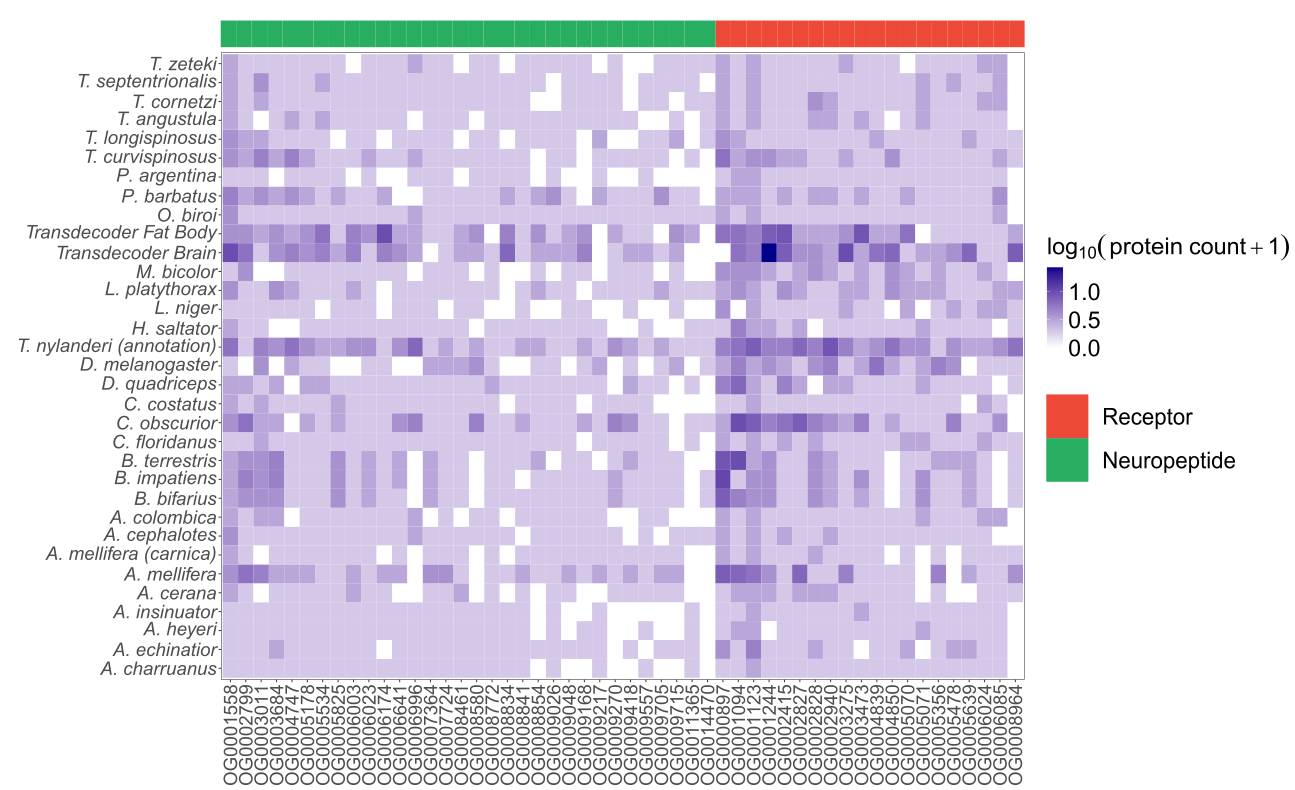
Supplementary Figure 3. Heatmap distribution of neuropeptide- and receptor-associated orthogroups across ant species and related social insects.**

Rows correspond to various species or transcriptome sources, while columns represent orthogroups (OGs). The colour of the cells indicates the log₁₀-transformed gene count number for each orthogroup (log₁₀[count + 1]), with darker violet shades signifying higher number of proteins. The topbar provides annotations for the orthogroup types, with green indicating neuropeptides and red representing receptors. Entries for *T. nylanderi* include genome annotations as well as tissue-specific transcriptomes obtained from TransDecoder, specifically for the brain and fat body.


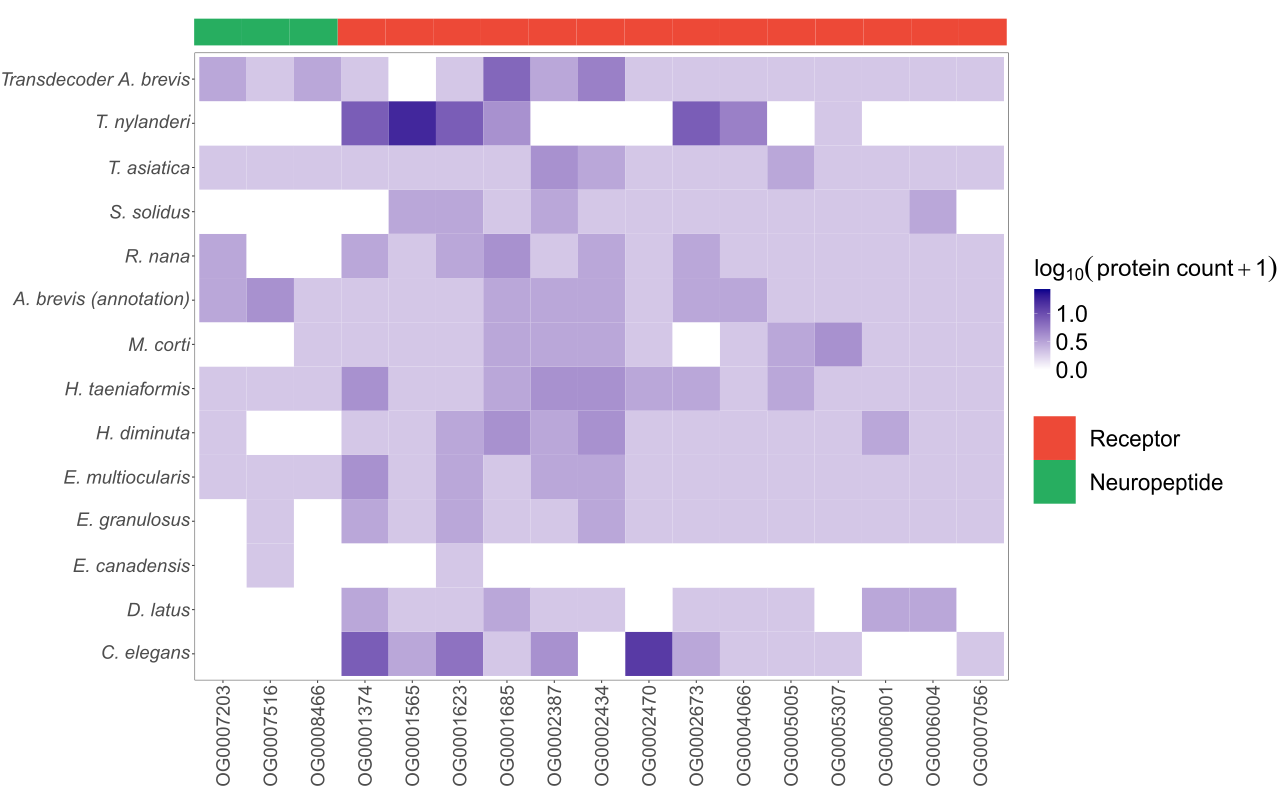


**Supplementary Figure 4. Heatmap distribution of neuropeptide- and receptor-associated orthogroups across *A. brevis* and cestodes.**

Rows correspond to various species or transcriptome sources, while columns represent orthogroups (OGs). Cell colour indicates the log₁₀-transformed gene count number per orthogroup (log₁₀[count + 1]), with darker shades indicating higher counts; white cells indicate absence. The topbar annotates orthogroup type (green: neuropeptides; red: receptors). In addition to cestode species, *Caenorhabditis elegans*, *T. nylanderi*, and *Anomotaenia brevis* annotation and TransDecoder-derived transcriptomes are shown for comparison.


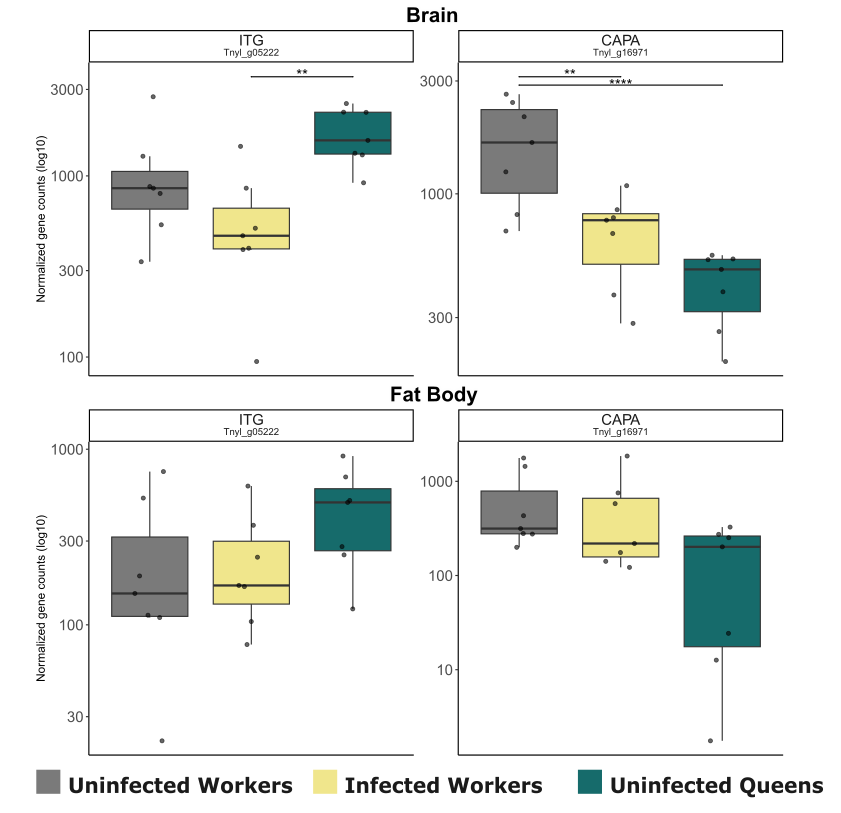


**Supplementary Figure 5.** Boxplots showing the expression of the newly functionally annotated neuropeptide genes *CAPA* and *ITG* in the brain and fat body of *T. nylanderi*.


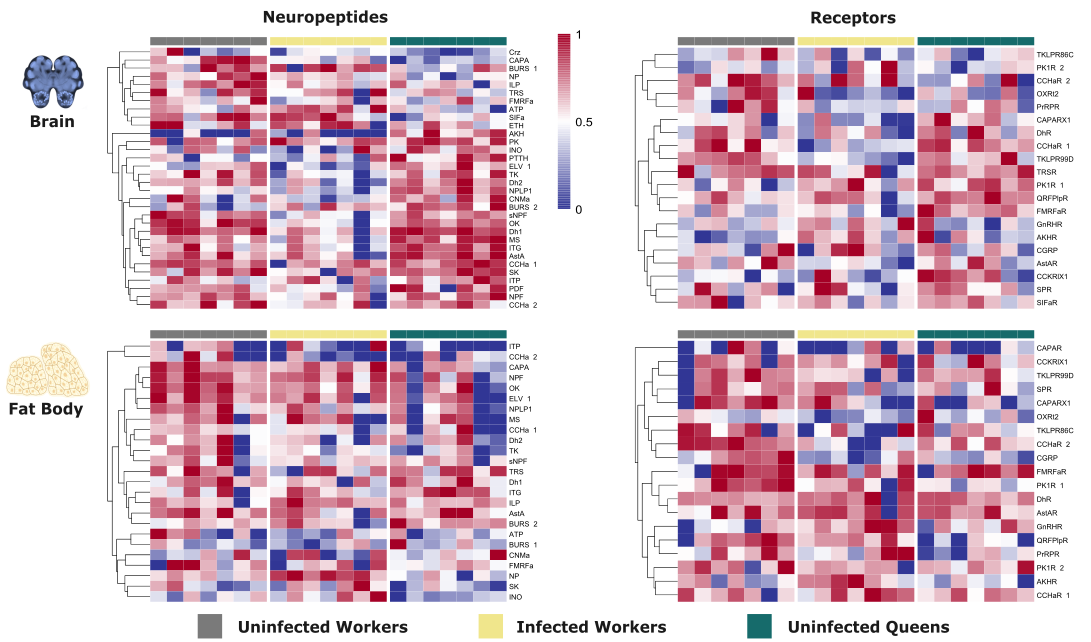


**Supplementary Figure 6. Heatmaps of the relative expression patterns of neuropeptide genes and neuropeptide receptor genes in the brain and fat body of *T. nylanderi* across uninfected workers, infected workers, and uninfected queens.**

Rows represent genes and columns represent individual samples. Samples are grouped by phenotype, indicated by the top annotation bar (grey: uninfected workers; yellow: infected workers; teal: uninfected queens). Gene expression is shown as relative scaled expression, with red indicating higher and blue lower expression. Rows were hierarchically clustered to highlight similarities in tissue- and phenotype-specific expression profiles.


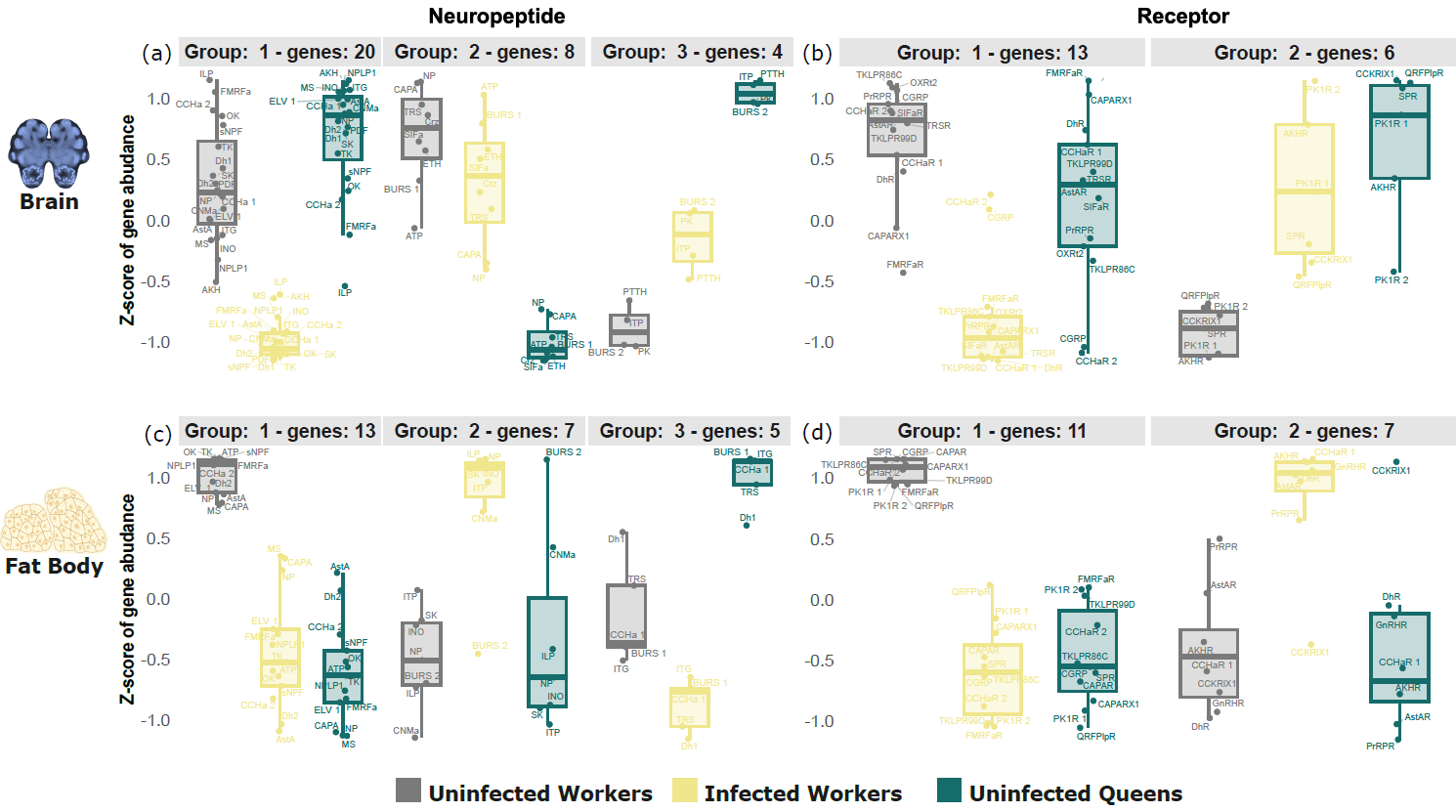


**Supplementary Figure 7. Expression profiles of neuropeptides and their receptors in the brain and fat body.**

Boxplots display clustered expression patterns of neuropeptides (a-c) and receptors (b-d) across castes and infection states in two tissues. Colours indicate caste and infection status: queens (teal), uninfected workers (grey), and infected workers (yellow). Labels indicate gene acronyms**.** Some graphical elements were created with BioRender.com.
